# MASP1‑METTL3‑HAS2 axis aggravates liver fibrosis through HMW‑HA‑mediated senescence inhibition and LMW‑HA‑induced activation of hepatic stellate cells

**DOI:** 10.1038/s41419-026-09158-2

**Published:** 2026-08-13

**Authors:** Xianzhi Liu, Peng Wang, Wei Zeng, Huajie Liu, Jun Lin, Wenxuan Gan, Meilian Yu, Yanyan Zheng, Qian Luo, Mingqing Zhang, Mingkai Li, Yihang Gong, Weiling He

**Affiliations:** 1 Department of Gastroenterology, Xiang’an Hospital of Xiamen University, School of Medicine, Xiamen University, Xiamen, China; 2 Department of Gastroenterology, the Third Affiliated Hospital, Sun Yat-sen University, Guangzhou, China; 3 Department of Pediatrics, Xiang’an Hospital of Xiamen University, School of Medicine, Xiamen University, Xiamen, China; 4 Department of Gastrointestinal Surgery, Xiang’an Hospital of Xiamen University, School of Medicine, Xiamen University, Xiamen, China; 5 Department of gastroenterology, Shenzhen Hospital of southern medical university, Shenzhen, China; 6 Biotherapy Centre & Cell-gene Therapy Translational Medicine Research Centre, the Third Affiliated Hospital, Sun Yat-sen University, Guangzhou, China

**Keywords:** Cell biology, Molecular biology

## Abstract

The hepatic microenvironment undergoes dynamic remodelling in advanced liver fibrosis. Hyaluronic acid (HA), a key component of this fibrotic microenvironment, plays a key role in disease progression. While cellular senescence is one of the fates that activated HSCs undergo and is associated with fibrosis resolution, the relationship between HA and HSC senescence in advanced liver fibrosis remains unclear. This study aimed to elucidate the specific mechanisms by which HA regulates HSC senescence. Here, intrahepatic HSC transplantation confirmed that an advanced fibrotic microenvironment inhibited HSC senescence and led to its sustained activation. Mechanistically, low-molecular-weight (LMW) HA promotes HSC activation via the CD44-p38-ATF2 axis, while HMW-HA simultaneously inhibits HSC senescence through the TLR4-SerpinE1 axis. Furthermore, HA synthesis is mediated by MASP1. MASP1 enhances the m6A methylation of HAS2 via METTL3, leading to increased HAS2 mRNA levels and consequently elevated HMW-HA synthesis, and HYAL2 mediates the conversion of HMW‑HA to LMW‑HA. Targeted knockdown of MASP1 in the liver reversed HA metabolism, subsequently promoting HSC senescence and alleviating liver fibrosis. Therefore, targeting HA metabolism to induce HSC senescence for activated HSC removal may represent a novel therapeutic strategy for liver fibrosis.

**Figure:**
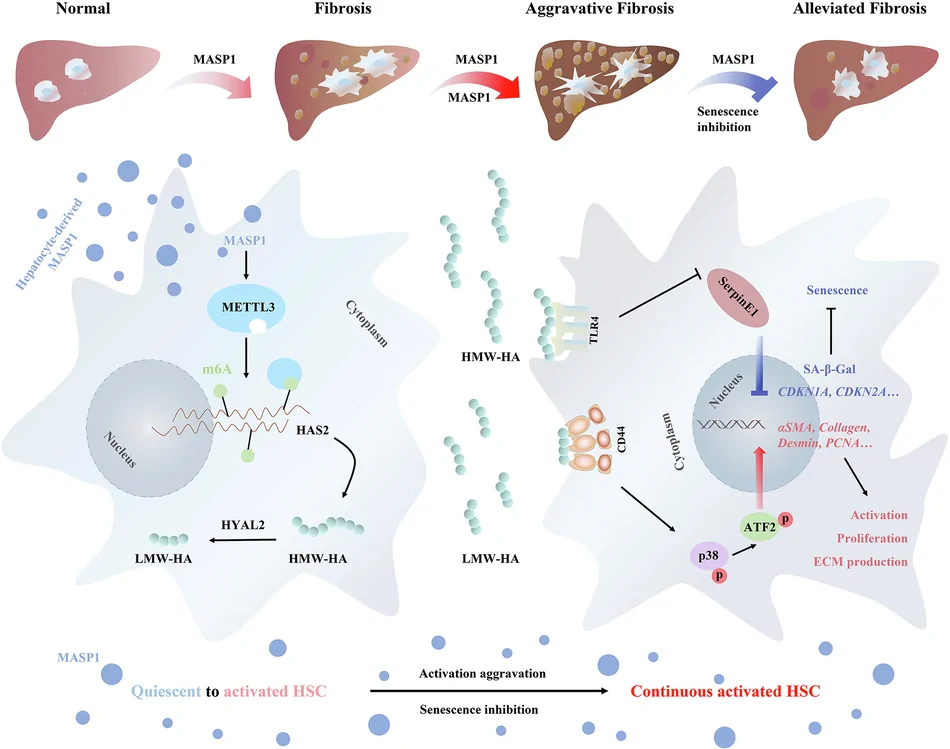

## Introduction

Cirrhosis is the end-stage outcome of various chronic liver diseases, including autoimmune liver disorders, metabolic dysfunction-associated liver disease, viral infections, and toxic injury [1]. Liver fibrosis, a reversible pathological stage, is characterized by the persistent overproduction of extracellular matrix. Myofibroblasts, the primary contributors to extracellular matrix deposition, originate mainly from HSCs [2]. The precise regulation of HSC activity is the focus of liver fibrosis treatment.

Following activation, HSCs can undergo several possible fates, including senescence, apoptosis, and inactivation. HSC senescence is characterized by reduced proliferation and collagen secretion, which halts further progression of liver fibrosis and is associated with its regression [3]. Senescent HSCs exhibit a range of associated phenotypes, such as enhanced senescence-associated β-galactosidase (SA-β‑Gal) activity, altered cell morphology, loss of DNA synthesis, and a proinflammatory secretory phenotype (senescence-associated secretory phenotype, SASP) [4, 5]. Senescent cells highly express two cell cycle inhibitory proteins, CDKN1A and CDKN2A, which serve as key biomarkers of senescence. How HSC senescence is regulated in liver fibrosis is largely unknown.

Hyaluronic acid (HA), a glycosaminoglycan, plays a crucial role in regulating cell-matrix interactions and maintaining liver architecture [6, 7]. HA in a healthy liver contributes to immune tolerance, tissue hydration, and parenchymal protection; however, under pathological stimuli such as metabolic disorders and alcohol consumption, HA is transformed into a driver of inflammation and carcinogenesis [8]. High-molecular-weight HA (HMW-HA) and low-molecular-weight HA (LMW-HA) have been reported to elicit divergent effects, thereby contributing to various liver diseases [9]. End-stage liver fibrosis is accompanied by a deficiency in repair capability [10–12], which is linked to alterations in the fibrotic microenvironment, and HA has been identified as a key regulator of this process. Therefore, how HA participates in the regulation of HSC activity in advanced liver fibrosis has become a key scientific issue.

In this study, we revealed that inhibition of HSC senescence is a key feature of advanced liver fibrosis. HA is markedly elevated in the advanced fibrotic microenvironment, where LMW-HA promotes HSC activation via the CD44-p38-ATF2 axis and HMW-HA suppresses HSC senescence through the TLR4-SerpinE1 axis, thereby aggravating liver fibrosis. Furthermore, the MASP1‑METTL3 axis was shown to increase HAS2 expression and HMW-HA synthesis through m^6^A modification, and HYAL2 mediates the conversion of HMW‑HA to LMW‑HA. Our findings demonstrate the inhibitory role of HA on HSC senescence to continuously activate HSCs in advanced liver fibrosis, offering a novel strategic direction for antifibrotic therapy.

## Materials and methods

### Human samples

Normal liver tissue samples were obtained from the para-haemangioma sites of patients with hepatic haemangioma, and liver fibrosis samples at different stages were obtained from corresponding patients before any therapeutic intervention. Human serum samples were obtained from healthy volunteers and patients with liver fibrosis. The study protocol was approved by the Clinical Research Ethics Committee of Xiang’an Hospital of Xiamen University.

### Mice and treatments

All animal experiments were approved by the Institutional Animal Care and Use Committee (IACUC) of Xiamen University and conducted in accordance with the NIH guidelines for the care and use of laboratory animals. Male C57BL/6 mice were used for all studies. To investigate the dynamic fate of activated HSCs (aHSCs) in evolving fibrotic microenvironments, a chronic liver fibrosis model was established. Mice received intraperitoneal (i.p.) injections of carbon tetrachloride (CCl_4_, diluted 1:4 in olive oil) at a dosage of 5 ml/kg body weight twice weekly for 12 weeks. At three strategic time points representing progressive fibrosis stages (week 3, week 7, and week 11), the mice were intrahepatically injected with TGF-β1-prestimulated and PKH26 fluorescent dye-labelled aHSCs. Liver tissues were collected, and PKH26-labelled aHSCs were isolated at week 4, 8, and 12. To evaluate the direct impact of MASP1 on fibrosis progression, a CCl_4_ model was used (i.p., 5 ml/kg, twice weekly). Concurrently, the mice were randomly assigned to receive one of the following treatments via tail vein injection: PBS control (equivalent volume, three times per week), recombinant MASP1 protein (0.5 µg of rMASP1 per mouse per injection, three times per week), or a MASP1-overexpressing luciferase plasmid (20 µg *MASP1-Luc* per mouse per injection). All the mice were sacrificed for sample collection. To delineate the distinct functions of low- and high-molecular-weight hyaluronic acid (LMW-HA and HMW-HA, respectively), mice were subjected to the CCl_4_ protocol. During this period, the mice received PBS control, LMW-HA, or HMW-HA. To assess the antifibrotic potential of inhibiting HA production, mice that were induced with CCl_4_ were treated with the HA synthesis inhibitor 4-methylumbelliferone (4-MU). To investigate the consequences of MASP1 inhibition in established fibrosis, at week 9, mice were administered one of the following adeno-associated virus (AAV) vectors via tail vein injection: AAV-shControl, AAV-shMASP1#1, or AAV-shMASP1#2. At week 11, all the mice received an intrahepatic injection of PKH26-labelled aHSCs. Finally, liver samples and labelled cells were collected at week 12 for molecular and cellular analyses. To knock down METTL3 in HSCs, *METTL3*^*flox/flox*^ mice were given one dose of adenovirus (Ad)-Des-CreERT2 or Ad-null. The mice were then injected with three doses of tamoxifen the following day for 3 consecutive days. AAV-OE-CDKN2A, AAV-sh-HAS2, and AAV-sh-HYAL2 were used separately to induce HSC senescence, inhibit HMW-HA synthesis, and HMW‑HA conversion to LMW‑HA. The above mice were combined with CCl_4_ treatment to establish the liver fibrosis model.

### Isolation of primary cells

Primary mouse hepatocytes and HSCs were harvested using an established two-step enzymatic portal vein perfusion protocol [13, 14]. Briefly, anaesthetized mice underwent in situ liver perfusion, initially with calcium-free Hank’s balanced salt solution (HBSS) for 15 min to clear the blood, followed by sequential digestion with 100 mL of 0.2% pronase and 0.2% collagenase type IV until the tissue softened and became pale. The digested liver was gently passed through a 100 μm nylon mesh filter. The resulting cell suspension was centrifuged at 50 × *g* to separate the cellular fractions. The pellet was collected for the isolation of hepatocytes, and the supernatant was then subjected to density gradient centrifugation for HSC isolation.

### RNA sequencing analysis

For transcriptomic profiling, cells were treated under experimental and control conditions for a specified duration. Total RNA was extracted using a standard phenol-chloroform-based reagent. RNA integrity and concentration were assessed by spectrophotometric and microfluidic analysis. Poly(A)+ mRNA was isolated via oligo(dT)-based magnetic bead purification. Sequencing libraries were prepared following a strand-specific protocol encompassing mRNA fragmentation, reverse transcription, end repair, adapter ligation, and PCR amplification. Libraries were quantified, and the size distribution was validated prior to high-throughput sequencing on an Illumina platform. Raw sequencing reads were processed to remove adapters and low-quality bases. Clean reads were then mapped to the appropriate reference genome using a splice-aware aligner. Transcript abundance was quantified and normalized to generate expression values (e.g., FPKM or TPM). Differential expression analysis was performed using a statistical model designed for count data, with significance thresholds defined by the fold-change magnitude and adjusted *p* value. Functional interpretation of the resulting gene lists was conducted through enrichment analysis against established biological pathway and gene ontology databases. For a systems-level perspective, gene set enrichment analysis (GSEA) was also performed to identify coordinated changes in predefined biological pathways.

### Cell culture and transfection

The human hepatic stellate cell line LX-2 was obtained from the ATCC, and authentication was performed by STR profiling. The LX-2 cell line tested negative for mycoplasma contamination. LX-2 cells were cultured in Dulbecco’s modified Eagle’s medium (DMEM) supplemented with 5% heat-inactivated foetal bovine serum (FBS). Cells were maintained at 37 °C in a humidified atmosphere containing 5% CO₂ and passaged at approximately 80% confluence using standard trypsinization protocols. For siRNA-mediated gene knockdown, cells were seeded in appropriate culture plates and transfected at 60–70% confluence using a lipid-based transfection reagent according to the manufacturer’s instructions. A nontargeting scrambled siRNA was used as a negative control. For plasmid transfection, cells were similarly transfected with the designated expression plasmids or an empty vector control. Following transfection, the cells were cultured for an additional 24–72 h before downstream analyses, such as RNA extraction, protein lysate preparation, or functional assays. Transfection efficiency was routinely monitored using a fluorescent control siRNA or a reporter plasmid.

### Statistical analysis

The data were statistically analysed using GraphPad Prism and are expressed as the mean ± SEM. The sample sizes and definitions of the statistical methods and measures for each experiment are provided in the corresponding figure legend. Statistical analysis was performed using Student’s *t* test or one-way ANOVA. A value of *P* < 0.05 was considered statistically significant.

The detailed materials and methods are included in the Supplementary Information.

## Results

### Reduced proportion of senescent hepatic stellate cells in advanced liver fibrosis

To investigate the dynamic changes in the proportion of senescent HSCs across different stages of liver fibrosis, liver samples were collected from patients with varying fibrosis grades. Immunofluorescence costaining revealed that the proportion of senescent HSCs (sHSCs, defined as Desmin⁺CDKN2A⁺) increased during the hepatitis and mild fibrosis stages but decreased during the severe cirrhosis stage. Furthermore, the proportions of quiescent HSCs (qHSCs, defined as Desmin⁺GFAP⁺) and inactivated HSCs (iHSCs, defined as Desmin⁺PPARγ⁺) decreased during fibrosis progression, whereas the proportion of apoptotic HSCs (apHSCs, defined as Desmin⁺Tunel⁺) increased (Fig. S1A, B). Consistent with these findings, both immunohistochemical staining and SA-β-Gal staining confirmed the reduction in senescent HSCs in cirrhosis (Fig. S1C). A CCl_4_-induced mouse model of liver fibrosis was subsequently established. Consistent with the above observations, the proportion of senescent HSCs decreased with advancing fibrosis stage (Fig. S1D–F and S2A–C). To further validate these findings, in vitro experiments were conducted using the human hepatic stellate cell line LX-2. Following stimulation with TGF-β1 in a concentration- and time-dependent manner, the expression of senescence-associated proteins (CDKN2A, CDKN1A, and IL6) initially increased but subsequently decreased. Concurrently, the expression of markers for qHSCs and iHSCs gradually decreased, whereas the expression of markers for aHSCs progressively increased (Fig. S3A, B).

**Fig. 1:**
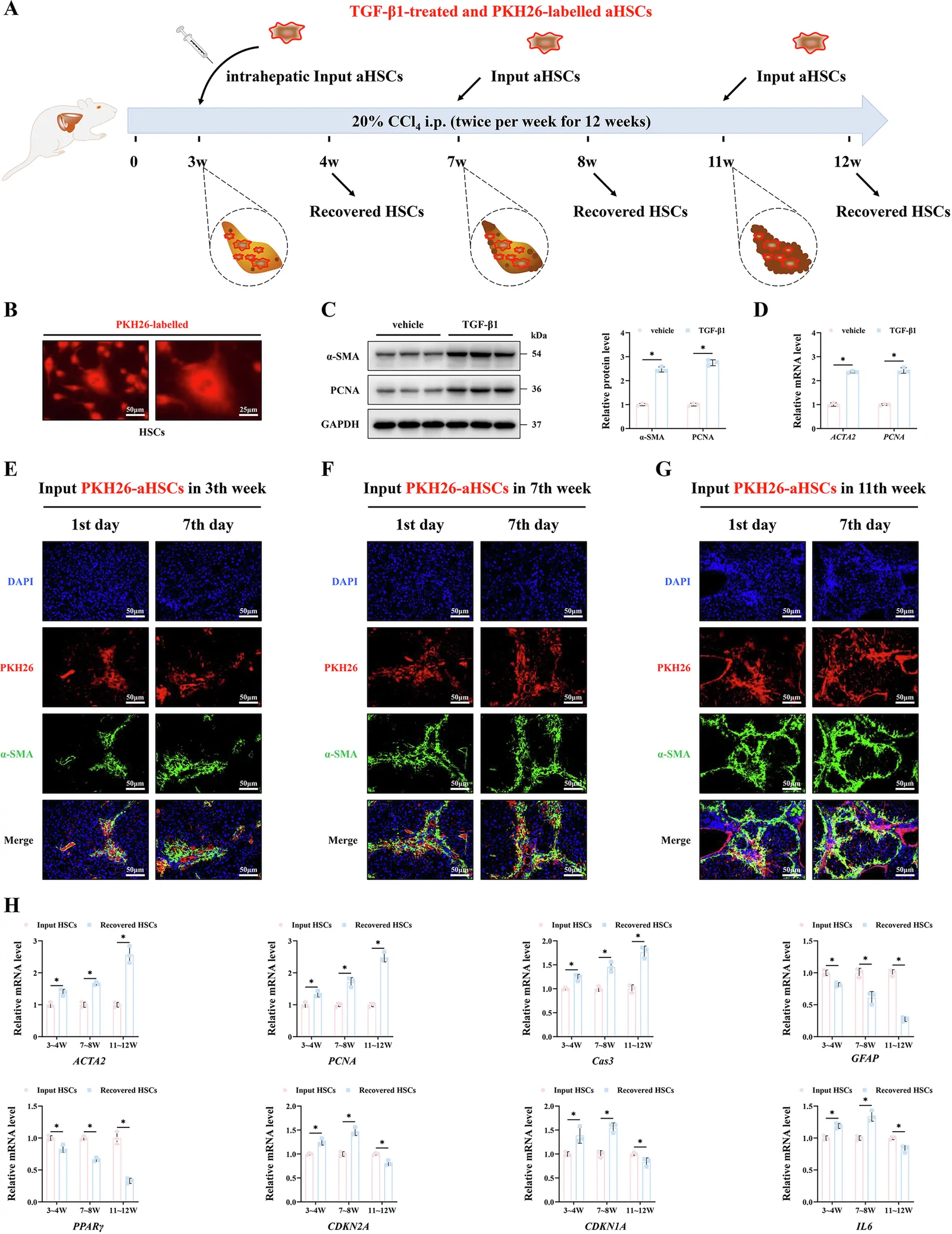
Analysis of HSC fate transformation during different fibrotic microenvironment. **A** Schematic diagram of the mouse modeling procedure. **B** PKH26 fluorescence staining in HSCs. Scale bar: 50 μm, 25 μm. **C**, **D** HSC activation induced by TGF‑β1; Data are presented as mean ± SEM; statistical significance was assessed by Student’s *t* test (*n* = 3 per group), *P < 0.05. **E**–**H** Effects of the fibrotic microenvironment at different stages on HSC fate assessed by qPCR and immunofluorescence co‑localization; data are presented as mean ± SEM; statistical significance was assessed by Student’s *t* test (**H**, *n* = 3 per group), *P < 0.05. Scale bar: 50 μm.

To investigate whether the reduction in the proportion of sHSCs is caused by the liver microenvironment in advanced liver fibrosis, we activated mouse primary HSCs in vitro using TGF-β1 and labelled them with PKH26. Subsequently, at different time points during CCl₄-induced modelling, PKH26-labelled activated HSCs were transplanted back into the mouse livers via intrahepatic injection, and mouse liver samples were collected 7 days later (Fig. 1A–D). Compared with the first two time points (week 3–4 and week 7–8), the microenvironment of advanced liver fibrosis (week 11–12) more readily activated HSCs, as indicated by fluorescence co-localization (Fig. 1E–G). We sorted PKH26-labelled HSCs from mouse livers at different stages of liver fibrosis. qPCR results revealed that the liver microenvironment in advanced fibrosis significantly activated HSCs and suppressed HSC senescence (Fig. 1H). Thus, the proportion of senescent HSCs was reduced in advanced liver fibrosis, which may be regulated by the irreversible fibrotic microenvironment.

### MASP1 increases in advanced liver fibrosis and enhances HSC activation by suppressing HSC senescence

**Fig. 2:**
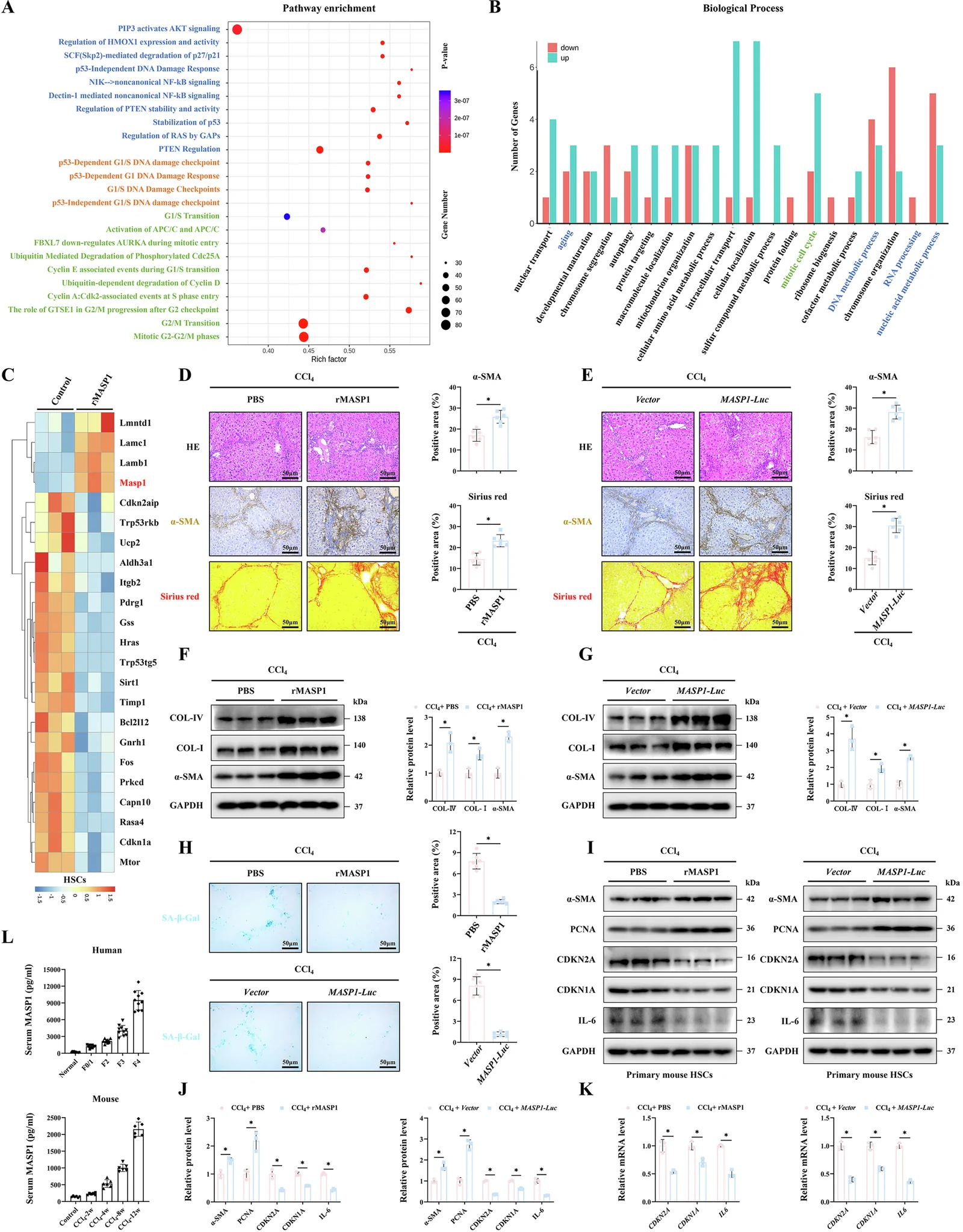
MASP1 inhibits HSC senescence and promotes liver fibrosis. **A**–**C** Transcriptome sequencing reveals the association of MASP1 with HSC senescence and activation. **D**–**G** MASP1 exacerbates liver fibrosis and injury in mice; Data are presented as mean ± SEM; statistical significance was assessed by Student’s *t* test (**D**, **E**, *n* = 6 per group; **F**, **G**, *n* = 3 per group), *P < 0.05. Scale bar: 50 μm. **H**–**K** MASP1 promotes HSC activation and suppresses the expression of senescence markers; Data are presented as mean ± SEM; statistical significance was assessed by Student’s *t* test (**H**, *n* = 6 per group; **I**–**K**, *n* = 3 per group), *P < 0.05. Scale bar: 50 μm. **L** MASP1 levels are significantly elevated in serum from patients and mice with advanced liver fibrosis. Data are presented as mean ± SEM (Human, *n* = 10 per group; Mouse, *n* = 6 per group).

Our previous research indicated that the expression of hepatocyte-derived MASP1 increases during liver fibrosis and promotes HSC activation [14]. Is the suppression of HSC senescence in advanced liver fibrosis mediated by MASP1? To further investigate the effect of MASP1 on HSCs, mouse primary HSCs were treated with recombinant MASP1 protein (rMASP1) and subjected to transcriptomic analysis. MASP1 treatment altered the gene expression profile of HSCs. KEGG and GO enrichment analyses revealed that many differentially expressed genes were associated with the cell cycle and cellular senescence (Fig. 2A, B). A heatmap demonstrated that MASP1 markedly suppressed the expression of senescence-associated genes (Fig. 2C). Subsequently, the mice were treated with rMASP1 or MASP1 overexpression plasmid (*MASP1-Luc*). H&E staining revealed that MASP1 exacerbated liver injury. Immunohistochemistry, Sirius Red staining, and protein assays indicated that MASP1 overexpression exacerbated liver fibrosis (Fig. 2D–G).

**Fig. 3:**
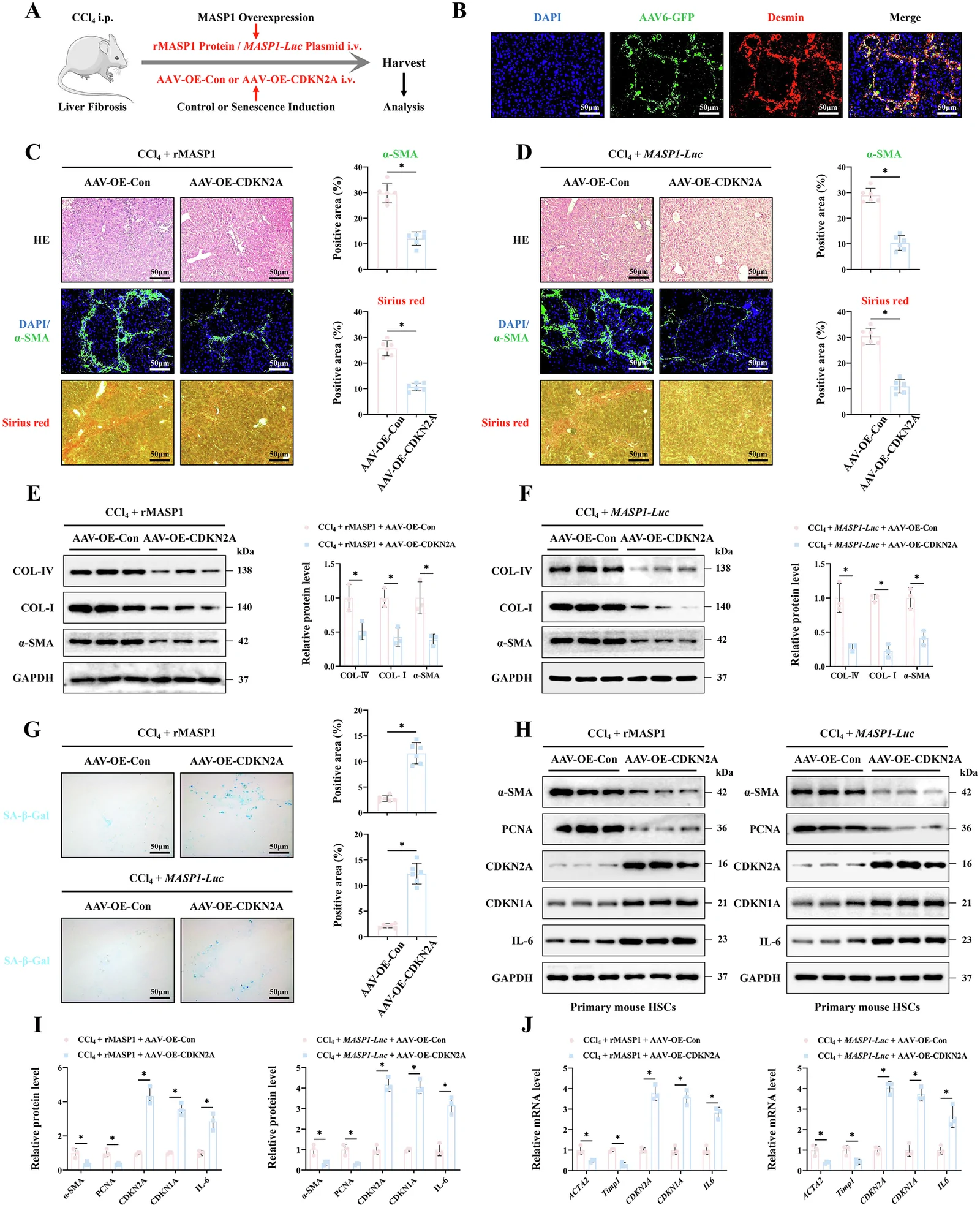
Targeted overexpression of CDKN2A in hepatic stellate cells reverses MASP1‑induced liver fibrosis. **A** Schematic diagram of the mouse model establishment. **B** Immunofluorescence detection of GFP-labeled AAV6 vector and HSC marker Desmin co-localization. Scale bar: 50 μm. **C**, **D** Mice were treated with rMASP1, MASP1‑Luc, and AAV‑OE‑CDKN2A; liver fibrosis was assessed by H&E, immunofluorescence, and Sirius Red staining. Data are presented as mean ± SEM; statistical significance was assessed by Student’s *t* test (*n* = 6 per group), *P < 0.05. Scale bar: 50 μm. **E**, **F** Western blot analysis of fibrosis‑related protein expression in mice treated with rMASP1, MASP1‑Luc, and AAV‑OE‑CDKN2A. Data are presented as mean ± SEM; statistical significance was assessed by Student’s *t* test (*n* = 3 per group), *P < 0.05. **G** SA‑β‑Gal staining for senescence detection in the above groups. Data are presented as mean ± SEM; statistical significance was assessed by Student’s *t* test (*n* = 6 per group), *P < 0.05. Scale bar: 50 μm. **H**–**J** Western blot and qPCR analysis of activation and senescence of primary HSCs isolated from mice in the AAV‑OE‑Con and AAV‑OE‑CDKN2A groups. Data are presented as mean ± SEM; statistical significance was assessed by Student’s *t* test (*n* = 3 per group), *P < 0.05.

We further examined whether MASP1 inhibits HSC senescence. SA-β-Gal staining revealed that MASP1 treatment suppressed cellular senescence (Fig. 2H). Both the mRNA and protein levels of CDKN2A, CDKN1A, and IL6 were lower in the MASP1-overexpressing group (Fig. 2I–K). Serum samples from patients and mice at different stages of liver fibrosis revealed that MASP1 expression increased to the greatest extent during advanced liver fibrosis (Fig. 2L). AAV6 was subsequently used to achieve targeted overexpression of CDKN2A in mouse hepatic stellate cells to induce senescence (Fig. 3A, B). Overexpression of the senescence‑associated protein CDKN2A significantly reversed MASP1‑induced liver injury and fibrosis (Fig. 3C, F). SA‑β‑Gal staining and results from primary mouse HSCs demonstrated that CDKN2A overexpression markedly promoted HSC senescence and reversed MASP1‑induced HSC activation (Fig. 3G–J). Furthermore, we explored the effects of MASP1-secreting hepatocytes from different stages of liver fibrosis on HSCs. Primary hepatocytes isolated from mice at different stages of liver fibrosis showed a marked increase in MASP1 secretion as fibrosis progressed, whereas si‑MASP1 treatment significantly reduced MASP1 secretion from hepatocytes (Fig. S4A). Coculture experiments further revealed that hepatocytes derived from both early‑stage and late‑stage fibrotic livers promoted HSC activation, whereas MASP1 knockdown reversed this effect. Notably, only hepatocytes from late‑stage fibrotic livers significantly inhibited HSC senescence, and this effect was reversed by si‑MASP1 treatment. These findings suggest that abundant MASP1 secretion in advanced liver fibrosis promotes HSC activation and suppresses HSC senescence (Fig. S4B–E). To further validate the above conclusions, we inhibited MASP1 expression in HSCs using *si-MASP1* and a C1 inhibitor. The reduction in MASP1 expression suppressed the activation and proliferation of HSCs (Fig. S5A–C). Furthermore, activated HSCs became more susceptible to senescence because of impaired MASP1 expression (Fig. S5D–F). A rescue experiment was subsequently performed by treating the cells with rMASP1 and MASP1-overexpressed plasmid. The overexpression of MASP1 reversed the HSC senescence induced by *si-MASP1* and the C1 inhibitor (Fig. S5G, H). These findings suggest that MASP1 upregulation in advanced liver fibrosis suppresses HSC senescence and exacerbates liver fibrosis.

### MASP1 promotes hepatic stellate cell activation and suppresses senescence by regulating hyaluronic acid metabolism

**Fig. 4:**
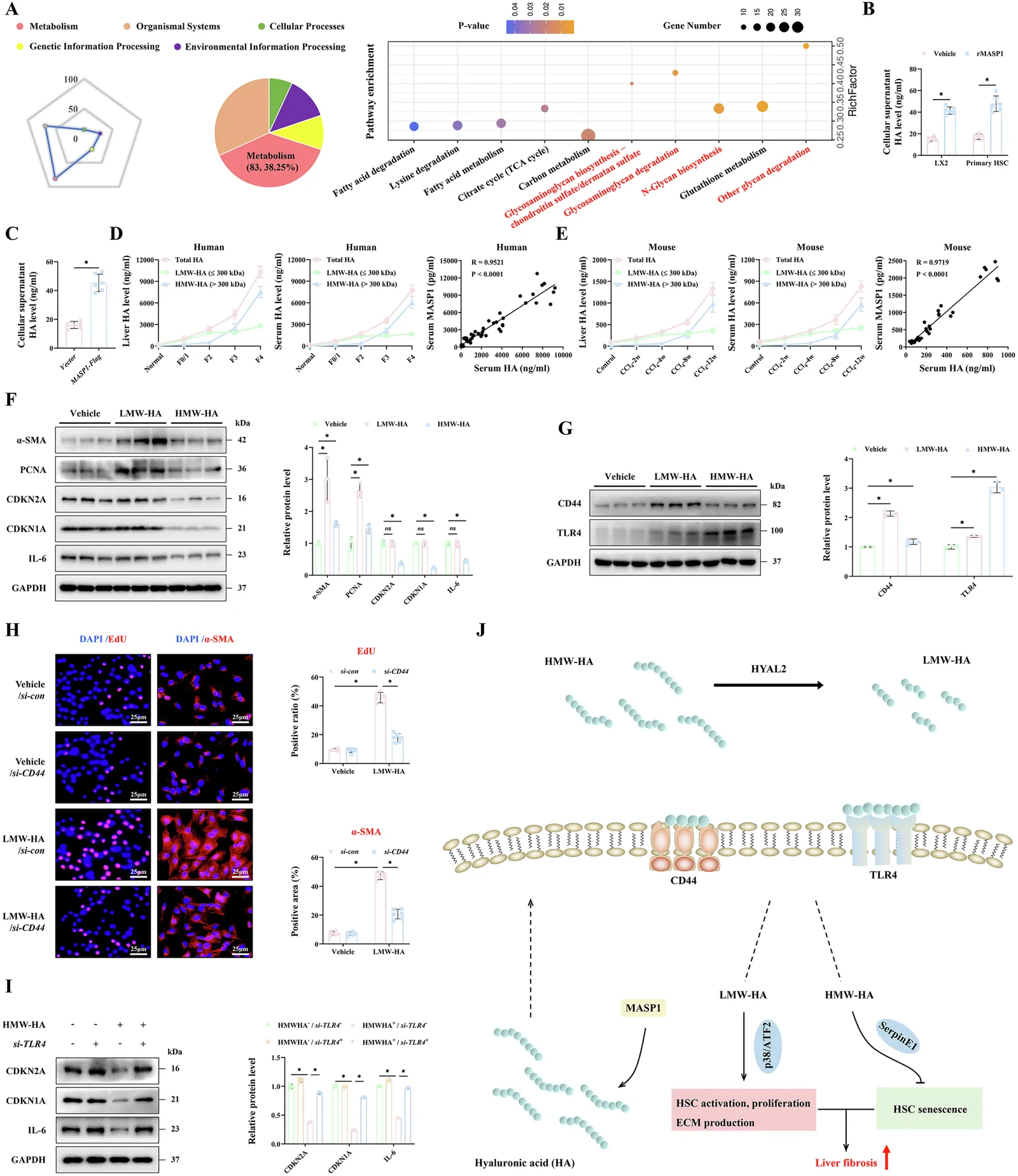
MASP1 promotes HSC activation and suppresses HSC senescence by regulating HA metabolism. **A** MASP1 is associated with glycosaminoglycan metabolism pathways. **B**, **C** MASP1 promotes HA secretion from HSCs; Data are presented as mean ± SEM; statistical significance was assessed by Student’s *t* test (*n* = 6 per group), *P < 0.05. **D**, **E** The levels of serum and liver total HA, HMW‑HA, LMW‑HA, and serum MASP1 were measured in mice and patients with liver fibrosis at different stages. Data are presented as mean ± SEM (Human, *n* = 10 per group; Mouse, *n* = 6 per group). And MASP1 level was positively correlated with HA level. **F**–**I** LMW-HA promotes HSC activation via CD44; HMW-HA suppresses HSC senescence via TLR4; Data are presented as mean ± SEM by one-way ANOVA (*n* = 3–6 per group), *P < 0.05. Scale bar: 25 μm. **J** Schematic diagram of the regulatory mechanism regarding HA in HSC activation and senescence.

To elucidate the mechanism by which MASP1 regulates HSC fate, differential gene KEGG enrichment analysis was performed. The results indicated that most altered pathways following MASP1 treatment were related to metabolic pathways, with several involved in glycosaminoglycan (GAG) metabolism (Fig. 4A). In the fibrotic liver microenvironment, the primary GAG secreted by HSCs is hyaluronic acid (HA). HSCs were then treated with rMASP1 and *MASP1-Flag*, and the increased MASP1 expression promoted HA synthesis in the supernatant (Fig. 4B, C). Furthermore, analysis of liver tissue and serum samples from patients with different stages of fibrosis revealed that the total HA content continuously increased as fibrosis progressed. HA can be classified by molecular weight into HMW-HA and LMW-HA [15, 16]. Further distribution analysis revealed that LMW-HA predominated in the early stages of liver fibrosis, whereas HMW-HA became the predominant form in advanced liver fibrosis. MASP1 levels were positively correlated with HA levels (Fig. 4D). These findings were also reproduced in mouse liver fibrosis samples (Fig. 4E). LMW-HA primarily promoted HSC activation, whereas HMW-HA inhibited HSC senescence (Fig. 4F). Given that TLR4 and CD44 have been reported to mediate HA-induced signalling [17], further assessment of HSCs revealed that LMW-HA treatment mainly upregulated CD44 expression, whereas HMW-HA treatment mainly upregulated TLR4 expression (Fig. 4G). CD44 knockdown reversed LMW-HA-induced HSC activation and proliferation (Fig. 4H), but si‑TLR4 did not significantly reverse LMW‑HA‑induced HSC activation (Fig. S6A). Additionally, TLR4 knockdown abolished the anti-senescence effect of HMW-HA (Fig. 4I), whereas si‑CD44 failed to reverse the HMW‑HA‑mediated inhibition of HSC senescence (Fig. S6B). Mechanistically, LMW-HA primarily binds to CD44, while HMW-HA primarily binds to TLR4 in activated HSCs (Fig. S6C, D).

As our previous study indicated that MASP1 activates the p38-ATF2 axis, we investigated whether the pro-activation effect of LMW-HA is mediated through this pathway. The inhibition of the p38 MAPK pathway by SB203580 impeded LMW-HA-induced HSC activation and proliferation (Fig. S7A–D). After HSCs were treated with TGF-β1 and the HA synthesis inhibitor 4-MU, transcriptomic analysis revealed that most functional alterations after HA synthesis inhibition were associated with cellular senescence, cell adhesion, and intercellular communication (Fig. S8A–D, S8G, H). A heatmap revealed upregulation of senescence-related genes and downregulation of activation-related genes in the 4-MU treatment group (Fig. S8E). A volcano plot revealed the greatest upregulation of serpin family E member 1 (SerpinE1) expression following 4-MU treatment (Fig. S8F). To further explore the mechanism by which HMW-HA inhibits senescence, we performed a transcriptomic study on HSCs treated with HMW-HA. The results demonstrated that the addition of HMW-HA downregulated the expression of senescence-related genes while upregulating the expression of genes related to HSC activation (Fig. S9E). Enrichment analysis indicated that most differentially expressed genes were functionally related to cellular senescence, intercellular communication, and collagen formation (Fig. S9A–D, S9G). SerpinE1 expression was most significantly downregulated after HMW-HA treatment (Fig. S9F). HSCs were subsequently transfected with a SerpinE1 overexpression plasmid. SerpinE1 overexpression reversed the suppression of senescence mediated by HMW-HA (Fig. S9H, I). Samples from patients and mice indicated that SerpinE1 levels increased during the mild liver fibrosis stage but decreased during advanced liver fibrosis (Fig. S9J). Accordingly, LMW-HA promotes HSC activation via the CD44-p38-ATF2 axis, whereas HMW-HA inhibits HSC senescence through the TLR4-SerpinE1 axis.

HYAL2 is a hyaluronidase involved in the hydrolysis of HMW‑HA to LMW‑HA [18]. We found that activated mouse primary HSCs highly expressed HAS2 and HYAL2, and that treatment of HSCs with rHYAL2 accelerated the conversion of HMW‑HA to LMW‑HA without altering the total HA content (Fig. S10A, B), verifying that HYAL2 is a key regulator in the hydrolysis of HMW‑HA to LMW‑HA. Furthermore, overexpression of HAS2 promoted the synthesis of HMW‑HA but had no effect on LMW‑HA synthesis, which can be explained by the fact that HAS2 overexpression did not affect HYAL2 expression (Fig. S10C, D). To further explain why the proportion of HMW‑HA is markedly increased in advanced liver fibrosis, we examined the expression of HAS2 and HYAL2 in primary HSCs from mice at different stages of fibrosis. Compared with that in the previous stage, HAS2 expression in advanced liver fibrosis was significantly elevated, whereas HYAL2 expression was not significantly increased (Fig. S10E). The overexpression of MASP1 significantly upregulated HAS2 expression but did not affect HYAL2 expression in activated-HSCs (Fig. S10F). These findings further demonstrate that in advanced liver fibrosis, abundant MASP1 secretion leads to increased HAS2 expression; however, the MASP1‑HAS2 axis does not influence HYAL2 expression in this stage. Consequently, the limited expression of HYAL2 prevents full processing of the large amount of HMW‑HA generated by the MASP1‑HAS2 axis, ultimately resulting in the accumulation of HMW‑HA, which significantly impedes the senescence of activated HSCs.

### Hyaluronic acid exacerbates liver fibrosis in vivo by promoting HSC activation and inhibiting senescence

**Fig. 5:**
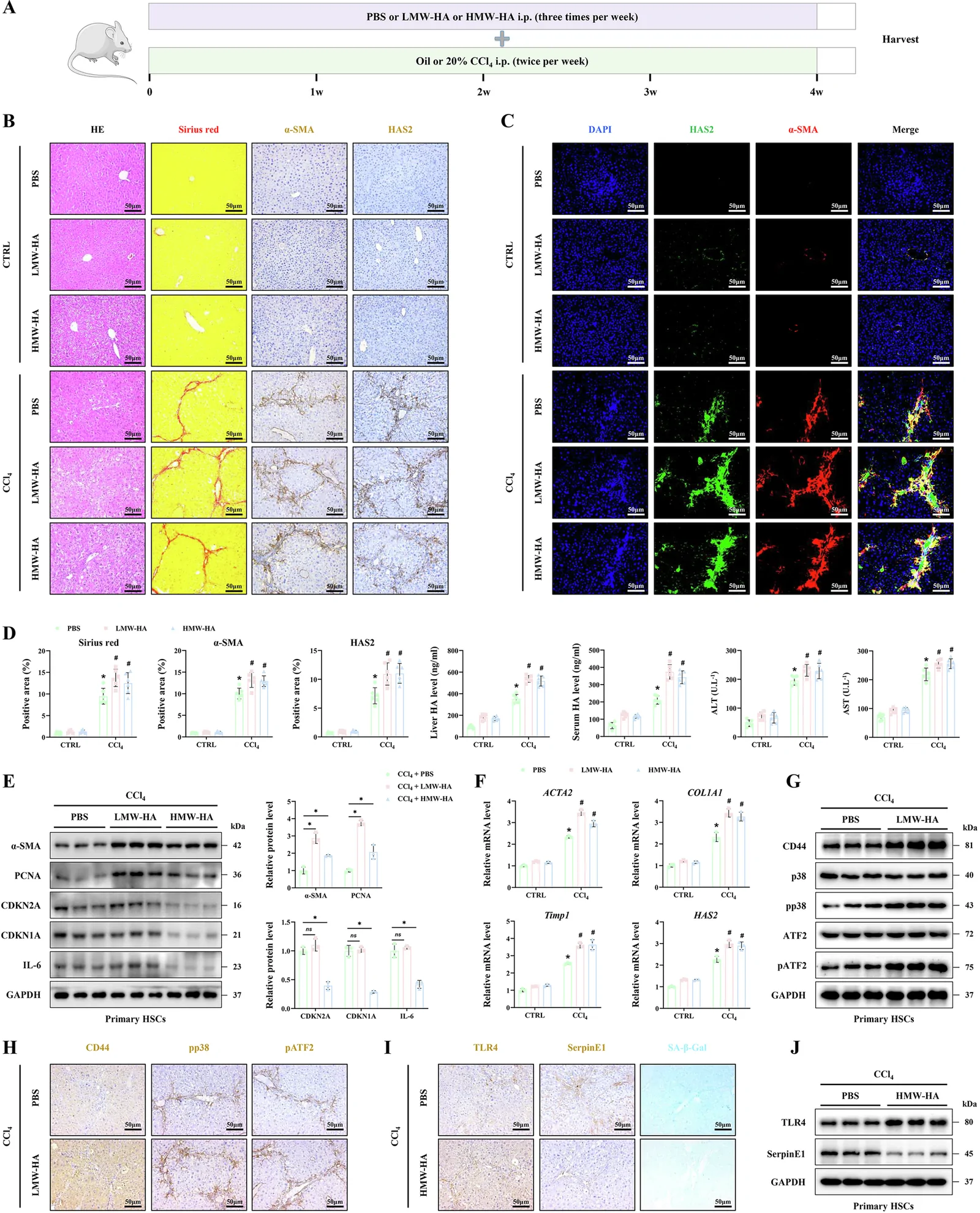
LMW-HA and HMW-HA exacerbate liver fibrosis by promoting HSC activation and suppressing HSC senescence, respectively. **A** Schematic diagram of the mouse modeling procedure. **B**–**D** LMW-HA and HMW-HA promote liver fibrosis and injury. Data are presented as mean ± SEM by one-way ANOVA (*n* = 6 per group), *P < 0.05 versus PBS/CTRL group, ^#^*P* < 0.05 versus PBS/CCl4 group. Scale bar: 50 μm. **E** LMW-HA and HMW-HA exacerbate liver fibrosis by promoting HSC activation and suppressing HSC senescence, respectively. Data are presented as mean ± SEM by one-way ANOVA (*n* = 3 per group), *P < 0.05. **F** LMW-HA and HMW-HA promote the mRNA levels of ACTA2, COL1A1, Timp1 and HAS2. Data are presented as mean ± SEM by one-way ANOVA (*n* = 3 per group), *P < 0.05 versus PBS/CTRL group, ^#^*P* < 0.05 versus PBS/CCl_4_ group. **G**–**J** LMW-HA and HMW-HA mediate their effects via the CD44-p38-ATF2 axis and the TLR4-SerpinE1 axis, respectively. *n* = 3–6 per group. Scale bar: 50 μm.

To clarify the functions of LMW-HA and HMW-HA, a mouse model of liver fibrosis was established (Fig. 5A). Both LMW-HA and HMW-HA exacerbated CCl_4_-induced liver fibrosis and injury, and the expression of hyaluronan synthase 2 (HAS2) increased as fibrosis worsened (Fig. 5B–D). Consistent with previous in vitro findings, western blotting revealed that LMW-HA primarily promoted HSC activation, whereas HMW-HA suppressed the expression of senescence-associated proteins (Fig. 5E). Both LMW-HA and HMW-HA also increased the mRNA levels of fibrotic markers and HAS2 (Fig. 5F). Studies on isolated mouse primary HSCs further verified that LMW-HA promoted HSC activation via the CD44-p38-ATF2 axis, whereas HMW-HA inhibited HSC senescence through the TLR4-SerpinE1 axis (Fig. 5G–J). The mice were subsequently treated with the HA synthesis inhibitor 4-MU (Fig. S11A). 4-MU treatment alleviated CCl_4_-induced liver injury and collagen deposition and reduced the expression of HAS2 (Fig. S11B–D). Experiments on mouse primary HSCs also revealed that 4-MU reduced HAS2 levels and HSC activation and promoted their senescence (Fig. S11E, F). Furthermore, 4-MU treatment suppressed the activity of both the CD44-p38-ATF2 axis and the TLR4-SerpinE1 axis (Fig. S11G–I). We also treated activated human HSCs (LX2 cells) with 4-MU and reported that 4-MU significantly reduced both the mRNA and protein expression of HAS2, promoted senescence, and inhibited the activation of HSCs (Fig. S11J, K). These findings further emphasize the role of HA in aggravating liver fibrosis through the regulation of HSC fate. Furthermore, to further elucidate our conclusions, we treated mice with AAV-sh-HAS2 or AAV-sh-HYAL2. The results revealed that knockdown of HAS2 significantly alleviated CCl₄-induced liver fibrosis in mice, whereas knockdown of HYAL2 had no significant effect (Fig. S12A). Moreover, HAS2 knockdown significantly promoted HSC senescence, whereas HYAL2 knockdown markedly inhibited HSC senescence (Fig. S12B, S12D). Mechanistically, knockdown of HAS2 significantly reduced the levels of total HA, HMW-HA, and LMW-HA, whereas knockdown of HYAL2 significantly increased HMW-HA levels and decreased LMW-HA levels but did not significantly affect the total HA content; in other words, knockdown of HYAL2 impeded the hydrolysis of HMW-HA to LMW-HA (Fig. S12C). Thus, HA exacerbates HSC activation and liver fibrosis by promoting HSC activation and inhibiting HSC senescence.

### MASP1 promotes HAS2 expression and HMW-HA synthesis via METTL3-mediated m^6^A modification

**Fig. 6:**
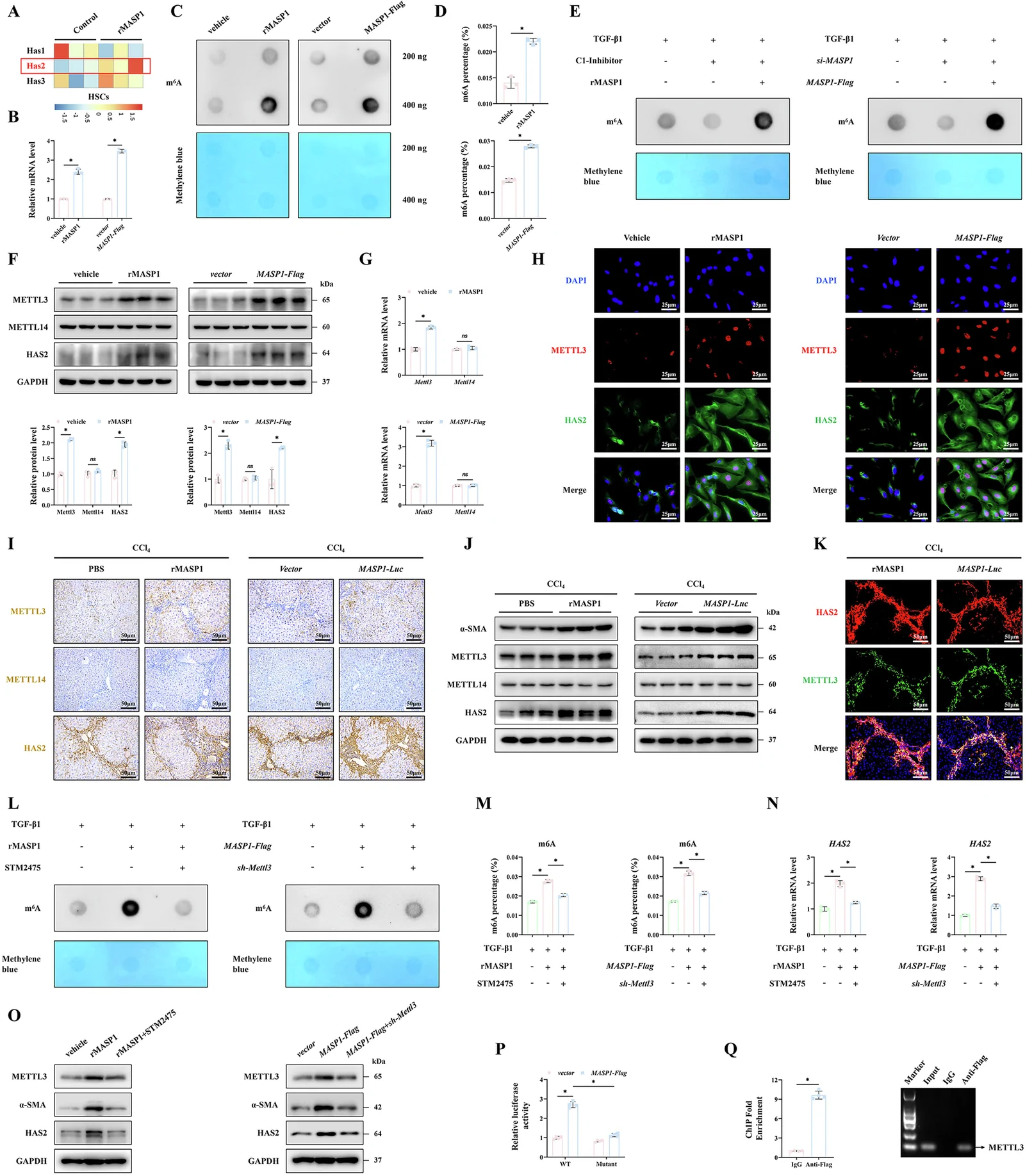
MASP1 promotes HAS2 expression through METTL3-mediated m^6^A modification. **A**, **B** MASP1 increases HAS2 mRNA expression. Data are presented as mean ± SEM; statistical significance was assessed by Student’s *t* test (*n* = 3 per group), *P < 0.05. **C**–**E** MASP1 elevates m^6^A methylation, while the MASP1 inhibitor C1-inhibitor and si-MASP1 can reverse this increase. Data are presented as mean ± SEM; statistical significance was assessed by Student’s *t* test or one-way ANOVA (*n* = 3 per group), *P < 0.05. **F**–**J** MASP1 promotes the expression of METTL3 and HAS2. Data are presented as mean ± SEM; statistical significance was assessed by Student’s *t* test (*n* = 3–6 per group), *P < 0.05. Scale bar: 25 μm. **K** Immunofluorescence staining shows co-localization of METTL3 and HAS2. Scale bar: 25 μm. **L**, **M** Inhibition of METTL3 reverses the MASP1-induced increase in m^6^A methylation. Data are presented as mean ± SEM by one-way ANOVA (*n* = 3 per group), *P < 0.05. **N**, **O** Inhibition of METTL3 reverses the MASP1-induced upregulation of HAS2 expression. Data are presented as mean ± SEM by one-way ANOVA (*n* = 3 per group), *P < 0.05. **P**, **Q** MASP1 binds to the promoter of METTL3. Data are presented as mean ± SEM; statistical significance was assessed by Student’s *t* test or one-way ANOVA (*n* = 3 per group), *P < 0.05.

As the key enzyme in HMW-HA synthesis, the level of HAS2 was significantly upregulated by rMASP1 treatment, whereas the expression of HAS1 and HAS3 did not significantly change according to the sequencing data (Fig. 6A). Overexpression of MASP1 markedly increased the mRNA level of HAS2 (Fig. 6B). Methylations are widely involved in the regulation of transcription and translation, and m^6^A methylation, as the most prevalent form, plays a crucial role in this process [19, 20]. Dot blot assays revealed that MASP1 overexpression increased m^6^A methylation (Fig. 6C, D), whereas C1 inhibitor treatment or MASP1 knockdown reduced m6A methylation (Fig. 6E). Further analysis revealed that MASP1 overexpression increased the mRNA and protein levels of METTL3, as a “Writer” of this process, but not that of METTL14 (Fig. 6F, G). Fluorescence confirmed that MASP1 enhanced the expression of both METTL3 and HAS2 in activated-HSCs (Fig. 6H). Immunostaining and western blot analysis revealed elevated-METTL3 and HAS2 expression in the MASP1-overexpressing fibrotic mice (Fig. 6I–K). To determine whether the MASP1-METTL3 axis affects HAS2 expression, HSCs were treated with the METTL3 inhibitor STM2475 or transfected with *sh-METTL3*. Both STM2475 and *sh-METTL3* reversed the increase in m^6^A methylation induced by MASP1 overexpression (Fig. 6L, M). The increase in HAS2 expression and HSC activation caused by MASP1 was also reversed by STM2475 and *sh-METTL3* (Fig. 6N, O). Subsequent luciferase reporter and ChIP assays demonstrated that MASP1 could bind to METTL3 DNA (Fig. 6P, Q). Taken together, these data indicate that METTL3-mediated m^6^A modification is important for MASP1-regulated HAS2 expression and HMW-HA synthesis, which contributes to HSC activation.

### Hepatic stellate cell-specific deficiency of METTL3 alleviates HSC activation and liver fibrosis

**Fig. 7:**
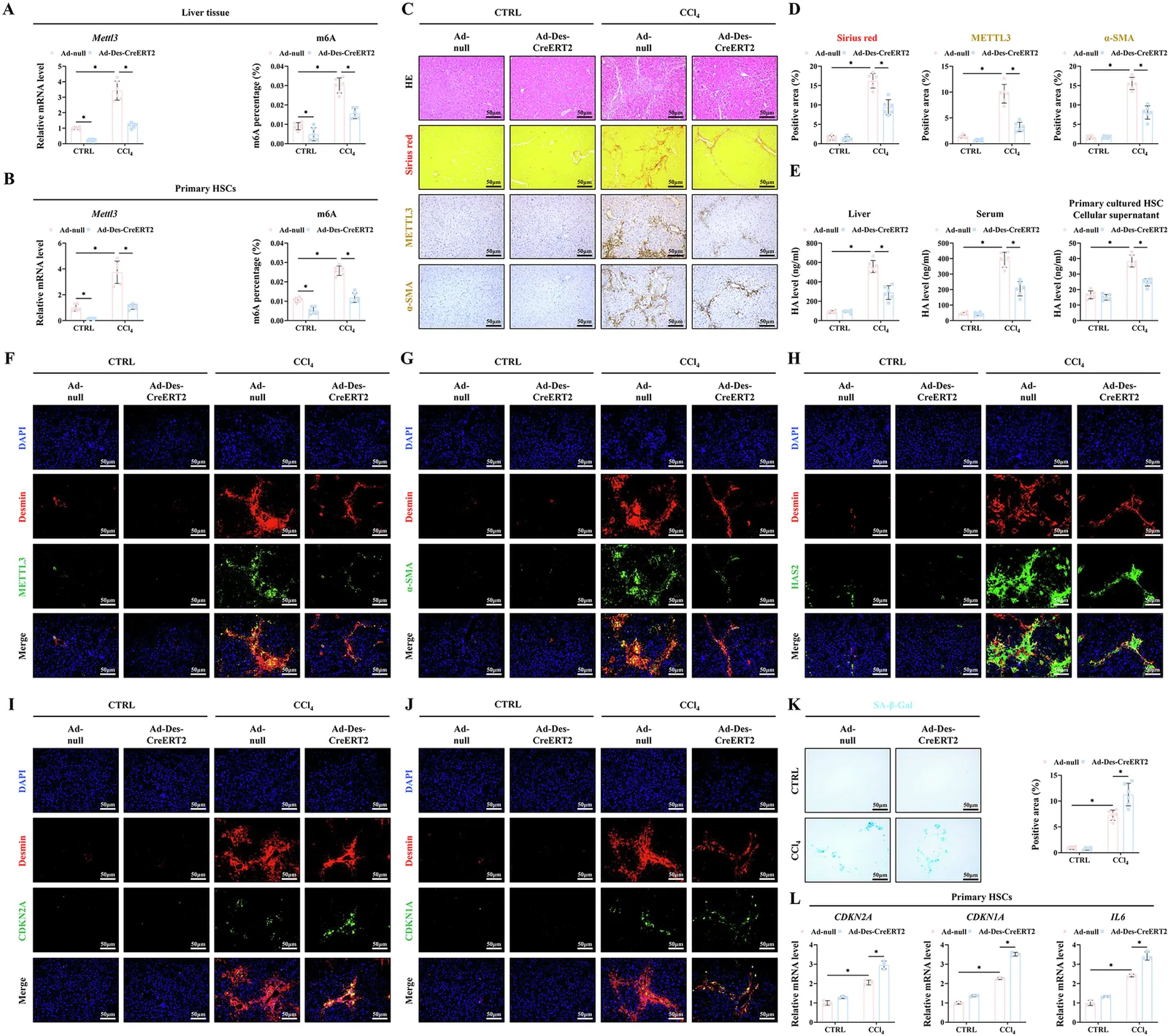
HSC-specific deficiency of METTL3 alleviates liver fibrosis by modulating HSC activation and senescence via the m^6^A/HAS2/HA pathway. **A**, **B** METTL3 deficiency reduces m^6^A methylation levels. Data are presented as mean ± SEM by one-way ANOVA (*n* = 3 per group), *P < 0.05. **C**–**H** METTL3 deficiency alleviates liver fibrosis, HA secretion, HAS2 expression and HSC activation. Data are presented as mean ± SEM by one-way ANOVA (*n* = 6 per group), *P < 0.05. Scale bar: 50 μm. **I**–**L** METTL3 deficiency promotes HSC senescence. Data are presented as mean ± SEM by one-way ANOVA (*n* = 3–6 per group), *P < 0.05. Scale bar: 50 μm.

Next, *METTL3*^*flox/flox*^ mice and adenovirus (Ad)-Des-CreERT2 combined with tamoxifen were used to construct HSC-specific METTL3-deficient mice, and a liver fibrosis model was induced. METTL3 levels were significantly reduced in both liver tissues and primary HSCs from Ad-Des-CreERT2 mice. Moreover, knockdown of METTL3 reversed the CCl_4_-induced m^6^A methylation levels (Fig. 7A, B). H&E, Sirius Red, and immunohistochemical staining revealed that liver injury was alleviated, collagen deposition was decreased, and METTL3- and α-SMA-positive signals were reduced after METTL3 deficiency in HSCs (Fig. 7C, D). The total HA content in liver tissues, serum samples, and primary cultured HSC supernatant was also reduced in the METTL3-deficient group (Fig. 7E). Immunofluorescence costaining revealed that METTL3 deficiency reduced HAS2 expression, decreased the proportion of activated HSCs, and increased the proportion of senescent HSCs (Fig. 7F–J). SA-β-Gal staining further confirmed that METTL3 deficiency promoted cellular senescence, and this conclusion was also supported by elevated CDKN2A, CDKN1A, and IL6 levels in mouse primary HSCs (Fig. 7K–L). These findings demonstrate that METTL3 is a key regulator of HA metabolism, HSC activation, and senescence during liver fibrosis. Deficiency of METTL3 in HSCs alleviates liver fibrosis.

### Targeted inhibition of MASP1 expression repairs the liver microenvironment to promote HSC senescence and alleviate liver fibrosis

**Fig. 8:**
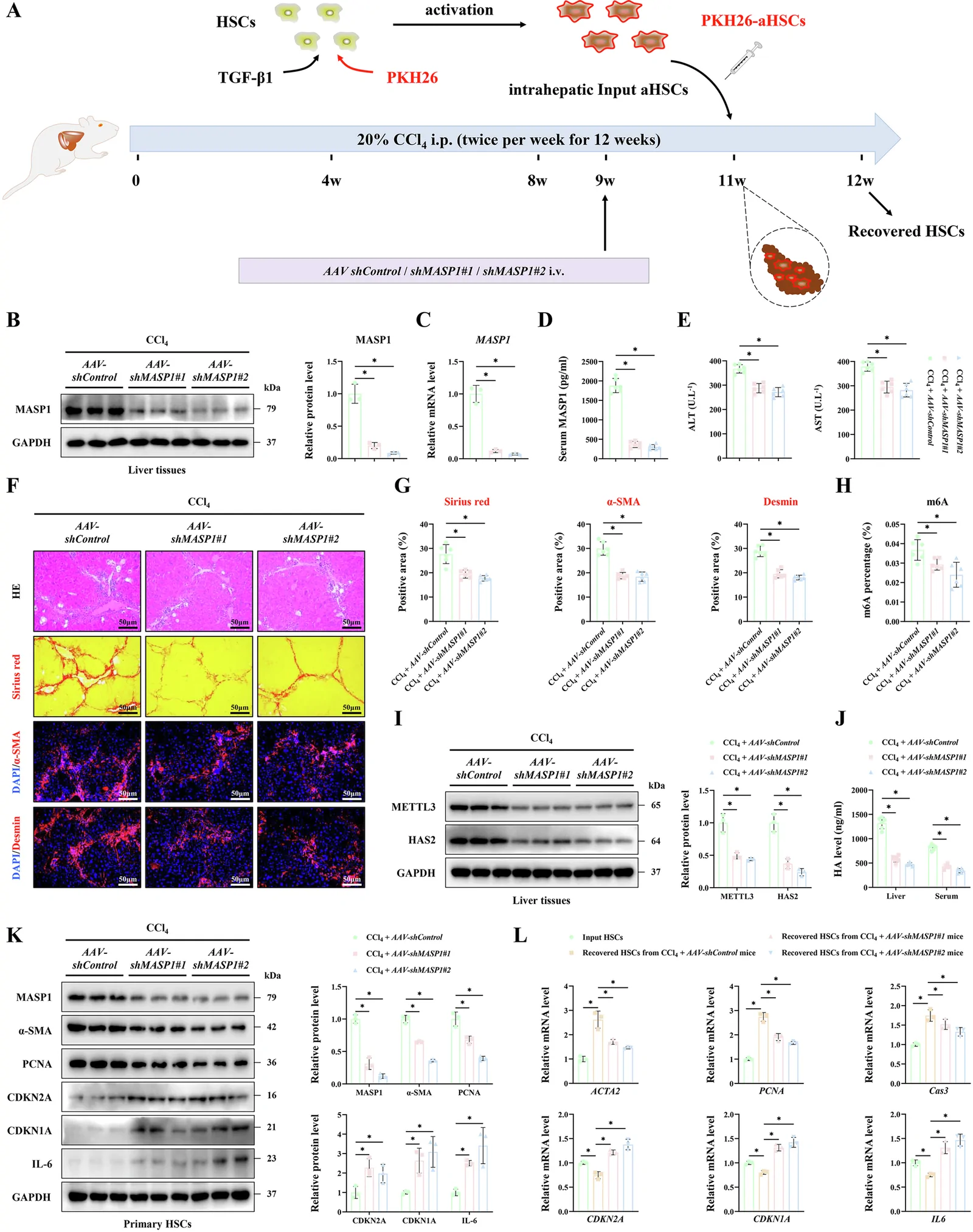
Inhibition of MASP1 promotes HSC senescence and alleviates liver fibrosis by modulating the fibrotic microenvironment. **A** Schematic diagram of the mouse modeling procedure. **B**–**D** Mice in the AAV-shMASP1 group exhibited reduced MASP1 expression. Data are presented as mean ± SEM by one-way ANOVA (*n* = 3–6 per group), *P < 0.05. **E**–**G** Knockdown of MASP1 alleviated liver fibrosis and injury. Data are presented as mean ± SEM by one-way ANOVA (*n* = 6 per group), *P < 0.05. Scale bar: 50 μm. **H** MASP1 knockdown decreased m^6^A methylation levels. Data are presented as mean ± SEM by one-way ANOVA (*n* = 6 per group), *P < 0.05. **I**–**J** MASP1 knockdown reduced HAS2 expression and HA level. Data are presented as mean ± SEM by one-way ANOVA (*n* = 3–6 per group), *P < 0.05. **K** MASP1 knockdown inhibited HSC activation and promoted HSC senescence. Data are presented as mean ± SEM by one-way ANOVA (*n* = 3 per group), *P < 0.05. **L** PKH26-labeled HSCs were also isolated from the indicated groups, compared to initial input HSCs, RT-qPCR showed that senescent HSCs were enhanced, while activated HSCs were inhibited when MASP1 was inhibited. Data are presented as mean ± SEM by one-way ANOVA (*n* = 3 per group), *P < 0.05.

Here, we speculate that the above process in which HA controls the inhibition of HSC senescence and enhances its activation is driven by MASP1 in the fibrotic microenvironment. Based on the sharply elevated MASP1 in the advanced liver fibrosis, to further define the role of MASP1, AAV targeting the liver for MASP1 knockdown (AAV-shMASP1) was administered at week 9 of the CCl₄-induced model. At week 11, PKH26-labelled activated-HSCs (aHSCs) were injected intrahepatically, and samples were collected one week later (Fig. 8A). The results revealed that mice treated with AAV-shMASP1 exhibited significantly reduced MASP1 protein and mRNA expression in the liver (Fig. 8B, C) and decreased serum MASP1 levels (Fig. 8D). Further assays revealed that liver injury was alleviated, fibrosis was reduced, and fewer activated HSCs were detected in the AAV-shMASP1 group (Fig. 8E–G), indicating that MASP1 inhibition ameliorated liver fibrosis. Both METTL3 expression and m^6^A levels were significantly downregulated following MASP1 knockdown (Fig. 8H–I). HAS2 expression and serum HA content were also reduced upon MASP1 knockdown (Fig. 8I, J). Subsequent analysis of mouse primary HSCs revealed that AAV-shMASP1 treatment reduced the degree of HSC activation and upregulated the expression of senescence-associated markers (Fig. 8K). More interestingly, compared with initial input HSCs, PKH26-labelled HSCs were also isolated from the indicated groups, and the RT-qPCR results suggested that the number of senescent HSCs was greater while the number of activated HSCs was lower when MASP1 expression was inhibited in the fibrotic microenvironment (Fig. 8L).

In summary, the liver fibrotic microenvironment determines the fate of HSCs. Our study demonstrated that HA-induced alterations in the microenvironment exacerbate liver fibrosis. LMW-HA and HMW-HA promote HSC activation and inhibit HSC senescence through the CD44-p38-ATF2 and TLR4-SerpinE1 axes, respectively. Furthermore, MASP1 upregulates HAS2 expression and promotes HMW-HA synthesis by regulating METTL3-mediated m^6^A methylation, and HYAL2 mediates the conversion of HMW-HA to LMW-HA. Therefore, targeting the MASP1-METTL3-HAS2 axis to disturb the HA-controlled fibrotic microenvironment may represent a promising therapeutic strategy for scavenging activated hepatic stellate cells and reversing liver fibrosis.

## Discussion

Liver fibrosis is a pivotal and reversible pathological stage during the progression of chronic liver diseases, with HSCs serving as central executors of extracellular matrix deposition and fibrosis modulation [21]. This study systematically delineates a previously underappreciated mechanism through which HA, a major component of the fibrotic extracellular matrix, orchestrates both HSC activation and senescence in a molecular-weight-dependent manner, thereby driving the persistence of advanced liver fibrosis. Our findings reveal the different states of HSCs in different fibrotic microenvironments and highlight the dual regulatory role of HA in modulating HSC fate. On the one hand, LMW-HA promotes HSC activation via the CD44-p38-ATF2 signalling axis, enhancing their proliferative and fibrogenic capacities. On the other hand, HMW-HA suppresses HSC senescence primarily through the TLR4-SerpinE1 pathway, thereby inhibiting a key cellular process associated with fibrosis resolution. This differential signalling not only clarifies the paradox of the context-dependent role of HA but also highlights how HA matrix remodelling in advanced fibrosis creates a microenvironment that favours fibrogenesis over regression.

Numerous clinical studies have demonstrated that serum HA levels are strongly correlated with the severity of liver fibrosis across various aetiologies, including nonalcoholic fatty liver disease (NAFLD), chronic hepatitis C, alcoholic liver disease, and primary biliary cholangitis [22–24]. Specifically, serum HA has been shown to accurately discriminate between absent/minimal fibrosis and intermediate/severe fibrosis, with areas under the ROC curve exceeding 0.85 for severe fibrosis and cirrhosis [22]. Moreover, elevated HA levels independently predict liver-related mortality and transplant-free survival in patients with chronic liver disease. These clinical observations firmly establish HA as a reliable noninvasive biomarker of fibrosis progression [25]. Consistent with this, studies in other systems have shown that HMW-HA possesses anti-senescence properties, whereas LMW-HA is proinflammatory and can promote cellular activation [24, 26]. Furthermore, in human amniotic epithelial stem cells (hAECs), HA delays cellular senescence by activating the AKT/mTOR signalling pathway, reducing the expression of senescence markers, altering the cell cycle, and significantly enhancing the differentiation capacity of hAECs [27]. In the context of skin ageing, compared with young and nonsenescent fibroblasts, senescent fibroblasts secrete significantly lower amounts of HA. The overexpression of miR-23a-3p leads to reduced HAS2-mediated HA synthesis, increased expression of senescence-associated markers, and decreased cell proliferation. The HA microenvironment thus constitutes an effector mechanism of dermal ageing and cellular senescence [28]. Thus, our mechanistic understanding of the role of HA in regulating HSC senescence and activation not only aligns with the long-standing clinical observation that HA levels mirror fibrosis severity but also provides a causal biological explanation for why elevated HA levels are associated with poor outcomes in patients with liver fibrosis. Our present study provides a mechanistic framework that directly supports and explains these clinical findings. We demonstrate that in advanced liver fibrosis, HA is not merely a passive byproduct of matrix remodelling but an active regulator of HSC fate. Specifically, we show that HMW-HA inhibits HSC senescence via the TLR4-SerpinE1 axis, thereby sustaining HSC activation and promoting disease progression, while LMW-HA promotes HSC activation through the CD44-p38-ATF2 pathway. This dual, molecular-weight-dependent function of HA offers a mechanistic explanation for the clinical correlation between increasing HA levels and worsening fibrosis. As fibrosis progresses, the accumulation of HMW-HA suppresses HSC senescence, allowing continued fibrogenesis.

Multiple independent studies have demonstrated that 4-MU has potent antifibrotic effects on various preclinical models of liver fibrosis. In a CCl₄-induced mouse model, 4-MU treatment significantly reduced collagen and hyaluronan deposition, suppressed HSC transdifferentiation to myofibroblasts, and downregulated the profibrotic cytokine FSTL1 [29]. In a choline-deficient L-amino acid-defined (CDAA) diet-induced NASH model, 4-MU administration ameliorated hepatic steatosis, hepatocyte injury, inflammation, and fibrogenesis, accompanied by reduced expression of Col1a1, Acta2, and Notch1 [30]. Furthermore, 4-MU has been shown to inhibit hyaluronan synthesis by depleting UDP-glucuronic acid and downregulating HAS2 expression, thereby reducing the production of both high-molecular-weight and low-molecular-weight HA [31]. Notably, while 4-MU at a very high dose is required to achieve significant antifibrotic effects in mice, its efficacy in reducing liver fibrosis in animal models is comparable to or even better than that of Resmetirom, and it has already been approved for use in several countries, with excellent safety profiles [32]. This favourable safety record, together with its demonstrated efficacy in multiple animal models of liver fibrosis, positions 4-MU as a promising candidate for drug repurposing in fibrotic liver diseases.

Homeostasis of the extracellular matrix is closely associated with disease onset and progression. Under physiological conditions, the extracellular matrix primarily maintains tissue function and integrity and regulates cell differentiation [33–35]. In pathological states such as vascular calcification, osteoarthritis, cancer, and fibrosis, the extracellular matrix becomes markedly dysregulated and promotes disease progression [36–38]. Cells within the microenvironment are continuously regulated by the extracellular matrix, primarily through cell surface receptors such as proteoglycans (PGs), integrins, and HA receptors, including CD44 [39]. These interactions subsequently influence cellular function and behaviour [40]. In our study, CD44 and TLR4 on the surface of HSCs were identified as key receptors mediating HA-dependent regulation and impacting HSC differentiation. CD44 is typically linked to HMW-HA binding, and TLR signalling is more often associated with fragmented or low-molecular-weight HA. However, our results indicated that LMW-HA activated HSCs mainly via CD44-mediated signalling and that HMW-HA inhibited HSC senescence mainly via TLR4-mediated signalling during advanced liver fibrosis. CD44 knockdown reversed LMW-HA-induced HSC activation, whereas TLR4 knockdown did not affect this activity. Additionally, TLR4 knockdown abolished the anti-senescence effect of HMW-HA, whereas knockdown of CD44 failed to reverse this effect. Mechanistically, LMW-HA primarily binds to CD44, while HMW-HA primarily binds to TLR4 in activated-HSCs. As reported, in normal human epidermal keratinocytes (NHEKs), LMW-HA significantly upregulates CD44 protein expression, whereas medium- and high-molecular-weight HA do not exert this effect [41]. In a study by Vistejnova et al., LMW-HA mediated CD44-dependent chemokine induction and enhanced the innate immune response in human dermal fibroblasts [42]. Consistent with these findings, CD44 also participated in LMW-HA-mediated HSC activation in our study. Although some studies have reported opposite conclusions, differences in cell types and disease conditions may account for these discrepancies. Taken together, our experimental results confirm that LMW-HA promotes HSC activation through CD44-mediated signalling pathways, whereas HMW-HA inhibits HSC senescence via TLR4 during liver fibrosis.

METTL3-mediated m6A methylation has been implicated in the progression of various diseases. For instance, METTL3 is upregulated in M1-polarized mouse macrophages and promotes inflammatory responses [43]; METTL3-dependent m6A modification suppresses SOCS2 mRNA expression, thereby accelerating tumorigenesis [44]; and METTL3 enhances fatty acid metabolism by increasing the m6A methylation of FASN, leading to impaired hepatic insulin sensitivity [45]. Our study revealed that METTL3 promotes m6A methylation in HSCs, which in turn increases HAS2 mRNA expression and HA synthesis and secretion. Moreover, our results confirm that during the progression of liver fibrosis, HAS2 is responsible for the generation of HMW-HA, while HYAL2 is responsible for hydrolysing HMW-HA into LMW-HA, thereby exacerbating liver fibrosis by promoting the activation and inhibiting the senescence of HSCs. These findings provide a novel perspective on how METTL3 contributes to the pathogenesis of liver fibrosis. Given that METTL3 has regulatory functions in multiple cell types and that liver fibrosis involves intricate intercellular crosstalk, future studies should expand this investigation to other cell populations to explore whether METTL3 also promotes fibrogenesis in these contexts.

Previous studies have identified MASP1 as a potential predictor of the severity of liver fibrosis [46, 47]. Work from our team has also demonstrated that hepatocyte-derived MASP1 promotes HSC activation [14]. In the present study, we further revealed that MASP1 enhances HA metabolism through METTL3-mediated m^6^A methylation, thereby reshaping the fibrotic microenvironment in advanced stages and suppressing HSC senescence. These findings expand the functional repertoire of MASP1 in advanced liver fibrosis and elucidate how hepatocyte-HSC crosstalk critically modulates the fibrotic niche, sustaining fibrosis progression. We note that the roles of MASP1 and METTL3 in m6A methylation and complement activation may vary across tissues and disease phases. In our study, in the context of advanced liver fibrosis, MASP1 is highly expressed, and the MASP1-METTL3 axis drives HSC activation and fibrosis progression, thereby representing a promising therapeutic target. However, systemic inhibition of this axis could interfere with normal complement-mediated immune surveillance or RNA methylation homeostasis in other organs. Furthermore, given that MASP1 is involved in the complement system, future therapeutic strategies targeting MASP1 for liver fibrosis should carefully assess the potential risk of immune impairment caused by excessive MASP1 expression. The development of therapeutic agents that specifically target MASP1 in hepatic stellate cells rather than in other cell types may help overcome this issue.

In summary, our study highlights the pivotal role of HA in remodelling the microenvironment of advanced liver fibrosis. We demonstrate that LMW-HA and HMW-HA promote HSC activation and suppress HSC senescence, respectively, and that the MASP1-METTL3-HAS2 axis drives HA metabolism to hinder the clearance of active HSCs through senescence, leading to their sustained activation. Therefore, targeting HA synthesis or its degradation pathways to repair the fibrotic microenvironment may represent a promising therapeutic strategy for liver fibrosis in the future.

## Supplementary information

Supplementary Information

Supplementary Information

## Data Availability

All the raw data are available from the corresponding author upon reasonable request.
