# Supplementary material for: MASP1‑METTL3‑HAS2 axis aggravates liver fibrosis through HMW‑HA‑mediated senescence inhibition and LMW‑HA‑induced activation of hepatic stellate cells
Source: Cell Death Dis. 2026 Aug 13;17(1):958. doi: 10.1038/s41419-026-09158-2 (PMC13752318; doi:10.1038/s41419-026-09158-2)
Supplement: Supplementary file 1 [file 41419_2026_9158_MOESM2_ESM.pdf]

1C

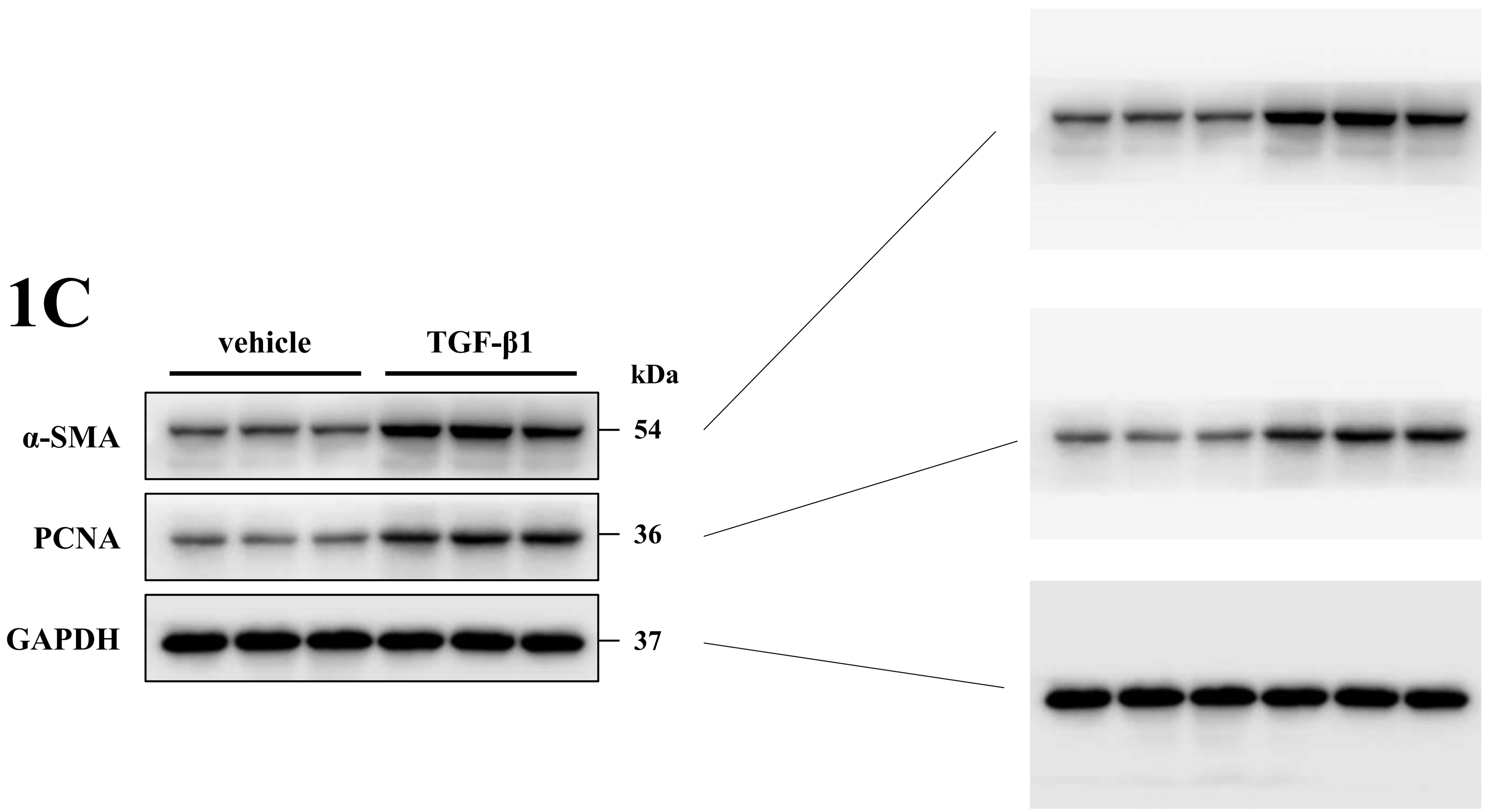
vehicle
TGF-β1
kDa
α-SMA
54
PCNA
36
GAPDH
37

2F

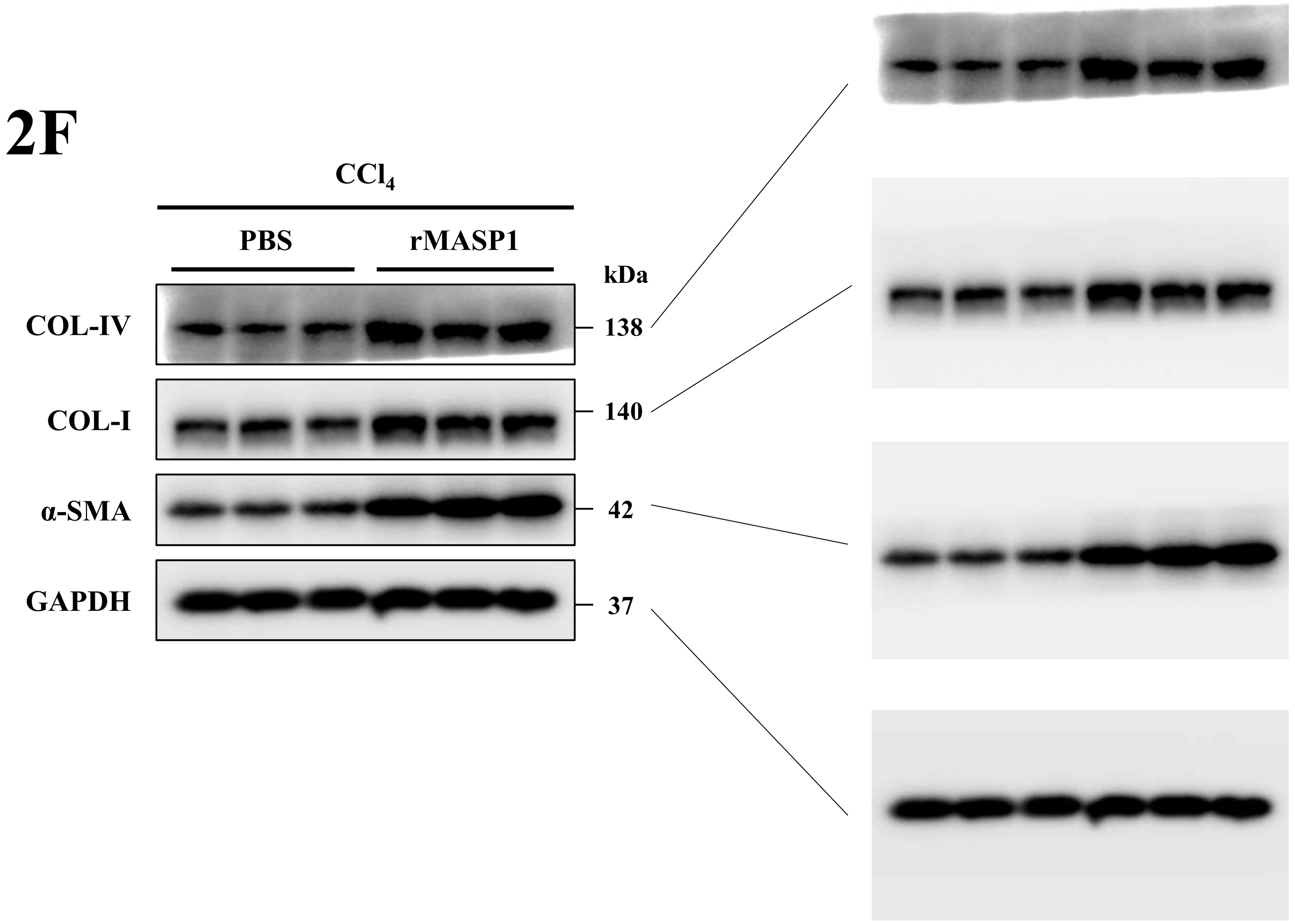
CCl4
PBS
rMASP1
kDa
COL-IV
138
COL-I
140
α-SMA
42
GAPDH
37

2G

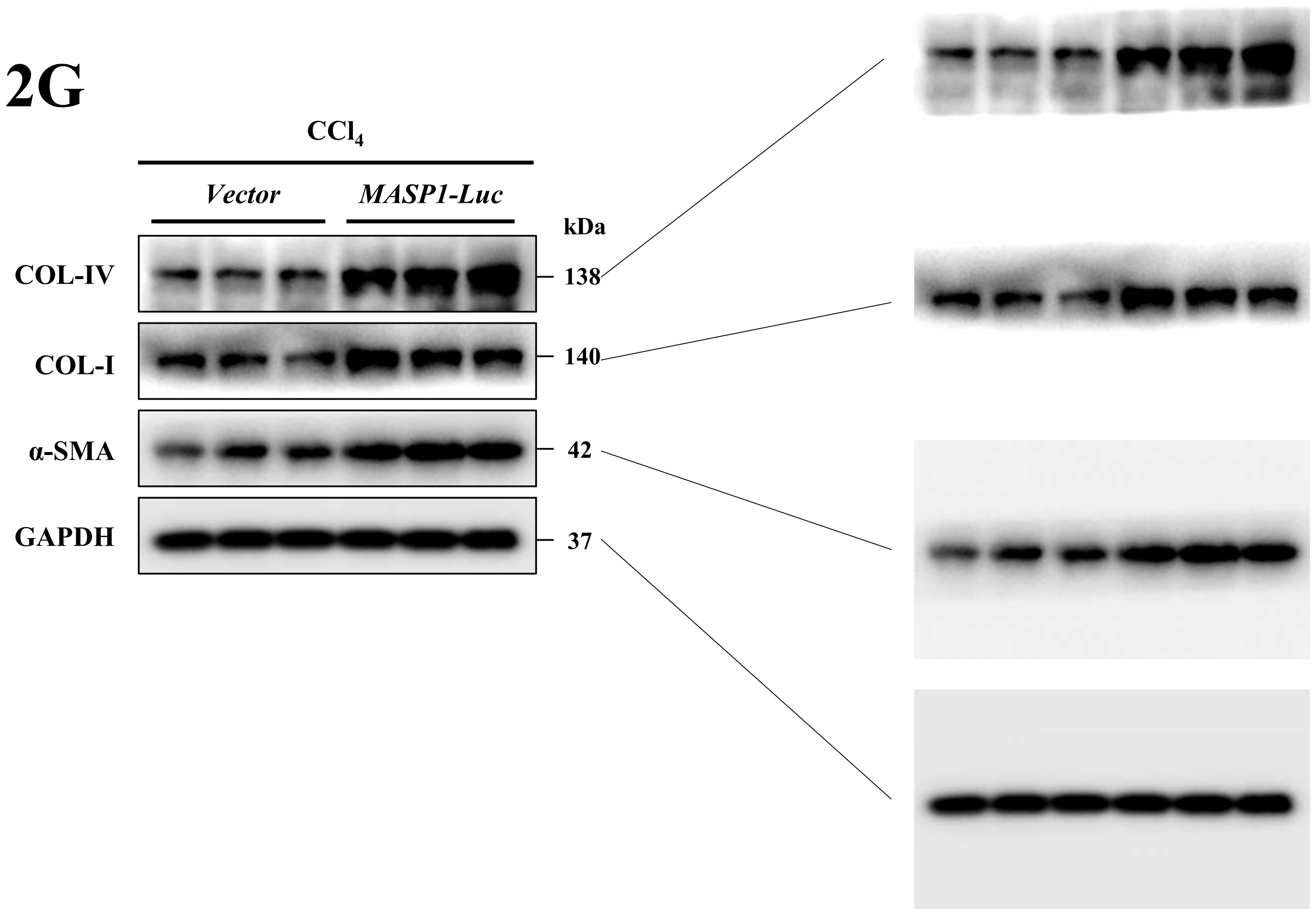
CCl4
Vector
MASP1-Luc
kDa
COL-IV
138
COL-I
140
α-SMA
42
GAPDH
37

2I Left

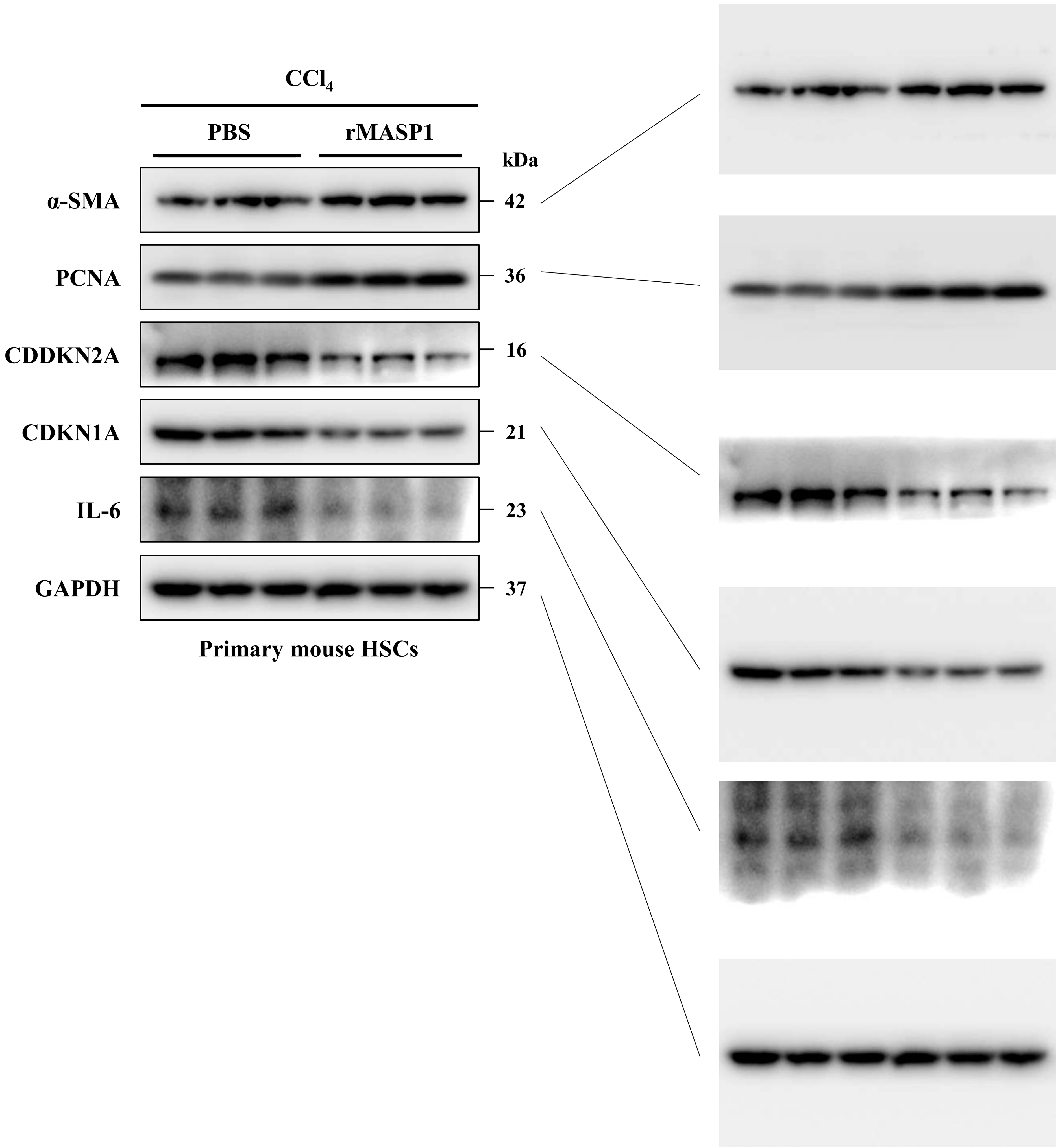

2I Right

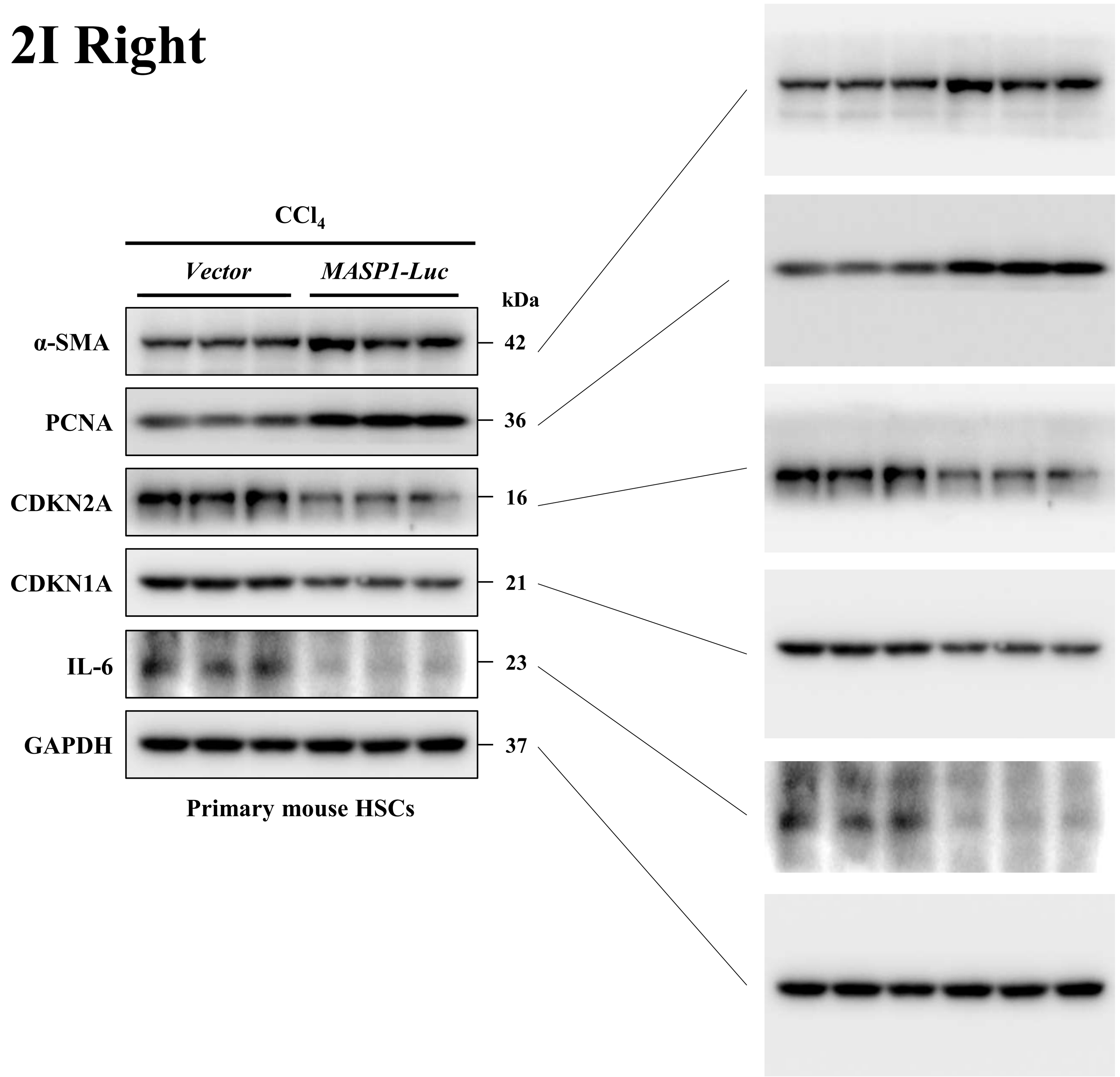

3E

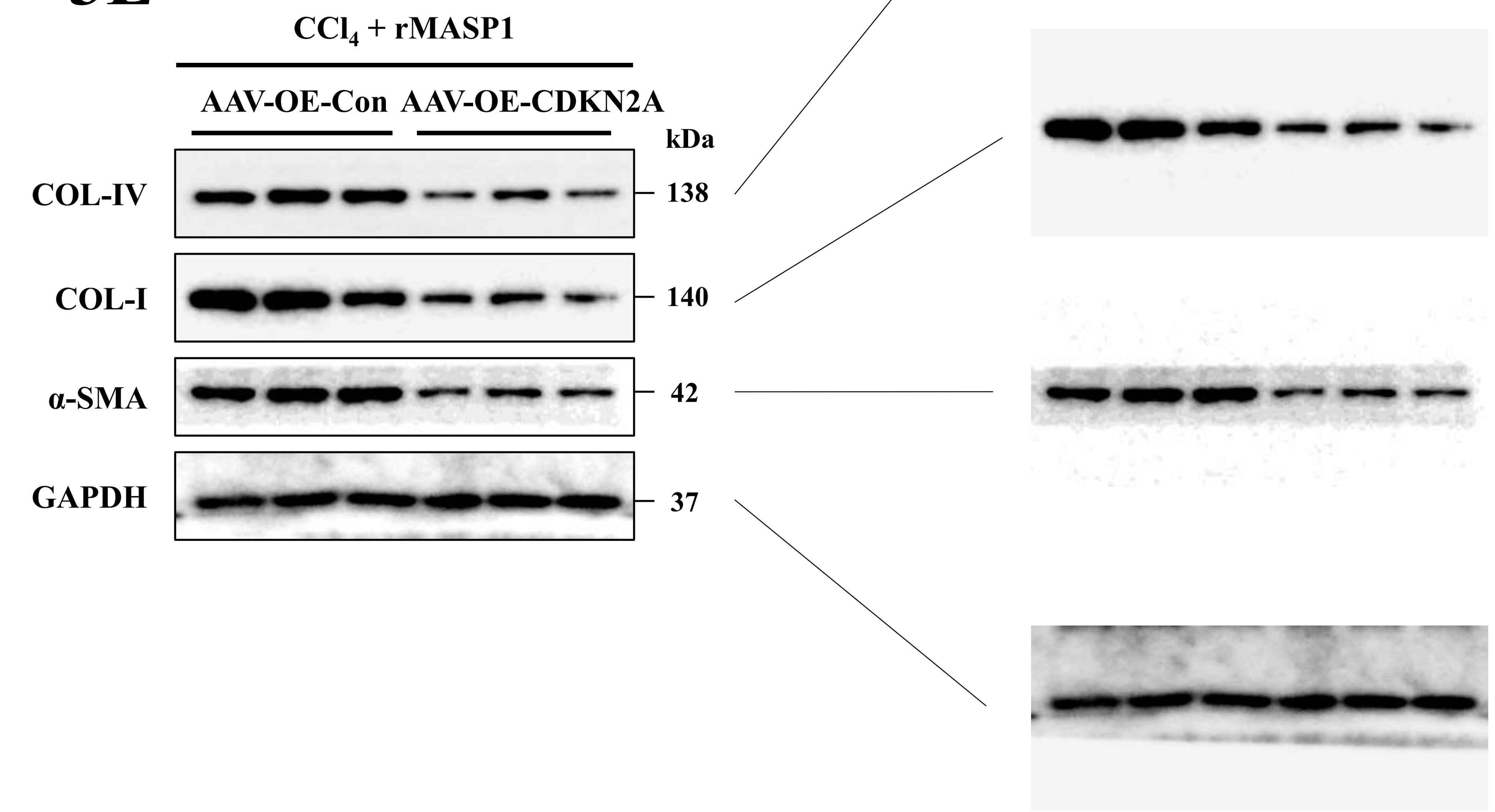

3F

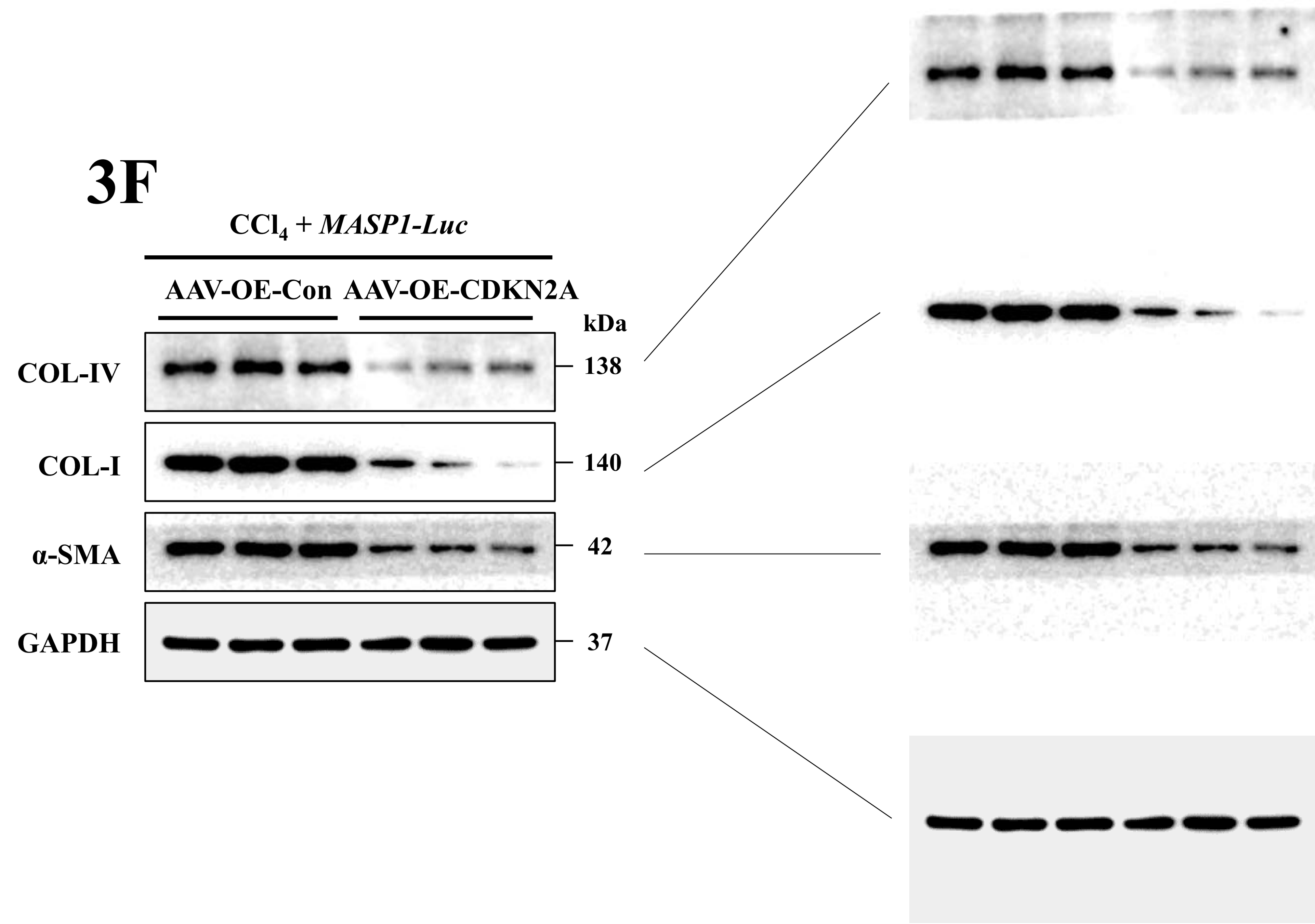

3H Left

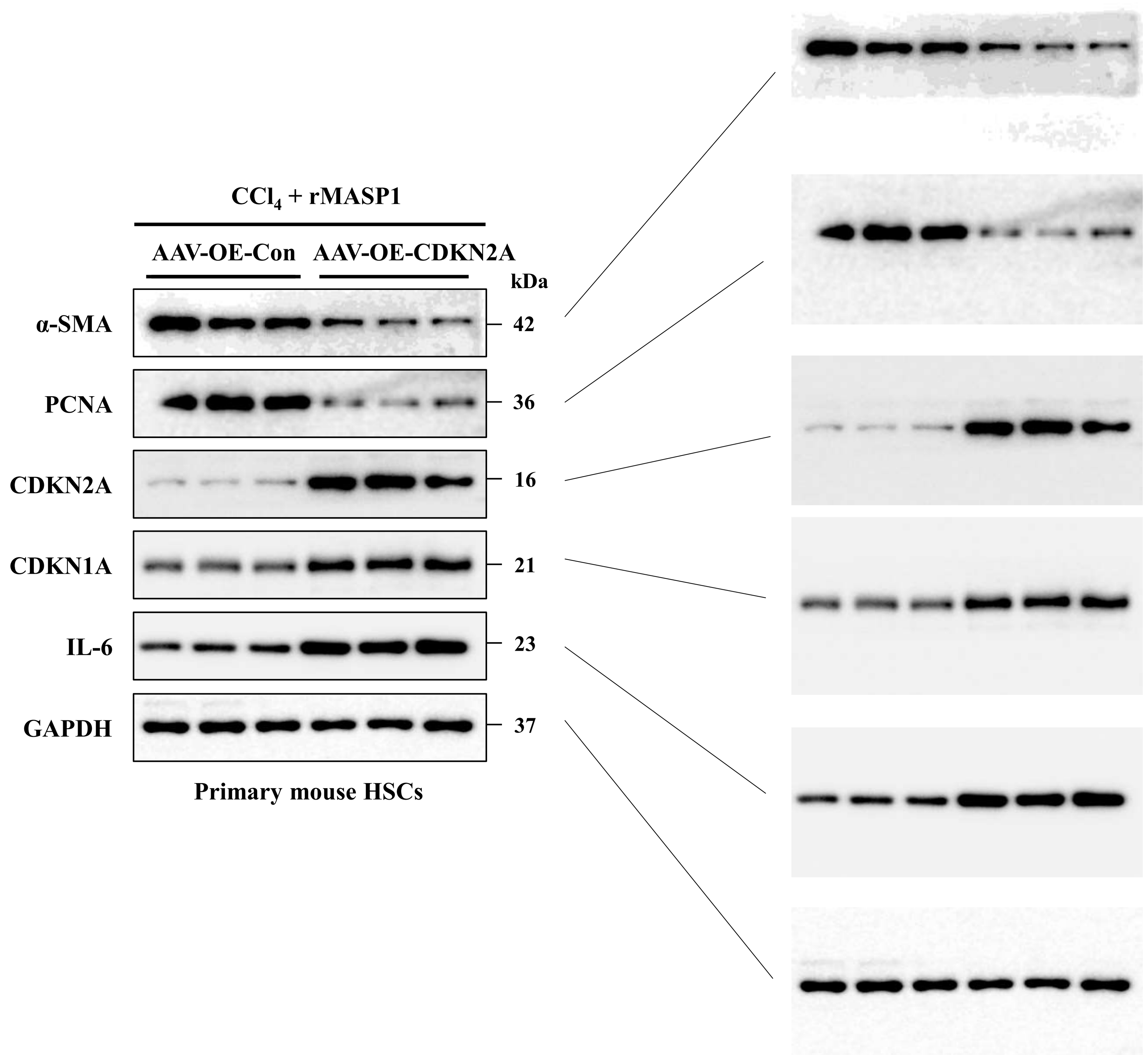

3H Right

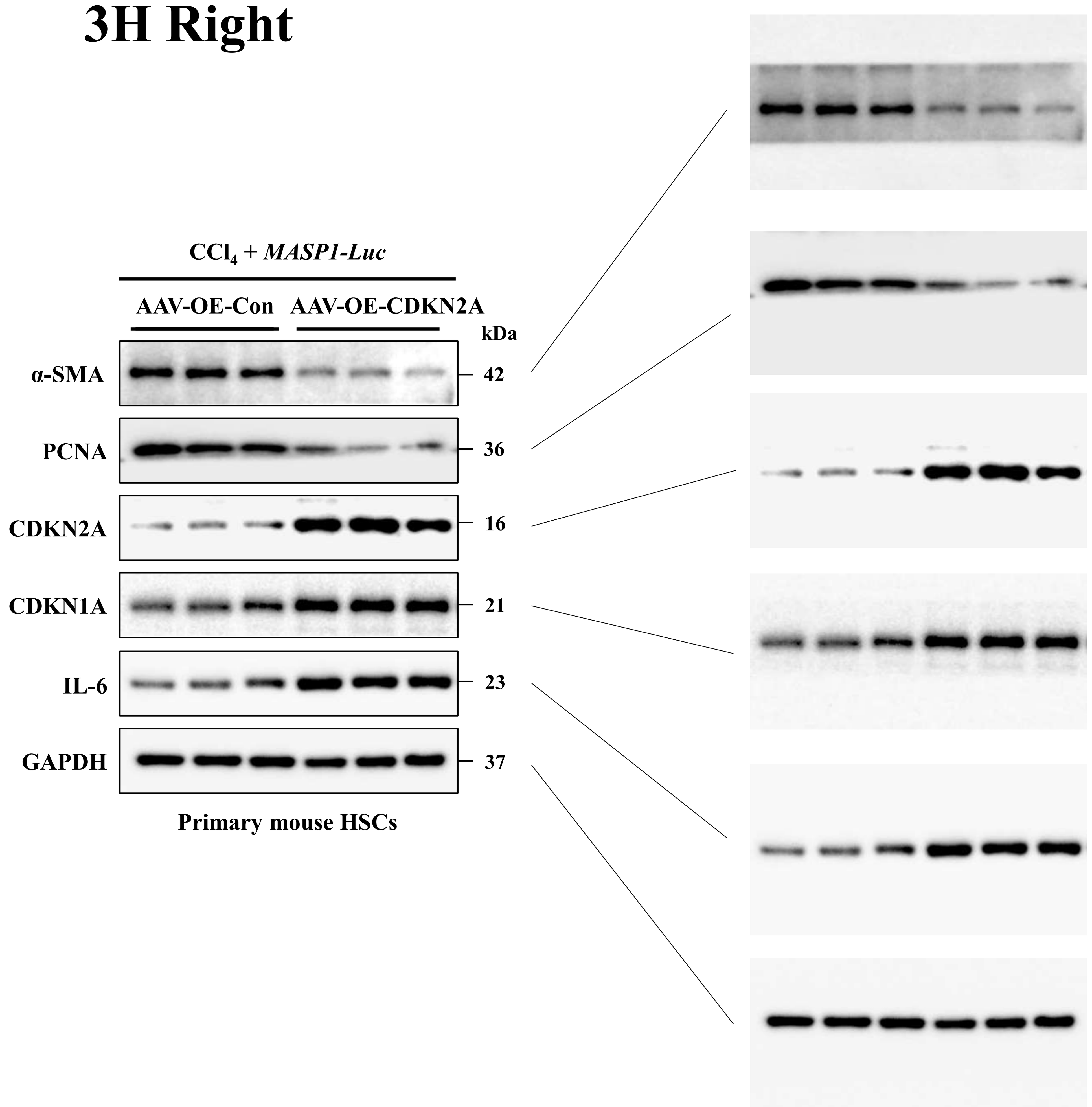

4F

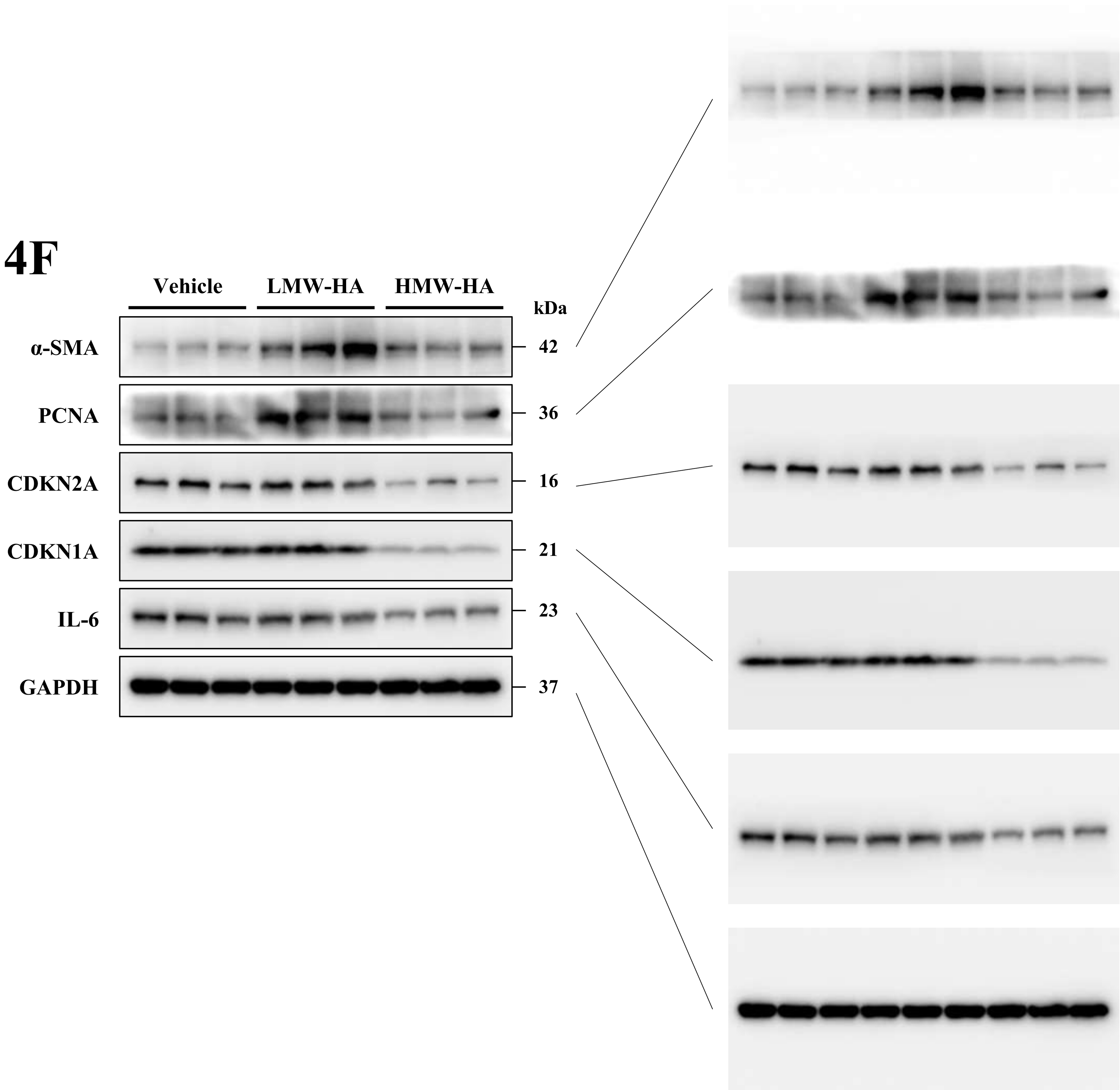

Vehicle
LMW-HA
HMW-HA
kDa
α-SMA
42
PCNA
36
CDKN2A
16
CDKN1A
21
IL-6
23
GAPDH
37

4G

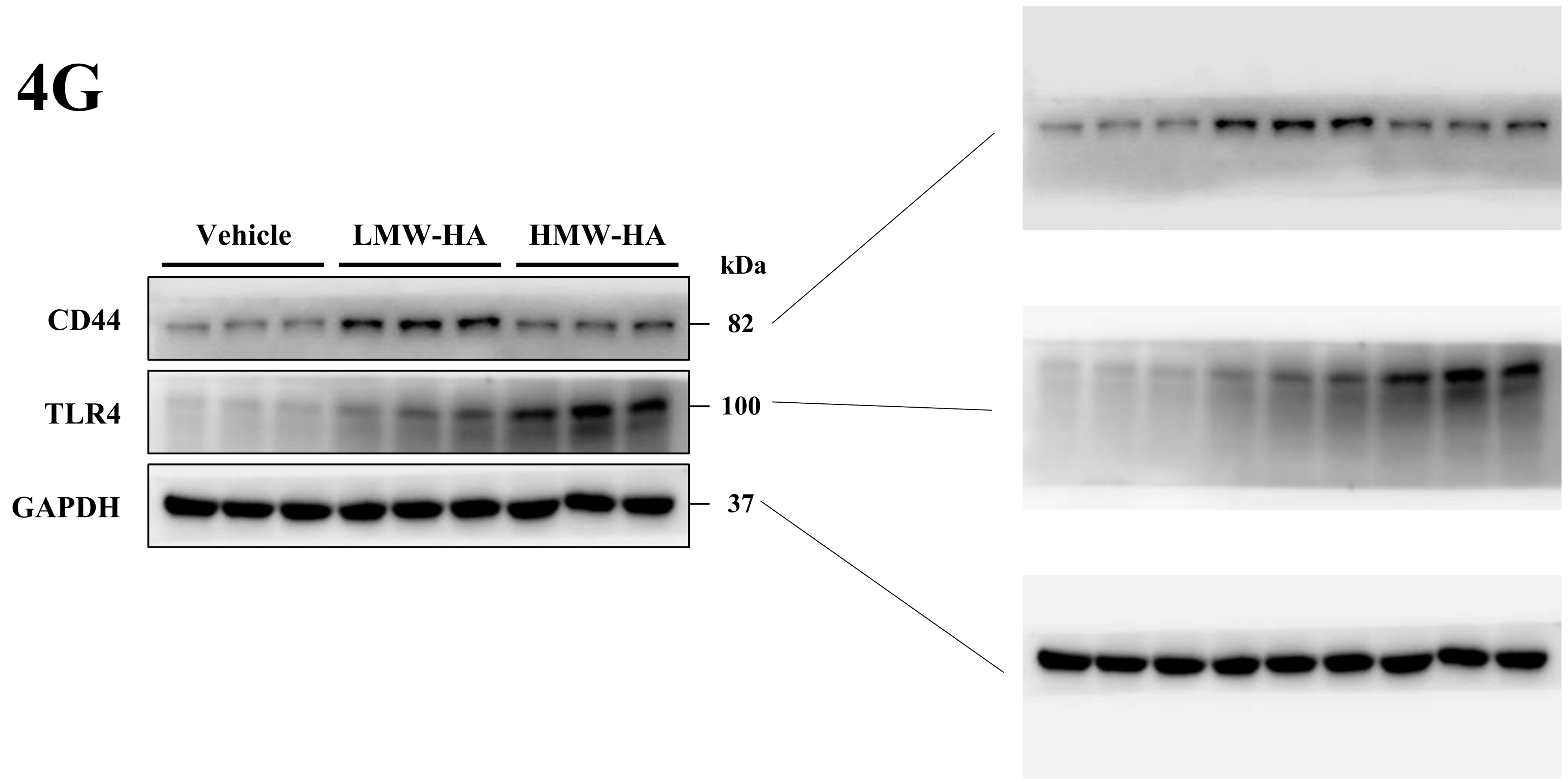

Vehicle
LMW-HA
HMW-HA
kDa
CD44
82
TLR4
100
GAPDH
37

4I

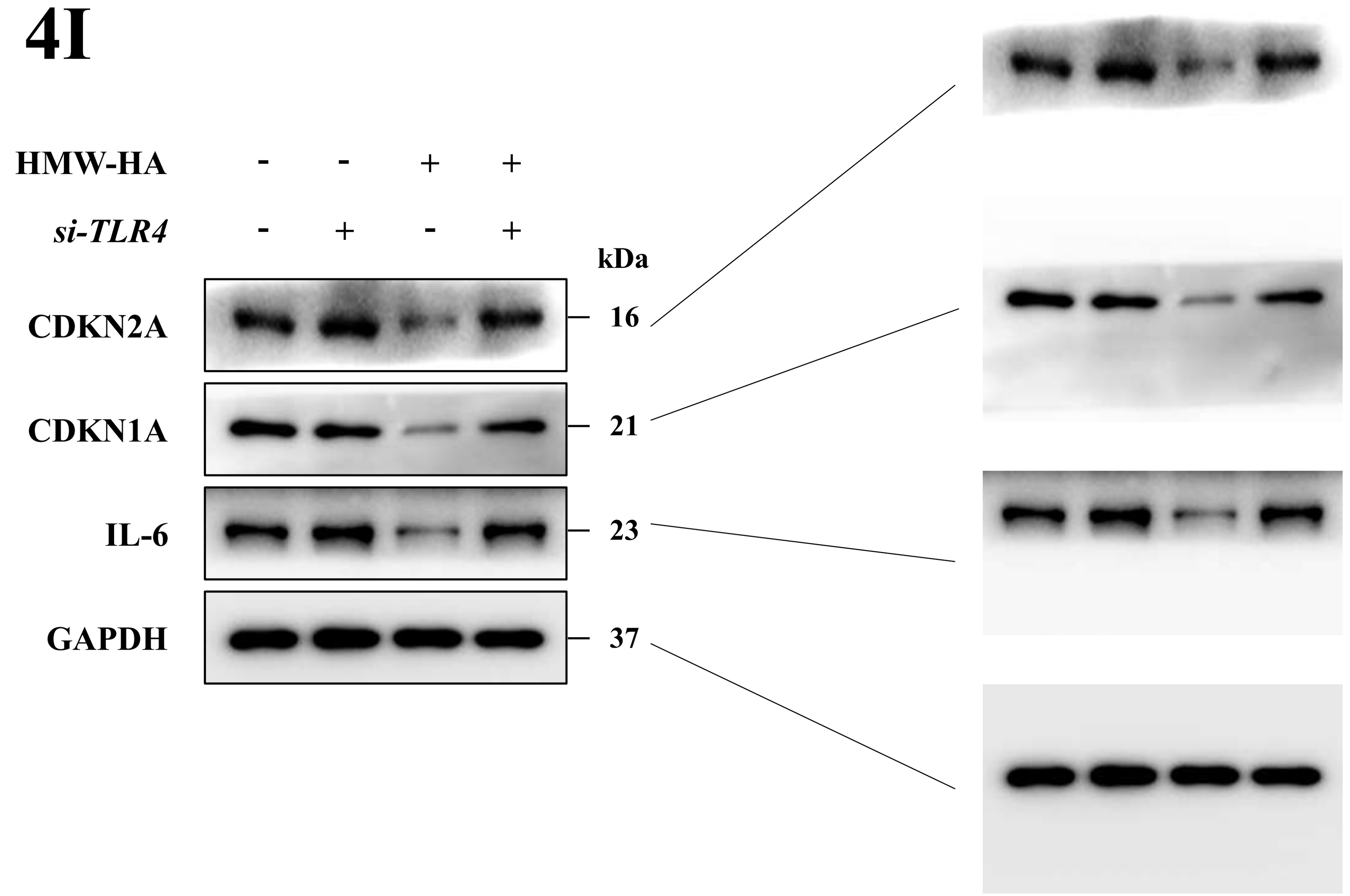

HMW-HA - - + +
si-TLR4 - + - +
kDa
CDKN2A
16
CDKN1A
21
IL-6
23
GAPDH
37

5E

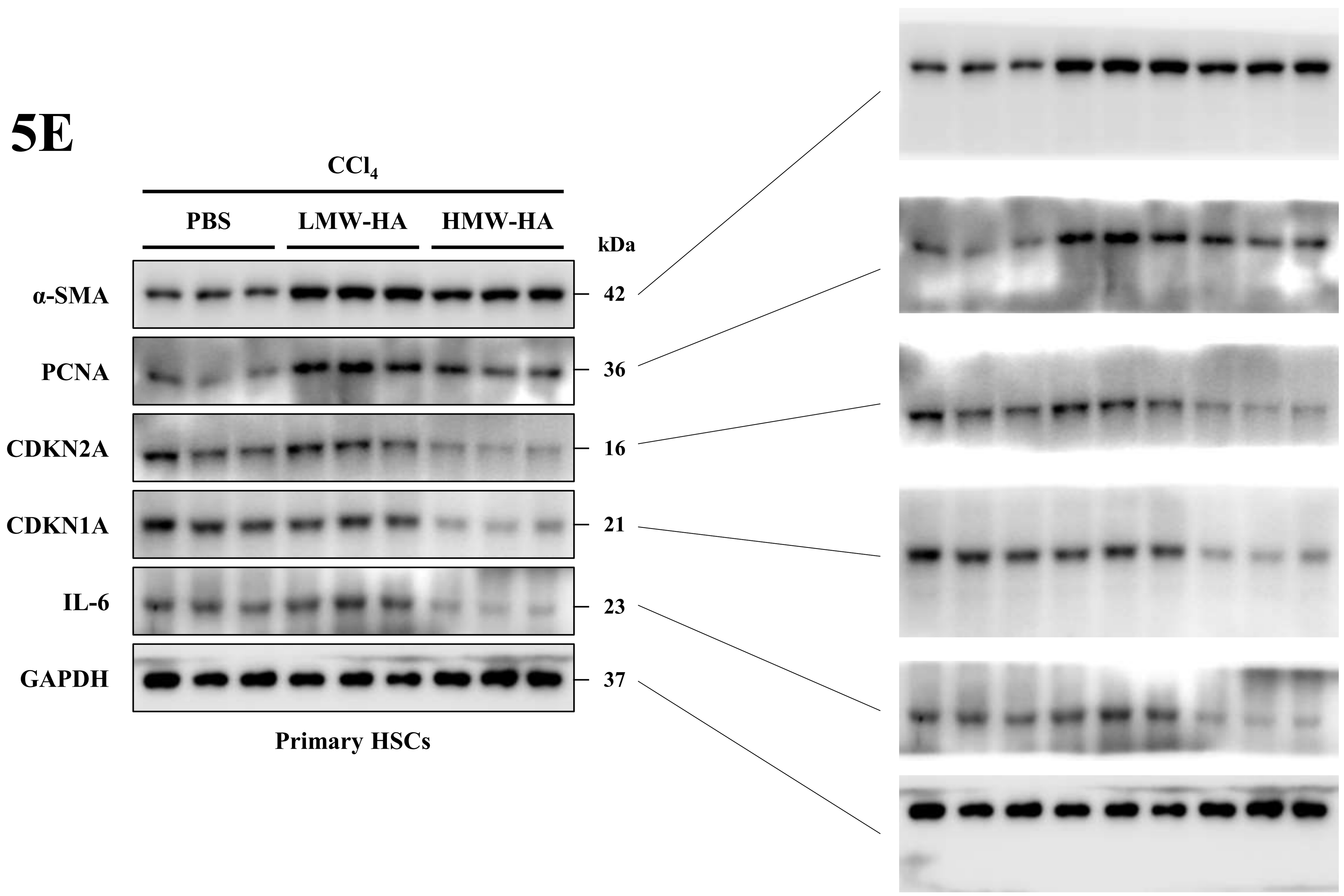

CCl4
PBS
LMW-HA
HMW-HA
kDa
α-SMA
42
PCNA
36
CDKN2A
16
CDKN1A
21
IL-6
23
GAPDH
37
Primary HSCs

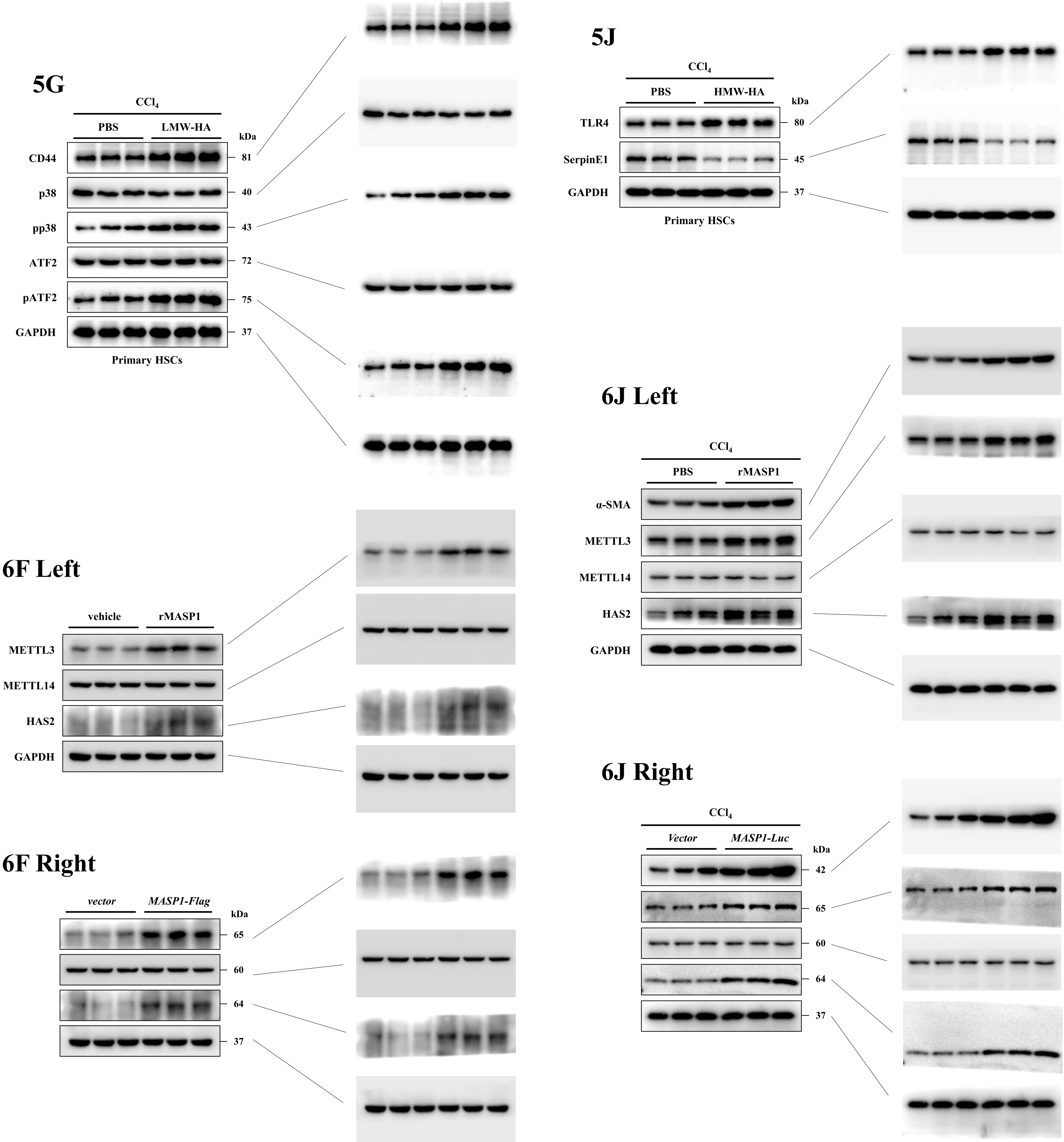

5G
CCl4
PBS
LMW-HA
kDa
CD44
p38
pp38
ATF2
pATF2
GAPDH
81
40
43
72
75
37
Primary HSCs
5J
CCl4
PBS
HMW-HA
kDa
TLR4
SerpinE1
GAPDH
80
45
37
Primary HSCs
6F Left
vehicle
rMASP1
METTL3
METTL14
HAS2
GAPDH
6F Right
vector
MASP1-Flag
kDa
65
60
64
37
6J Left
CCl4
PBS
rMASP1
α-SMA
METTL3
METTL14
HAS2
GAPDH
6J Right
CCl4
Vector
MASP1-Luc
kDa
42
65
60
64
37

6O Left

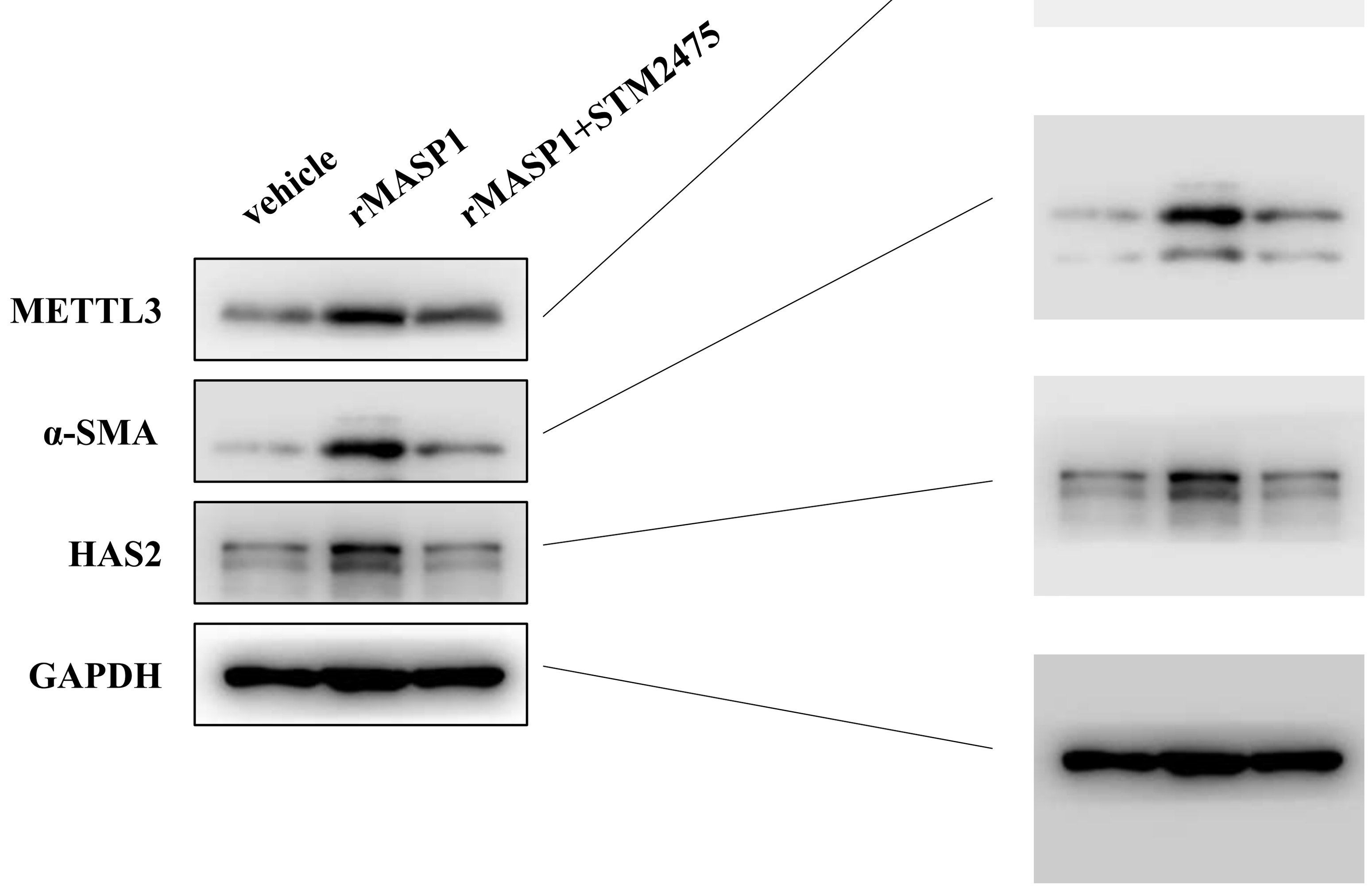

8B

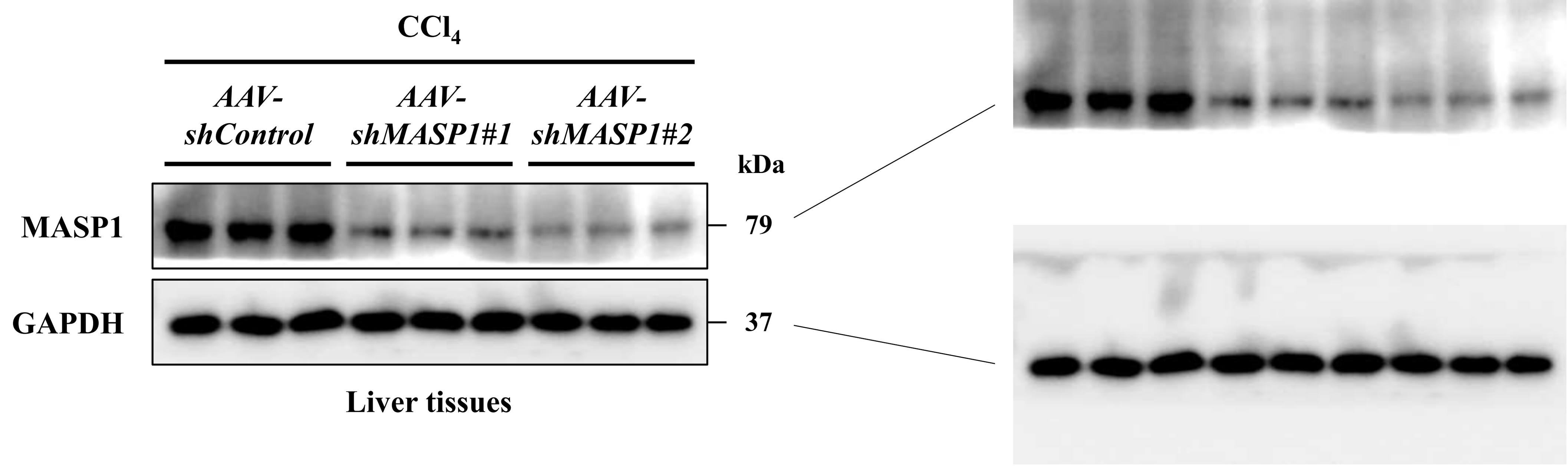

6O Right

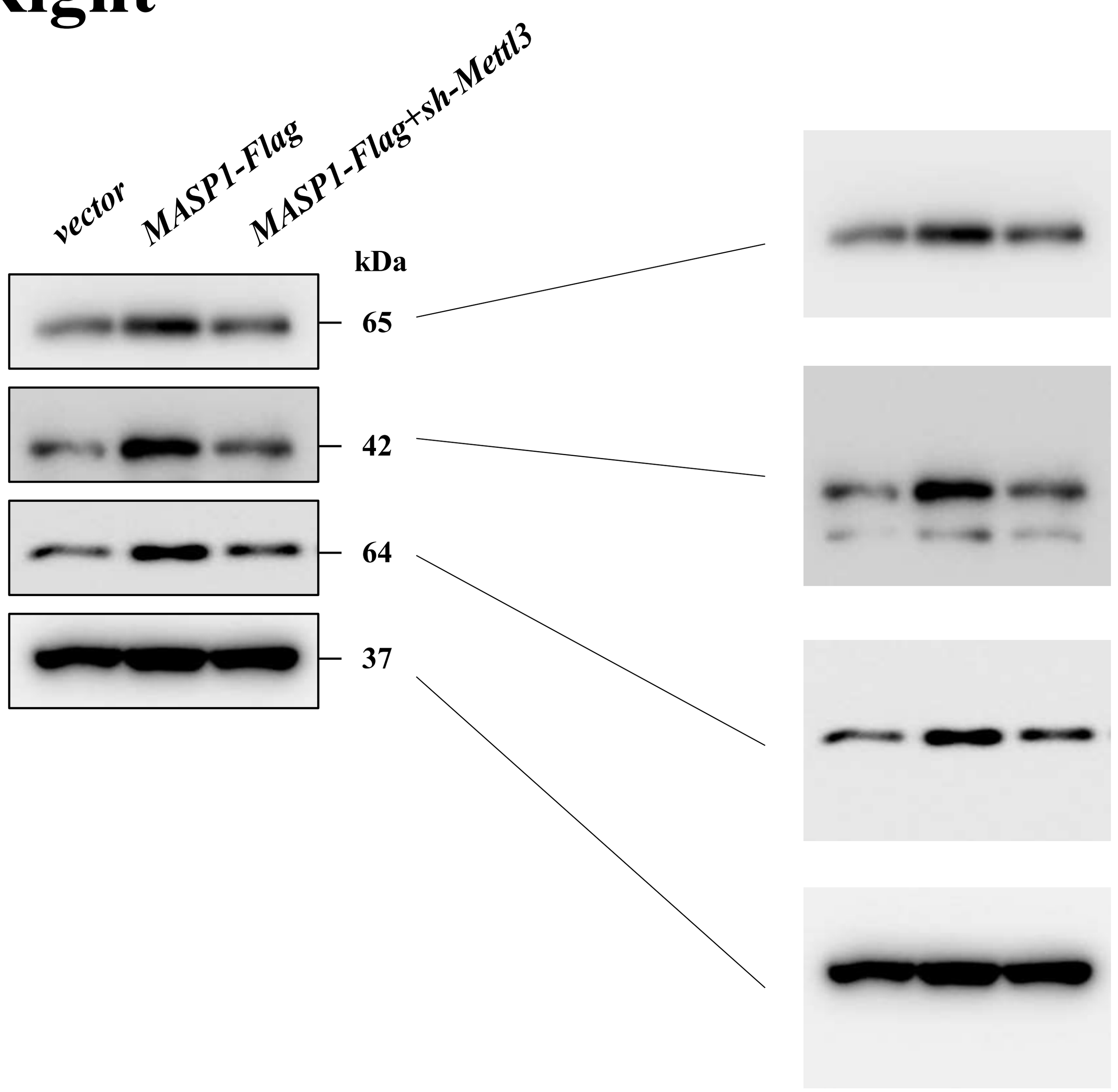

8I

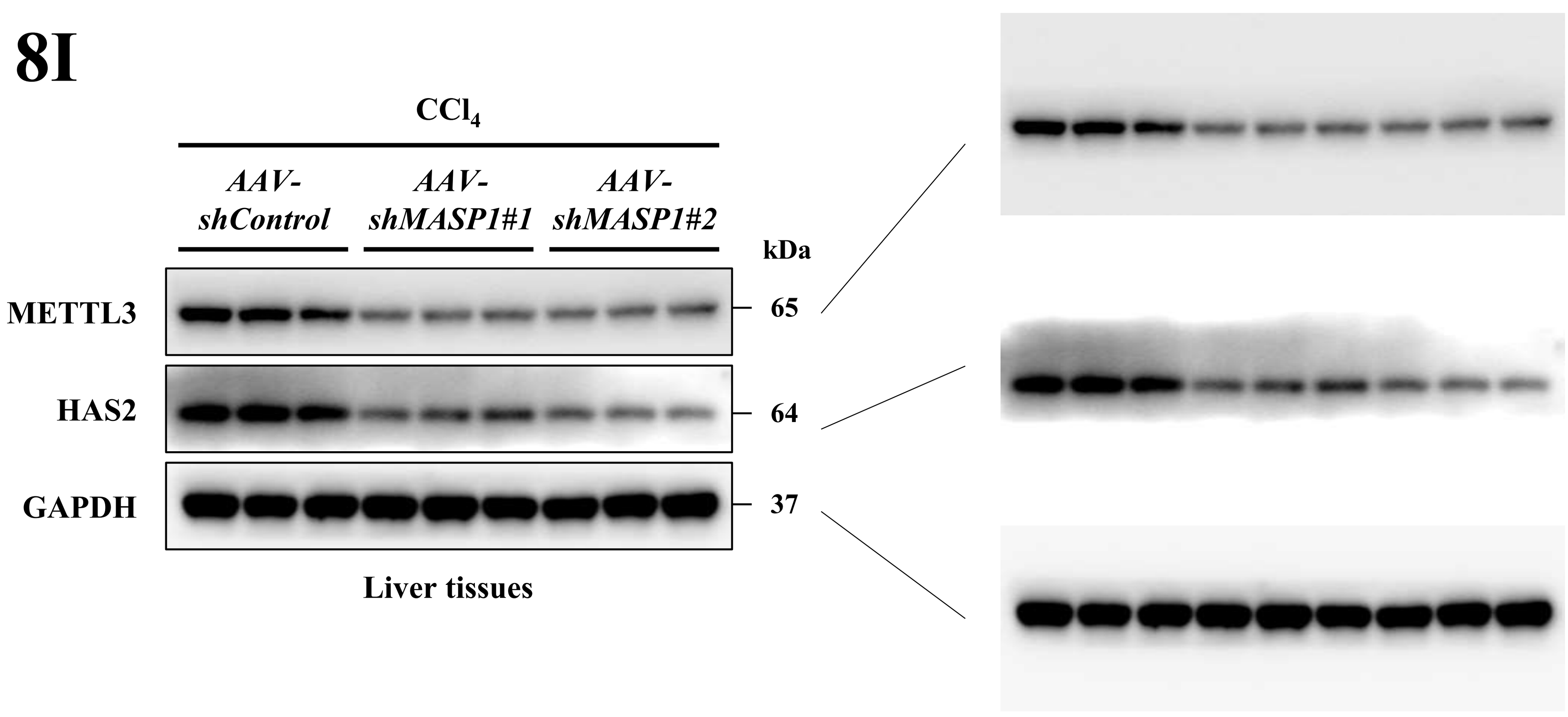

8K

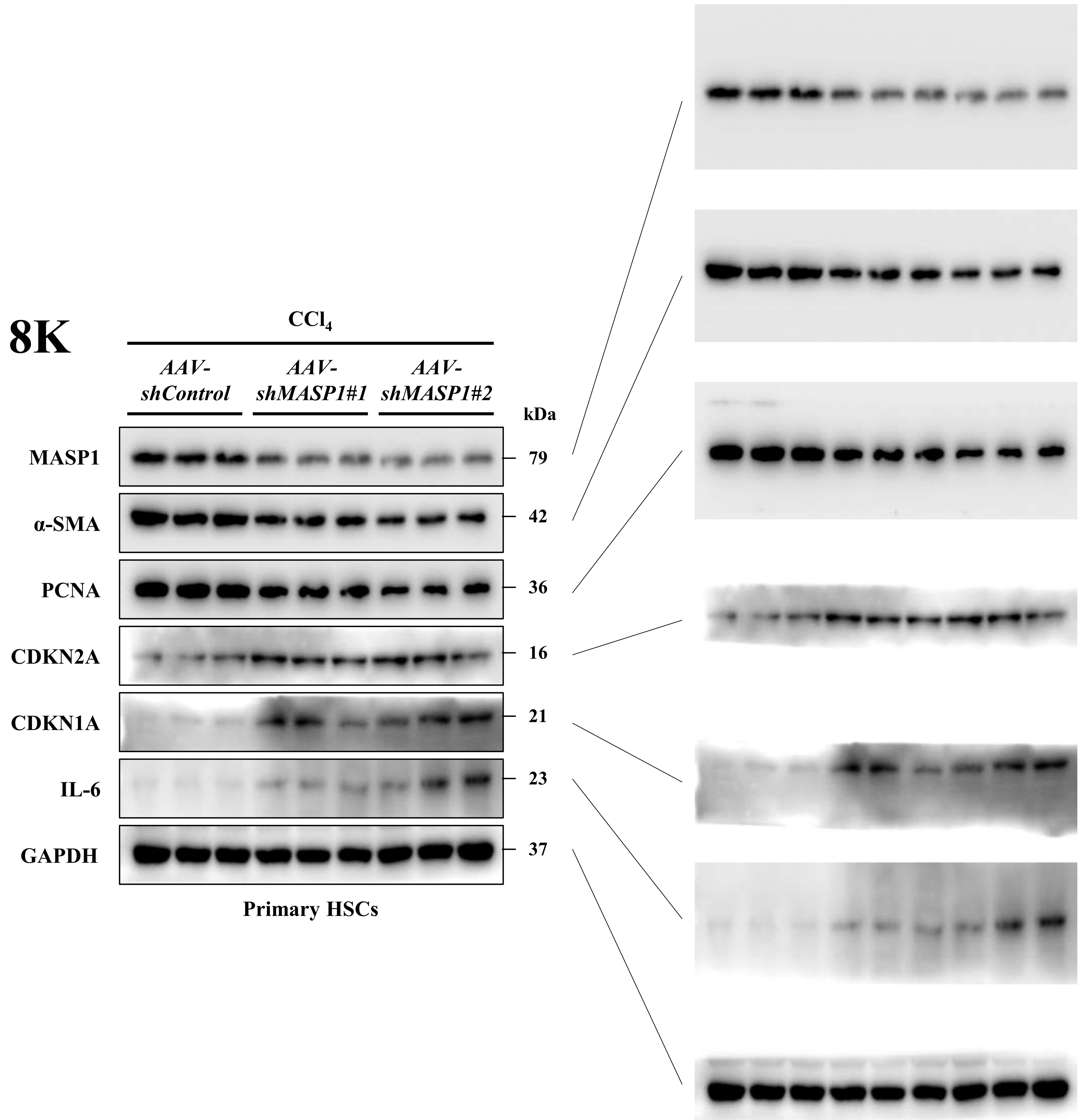

S2A

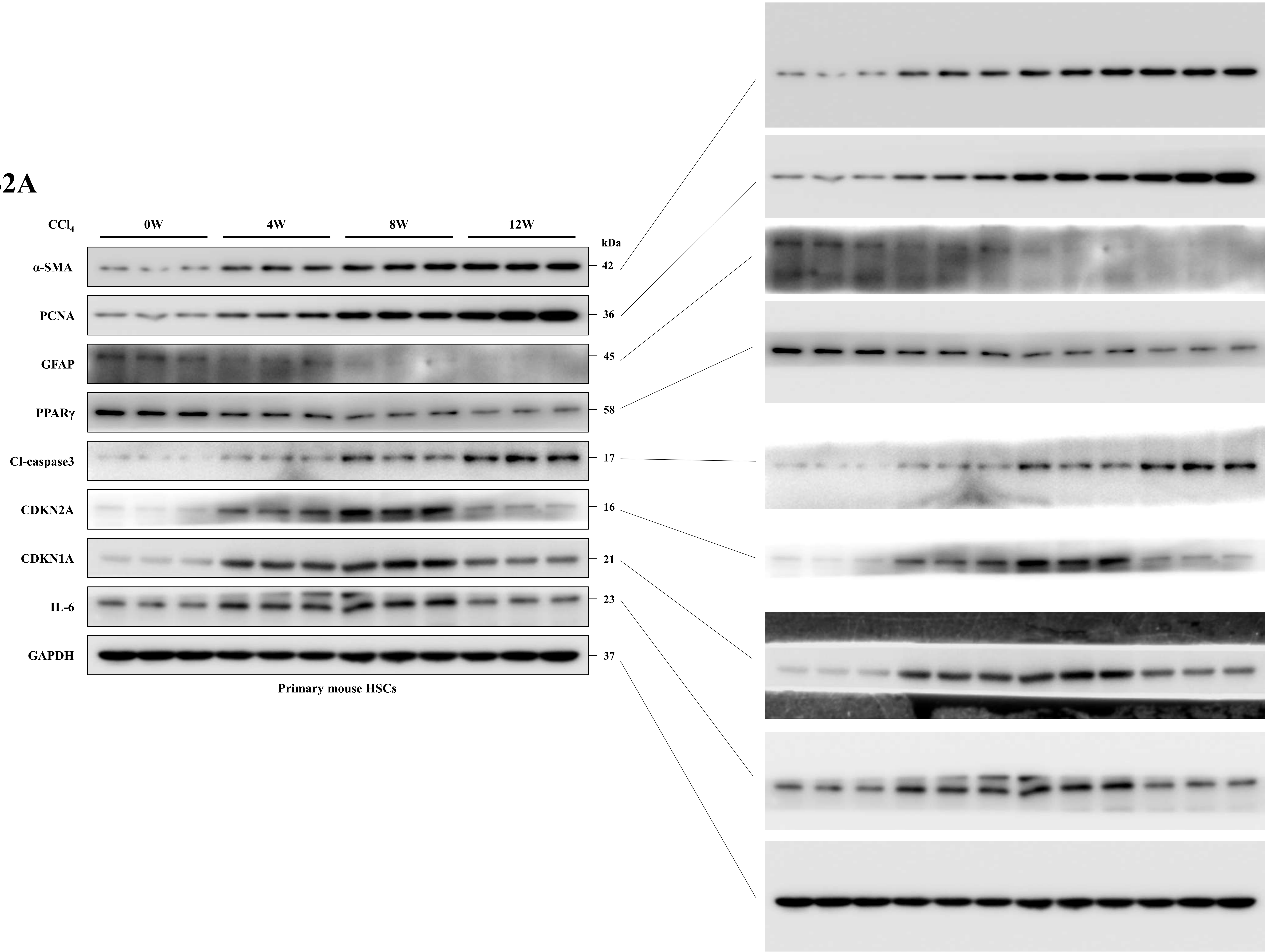
CCl4
0W
4W
8W
12W
kDa
α-SMA
42
PCNA
36
GFAP
45
PPARγ
58
Cl-caspase3
17
CDKN2A
16
CDKN1A
21
IL-6
23
GAPDH
37
Primary mouse HSCs

S4B

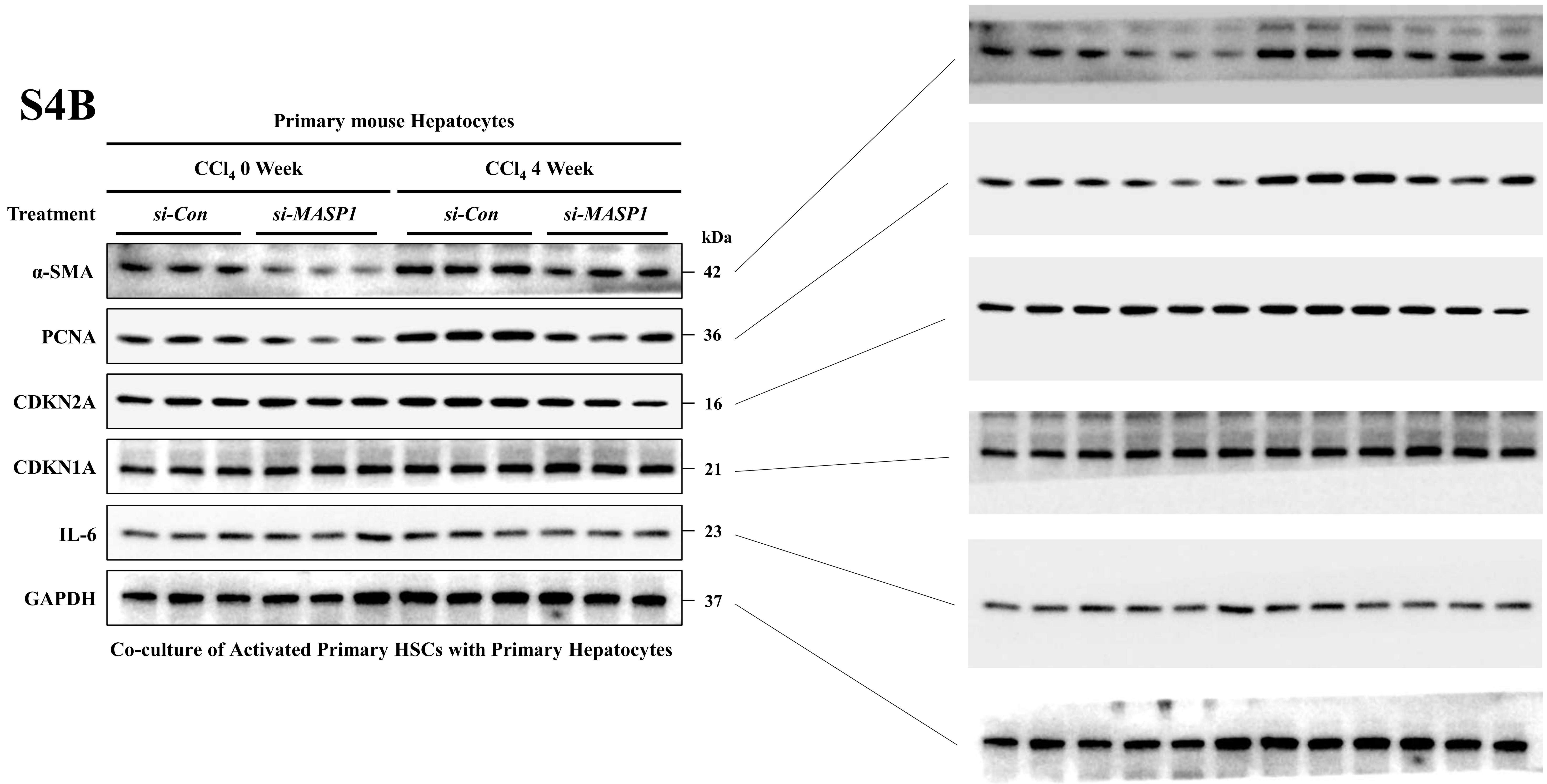

Primary mouse Hepatocytes
CCl4 0 Week
CCl4 4 Week
Treatment
si-Con
si-MASP1
si-Con
si-MASP1
kDa
α-SMA
42
PCNA
36
CDKN2A
16
CDKN1A
21
IL-6
23
GAPDH
37
Co-culture of Activated Primary HSCs with Primary Hepatocytes

S4C

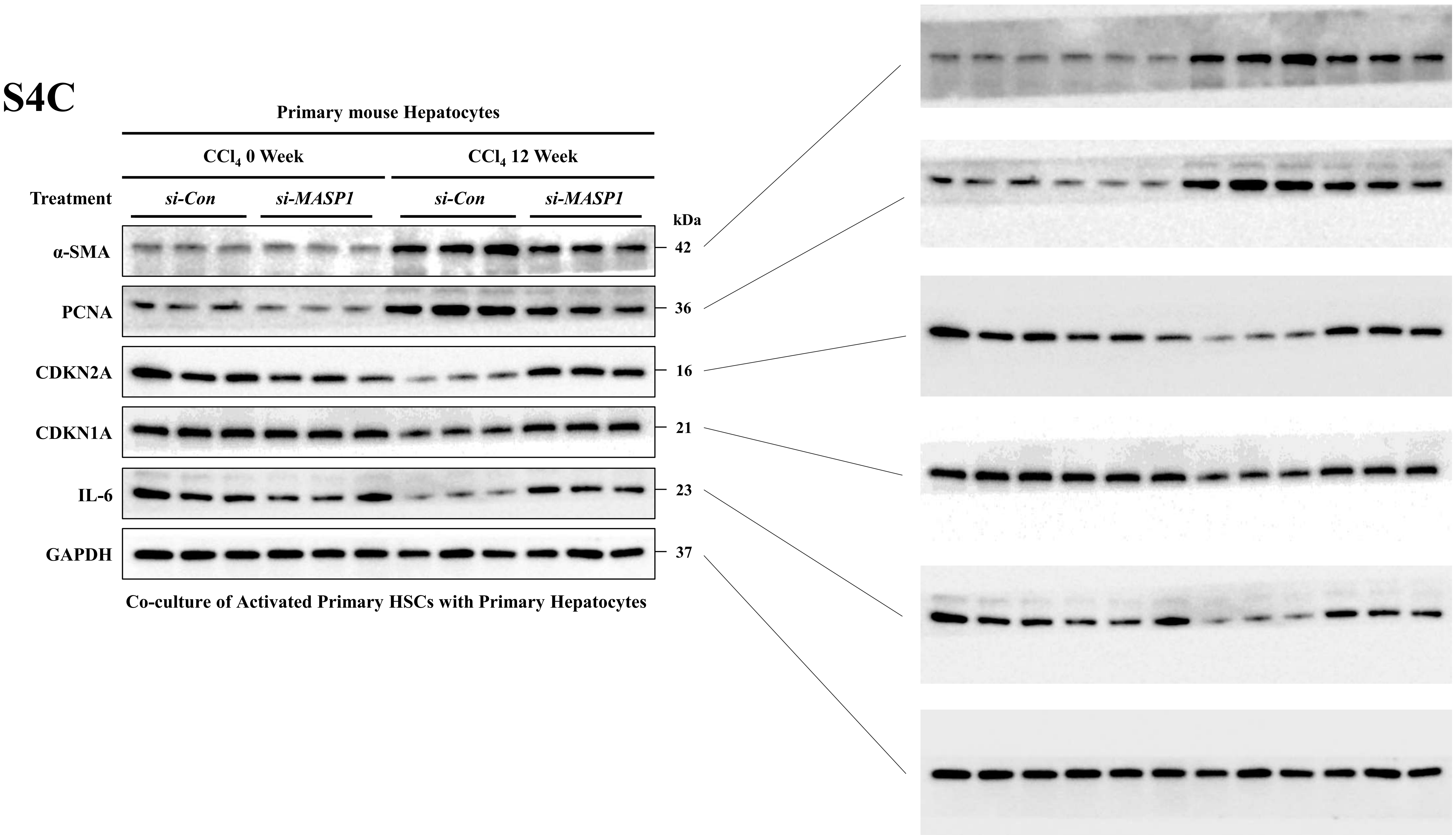

Primary mouse Hepatocytes
CCl4 0 Week
CCl4 12 Week
Treatment
si-Con
si-MASP1
si-Con
si-MASP1
kDa
α-SMA
42
PCNA
36
CDKN2A
16
CDKN1A
21
IL-6
23
GAPDH
37
Co-culture of Activated Primary HSCs with Primary Hepatocytes

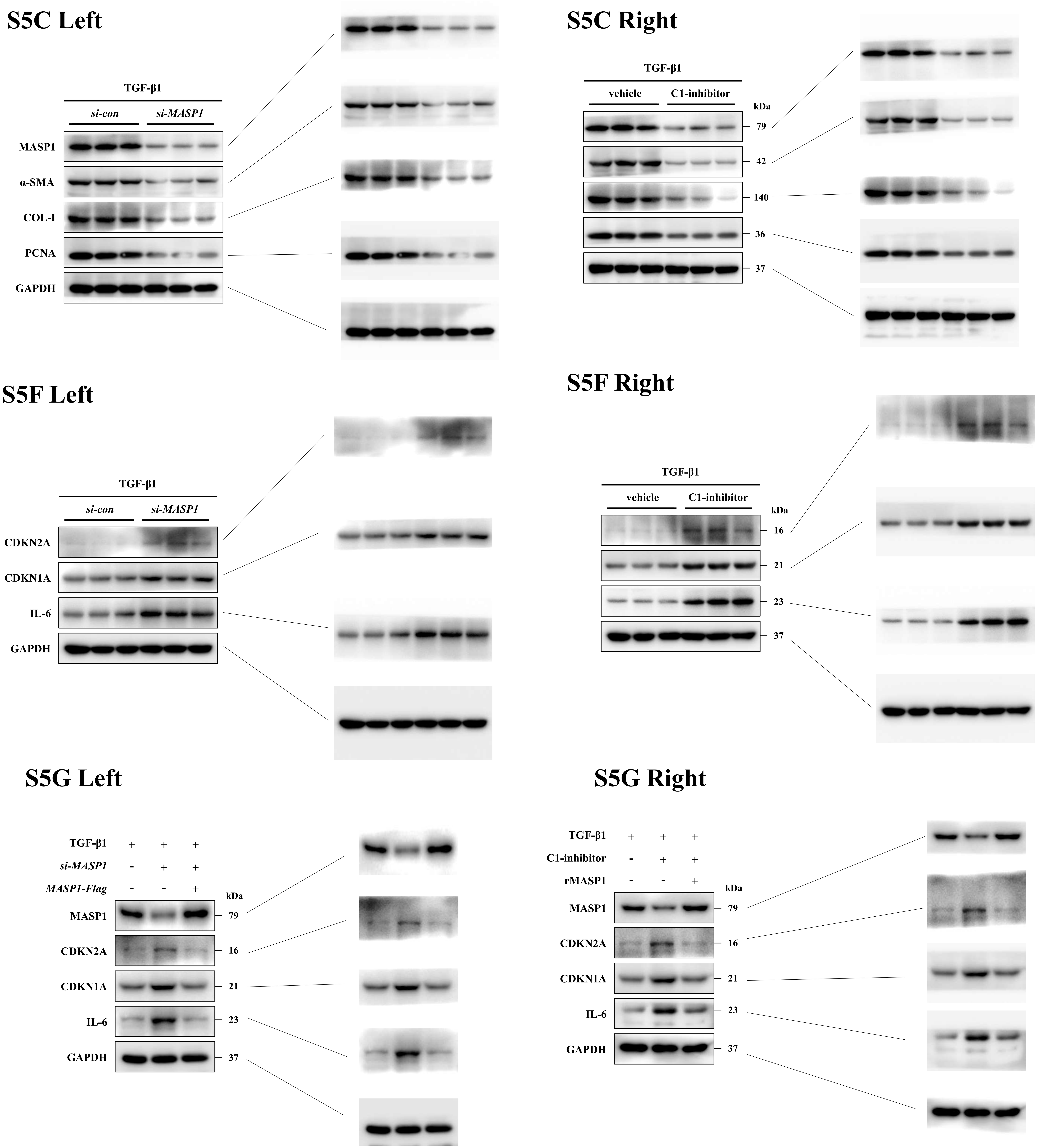

S5C Left
TGF-β1
si-con
si-MASP1
MASP1
α-SMA
COL-I
PCNA
GAPDH
S5C Right
TGF-β1
vehicle
C1-inhibitor
kDa
79
42
140
36
37
S5F Left
TGF-β1
si-con
si-MASP1
CDKN2A
CDKN1A
IL-6
GAPDH
S5F Right
TGF-β1
vehicle
C1-inhibitor
kDa
16
21
23
37
S5G Left
TGF-β1 + + +
si-MASP1 - + +
MASP1-Flag - - +
kDa
MASP1 79
CDKN2A 16
CDKN1A 21
IL-6 23
GAPDH 37
S5G Right
TGF-β1 + + +
C1-inhibitor - + +
rMASP1 - - +
kDa
MASP1 79
CDKN2A 16
CDKN1A 21
IL-6 23
GAPDH 37

S6B

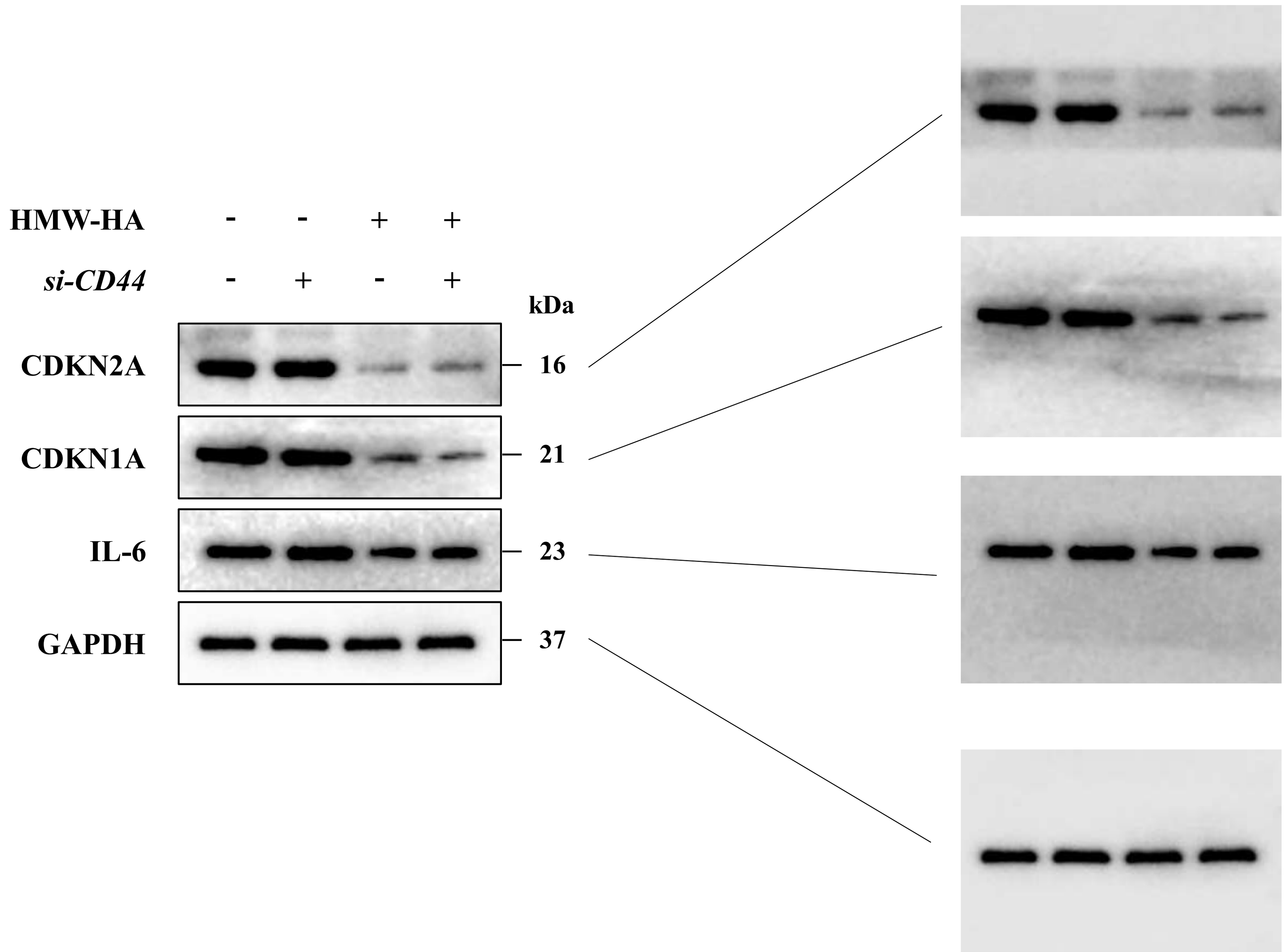

HMW-HA - - + +
si-CD44 - + - +
kDa
CDKN2A 16
CDKN1A 21
IL-6 23
GAPDH 37

S6C

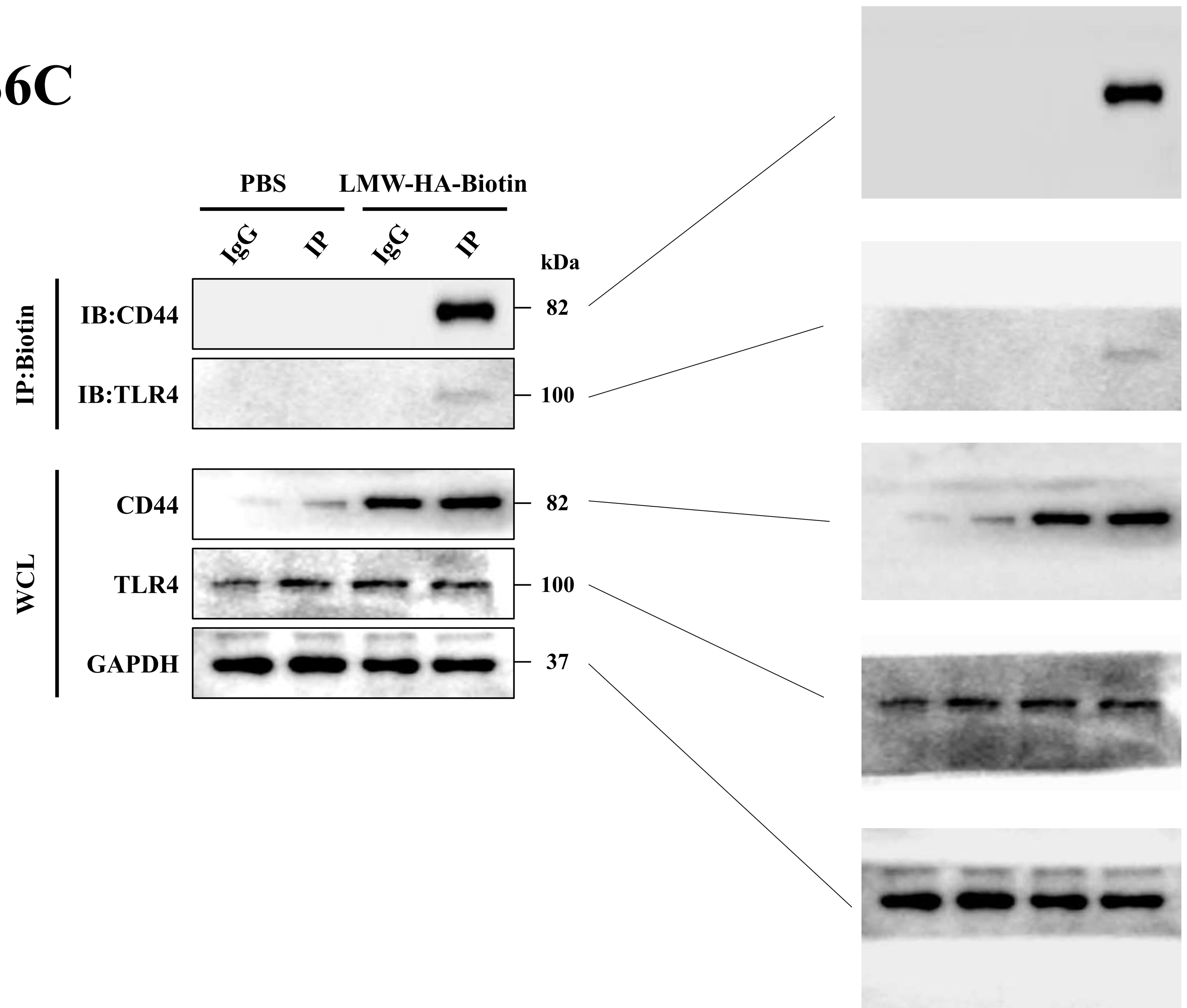

PBS
LMW-HA-Biotin
IgG IP IgG IP
kDa
IP:Biotin
IB:CD44 82
IB:TLR4 100
WCL
CD44 82
TLR4 100
GAPDH 37

S6D

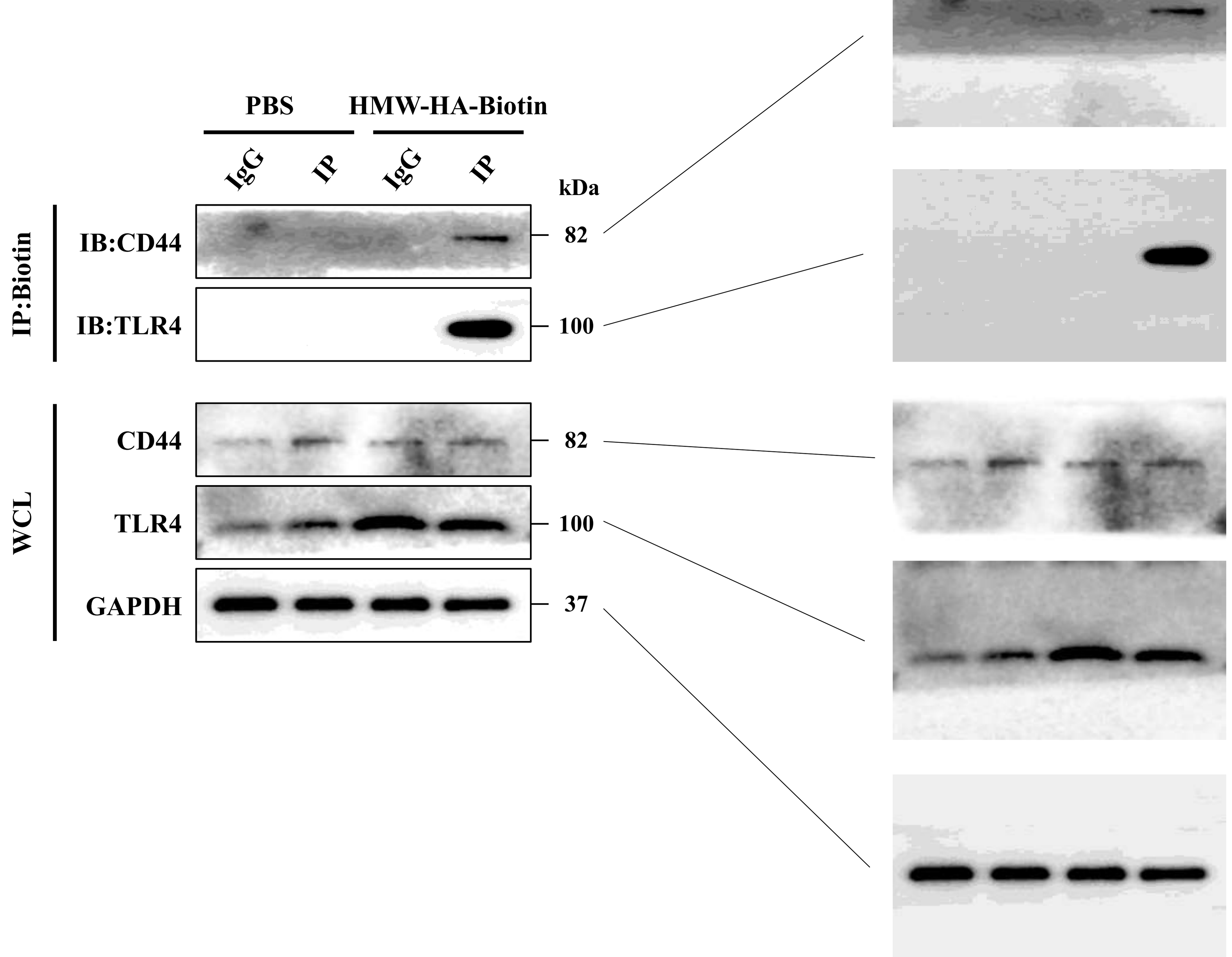

PBS
HMW-HA-Biotin
IgG IP IgG IP
kDa
IP:Biotin
IB:CD44 82
IB:TLR4 100
WCL
CD44 82
TLR4 100
GAPDH 37

S7A

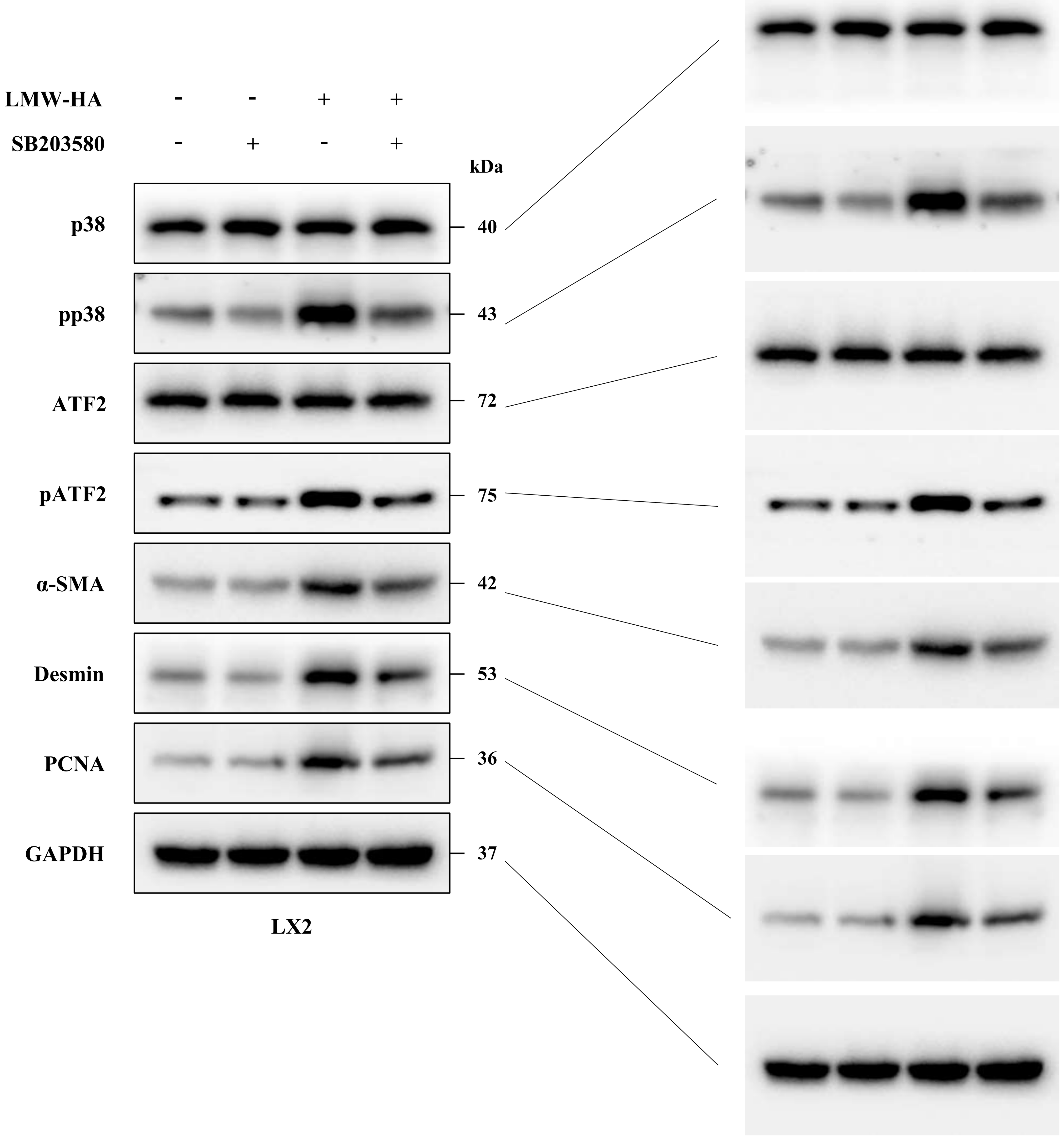
LMW-HA - - + +
SB203580 - + - +
kDa
p38
40
pp38
43
ATF2
72
pATF2
75
α-SMA
42
Desmin
53
PCNA
36
GAPDH
37
LX2

S7B

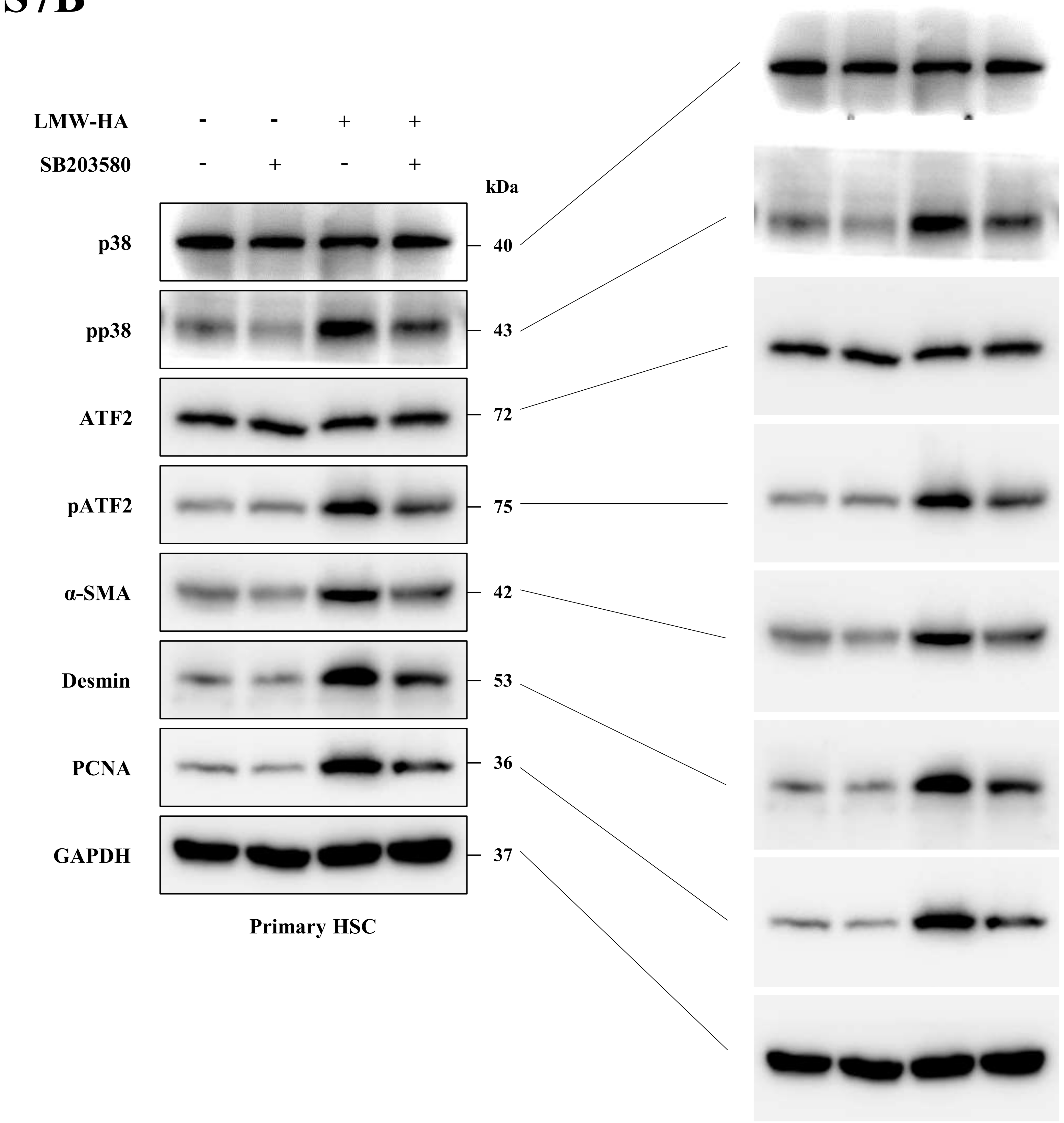
LMW-HA - - + +
SB203580 - + - +
kDa
p38
40
pp38
43
ATF2
72
pATF2
75
α-SMA
42
Desmin
53
PCNA
36
GAPDH
37
Primary HSC

S9H

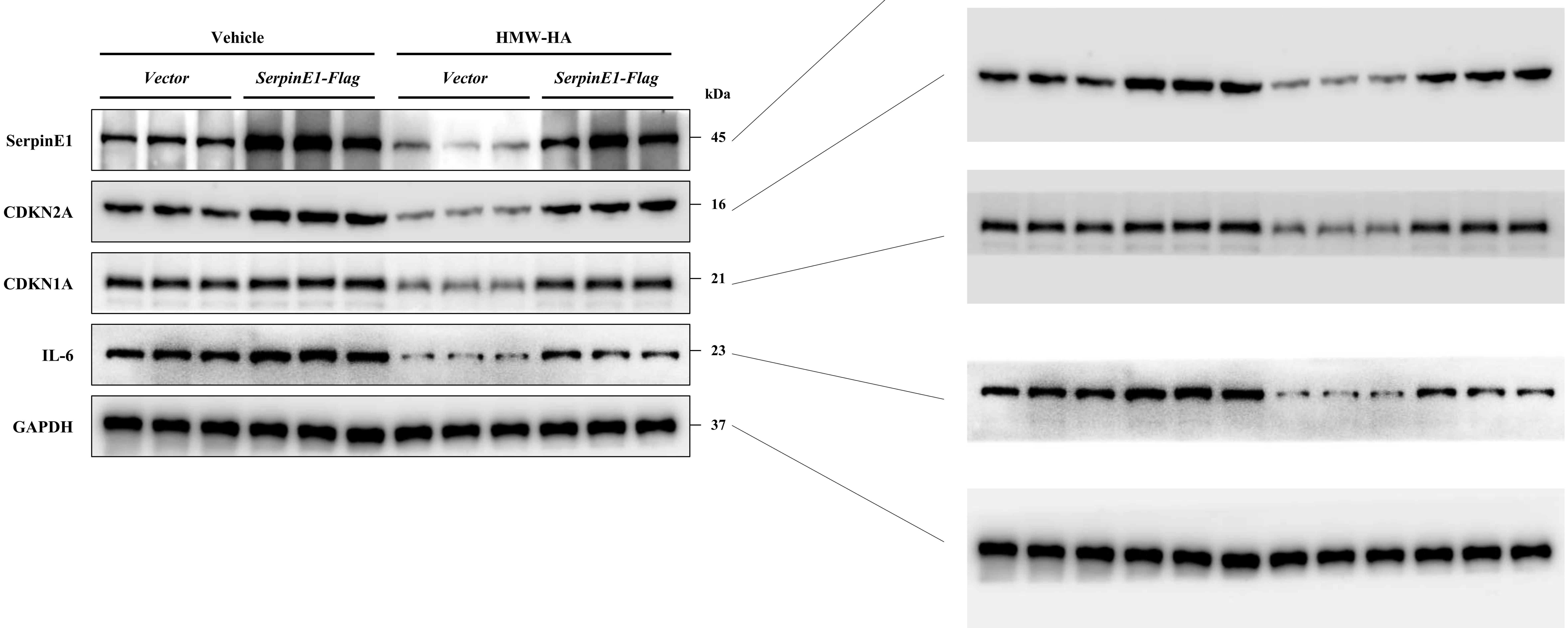
Vehicle
HMW-HA
Vector
SerpinE1-Flag
Vector
SerpinE1-Flag
kDa
SerpinE1
45
CDKN2A
16
CDKN1A
21
IL-6
23
GAPDH
37

S10A

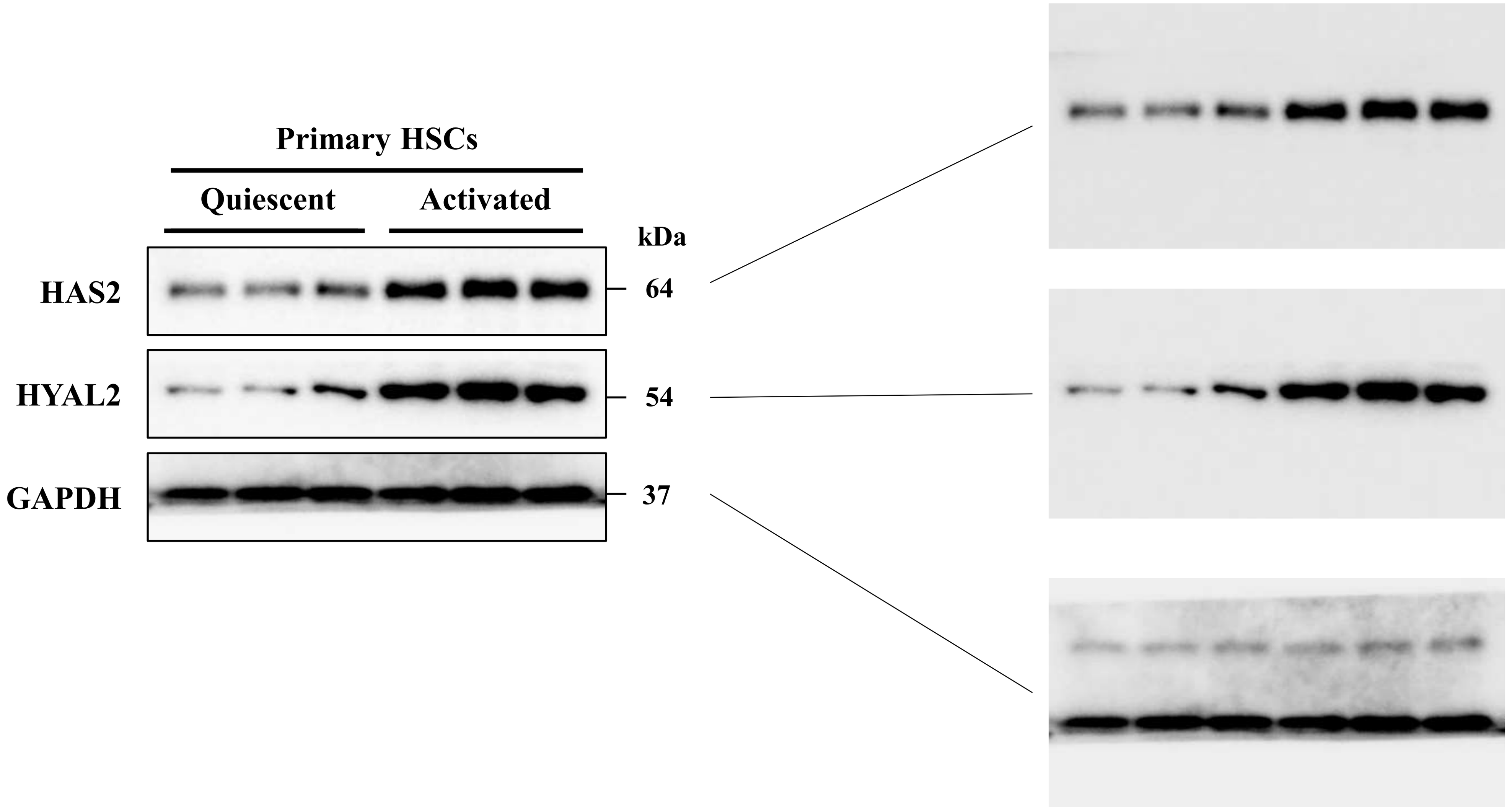

S10E

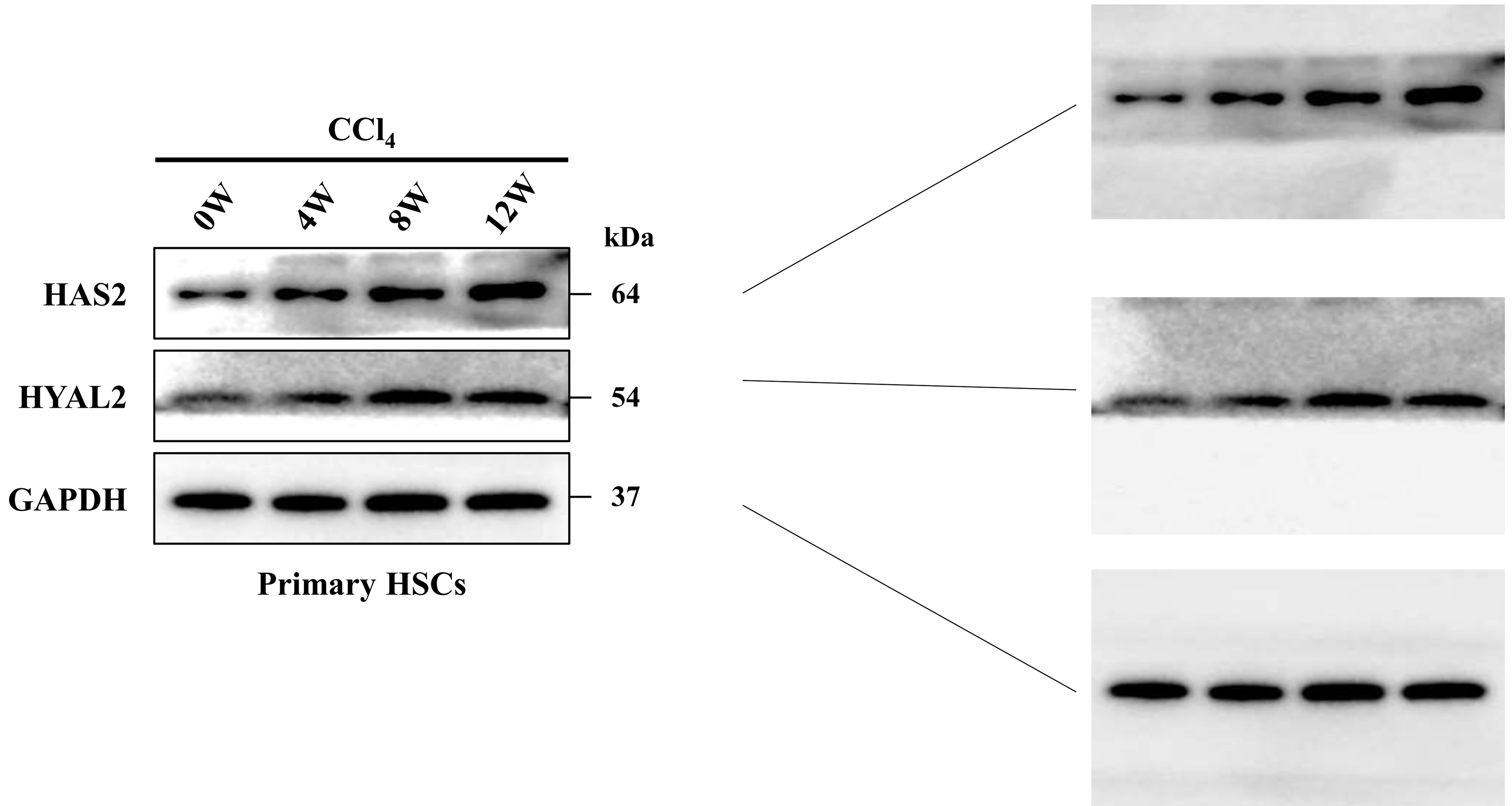

S10D

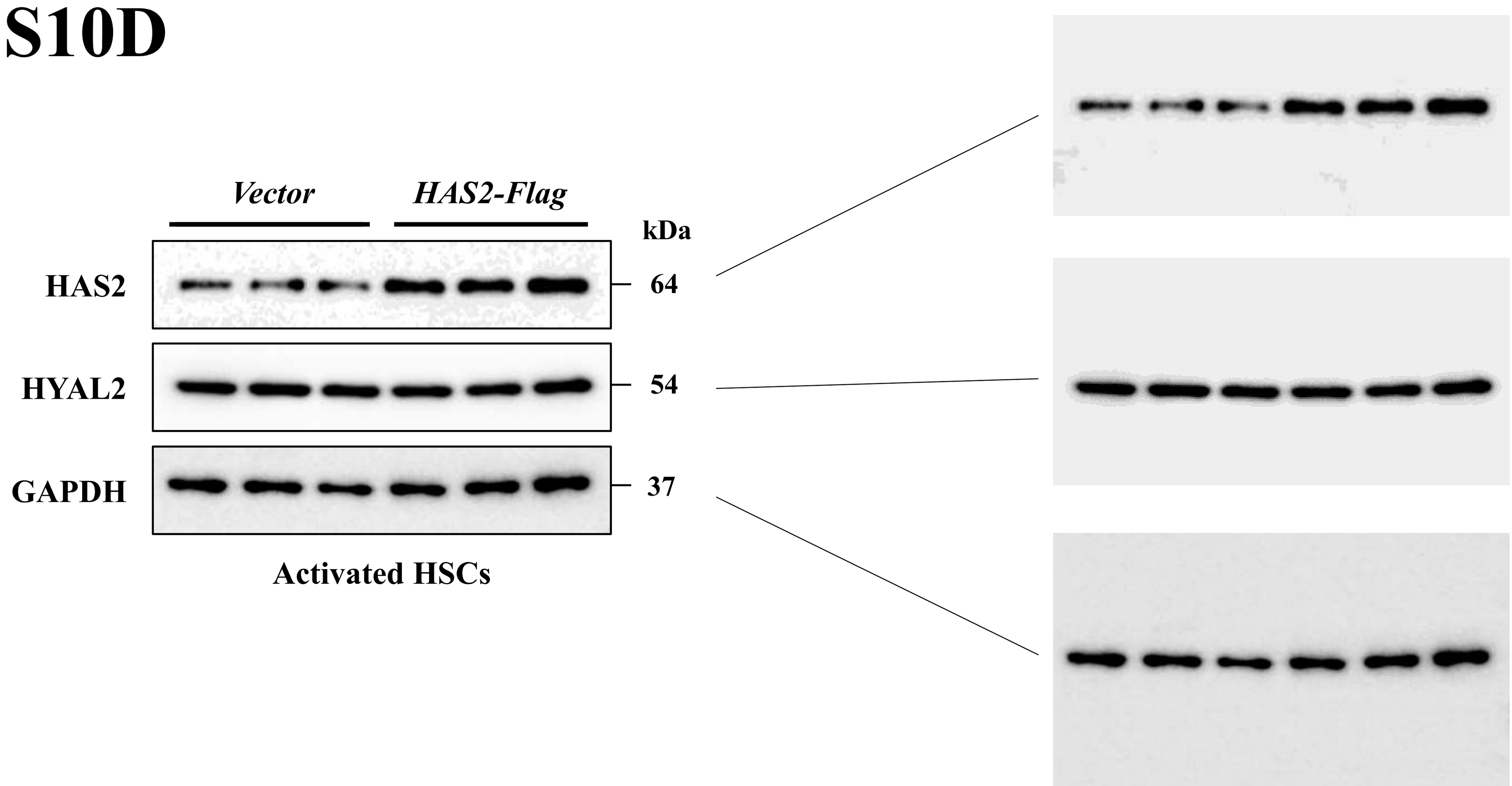

S10F

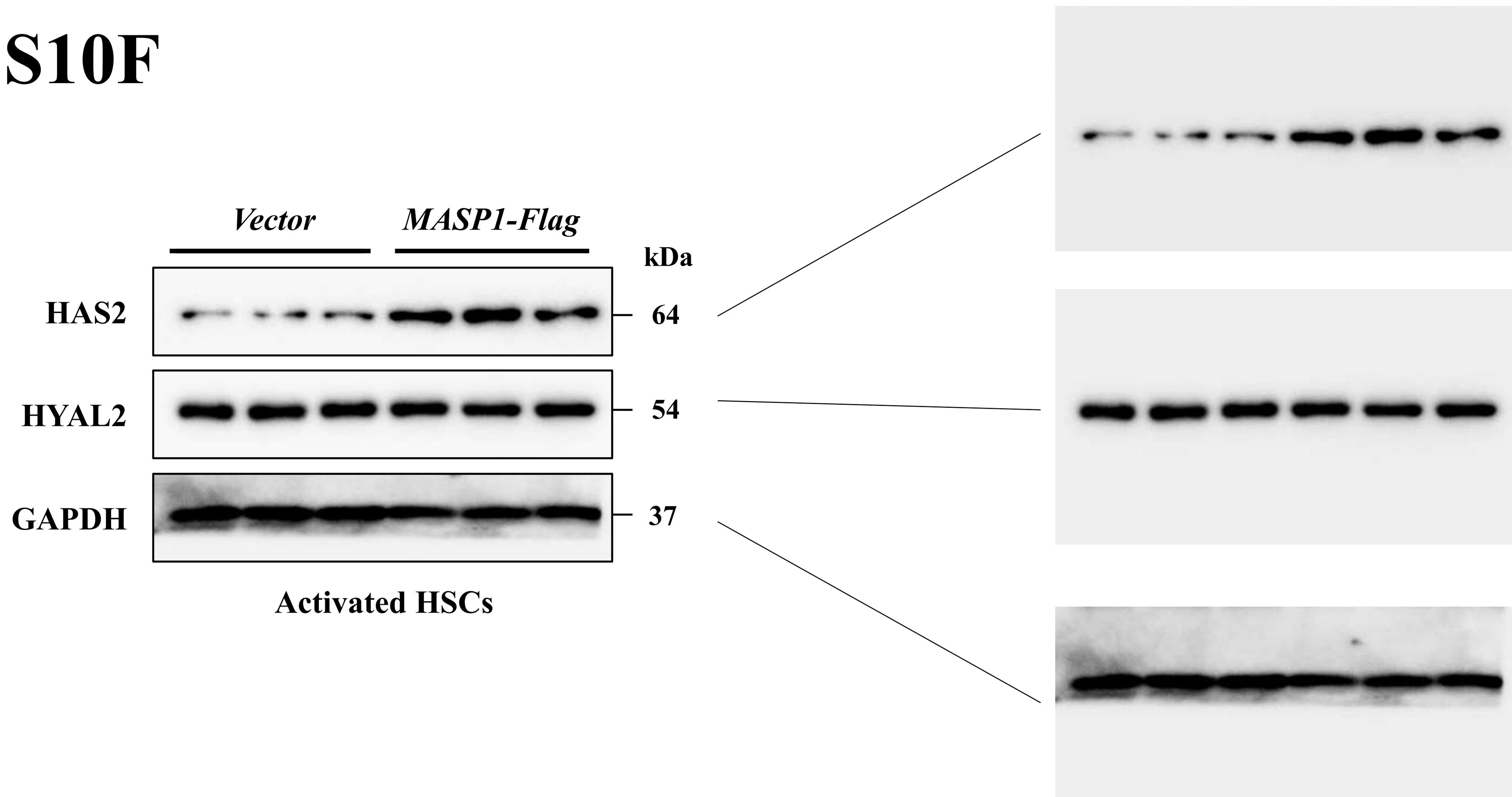

S11E

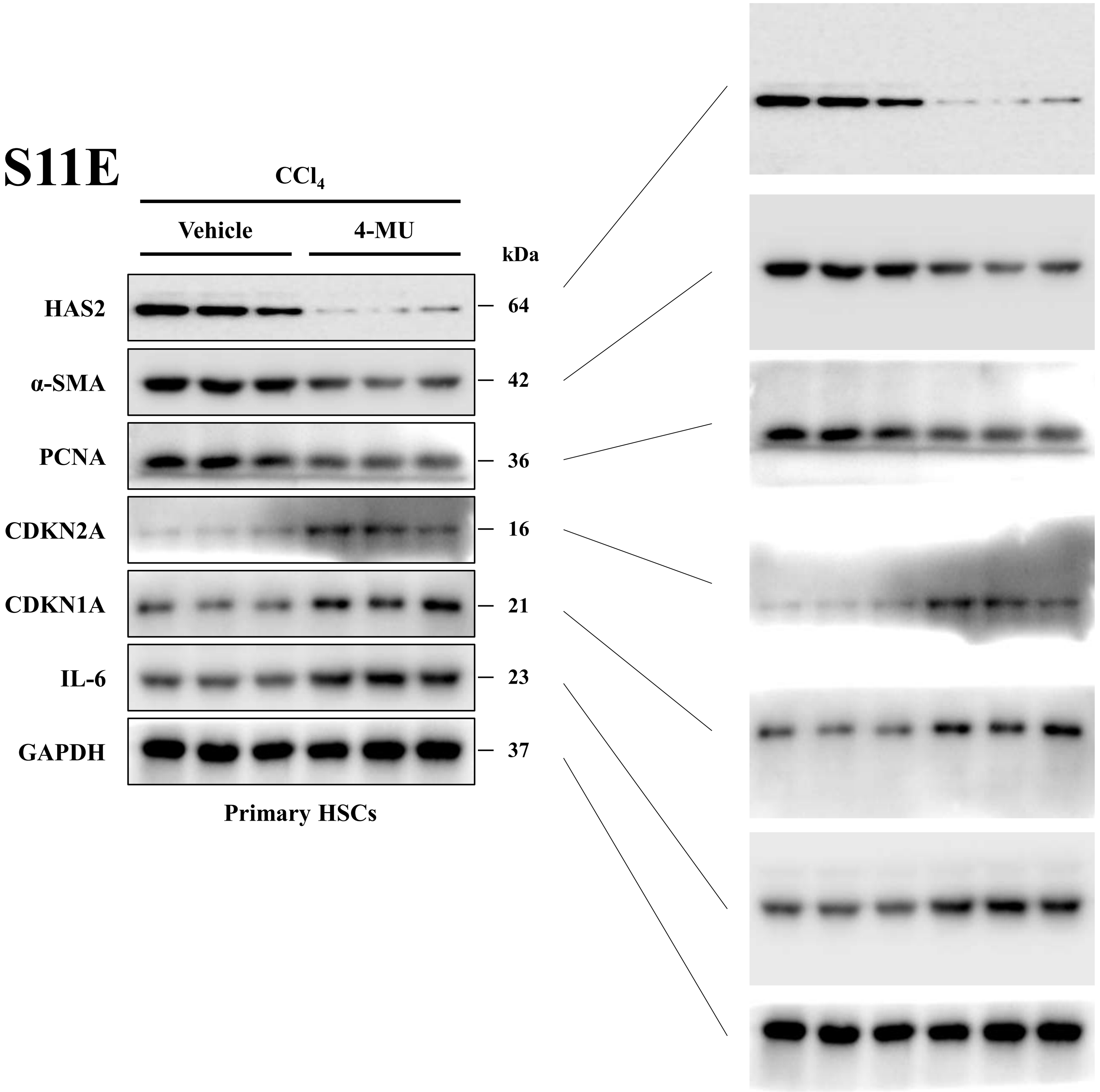
CCl4
Vehicle
4-MU
kDa
HAS2
64
α-SMA
42
PCNA
36
CDKN2A
16
CDKN1A
21
IL-6
23
GAPDH
37
Primary HSCs

S11G

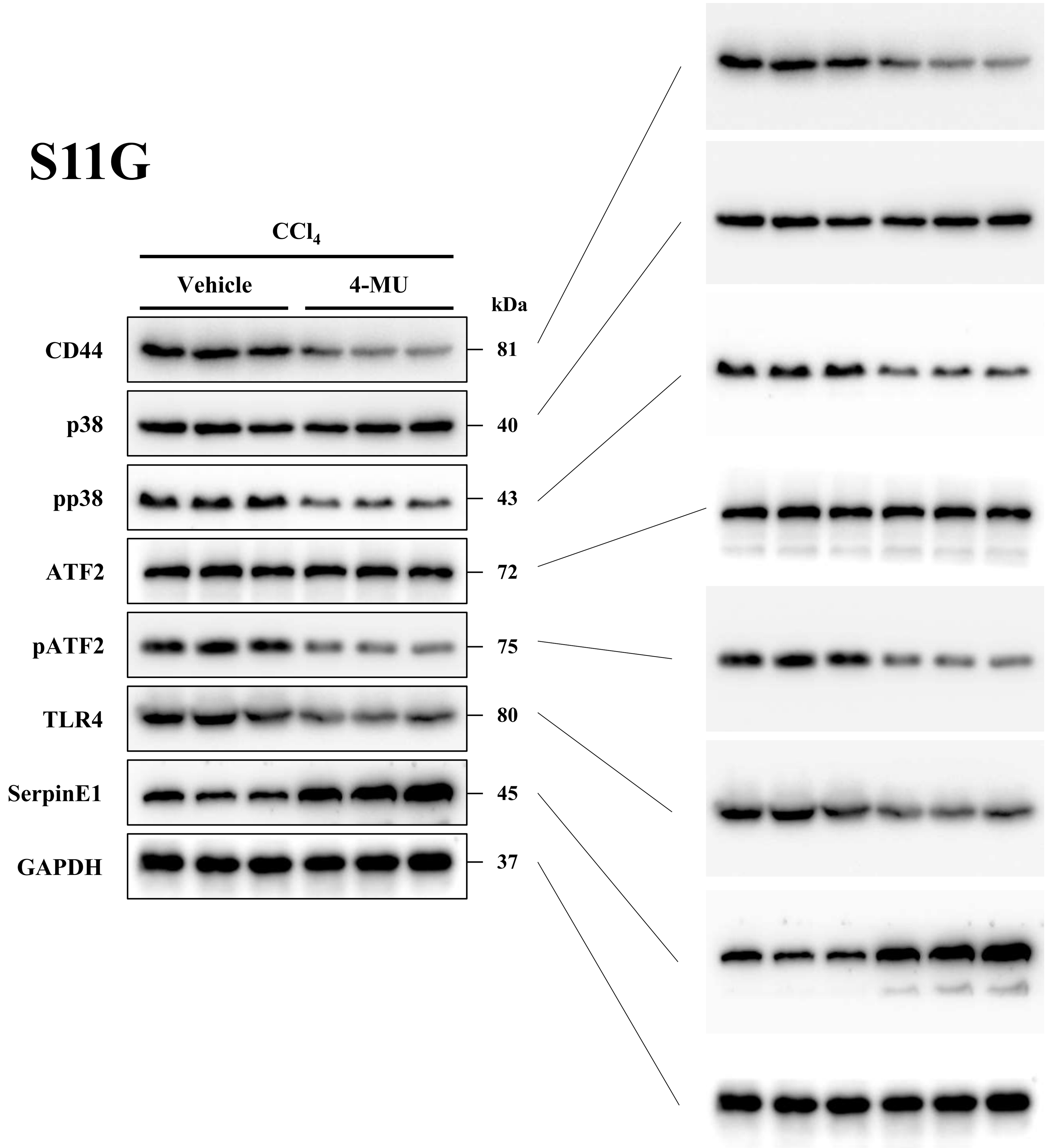
CCl4
Vehicle
4-MU
kDa
CD44
81
p38
40
pp38
43
ATF2
72
pATF2
75
TLR4
80
SerpinE1
45
GAPDH
37

S11J

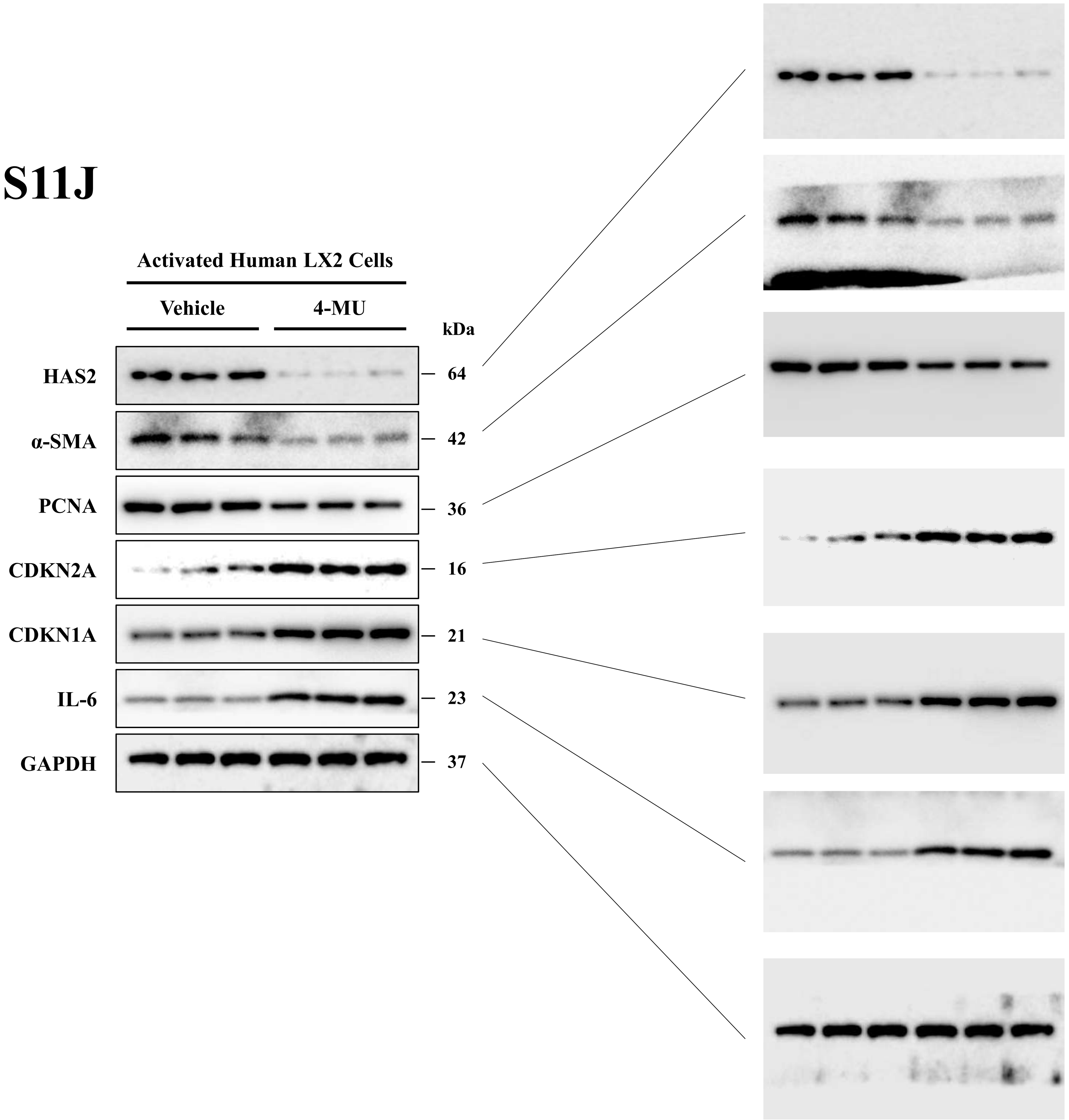
Activated Human LX2 Cells
Vehicle
4-MU
kDa
HAS2
α-SMA
PCNA
CDKN2A
CDKN1A
IL-6
GAPDH
64
42
36
16
21
23
37

S12D

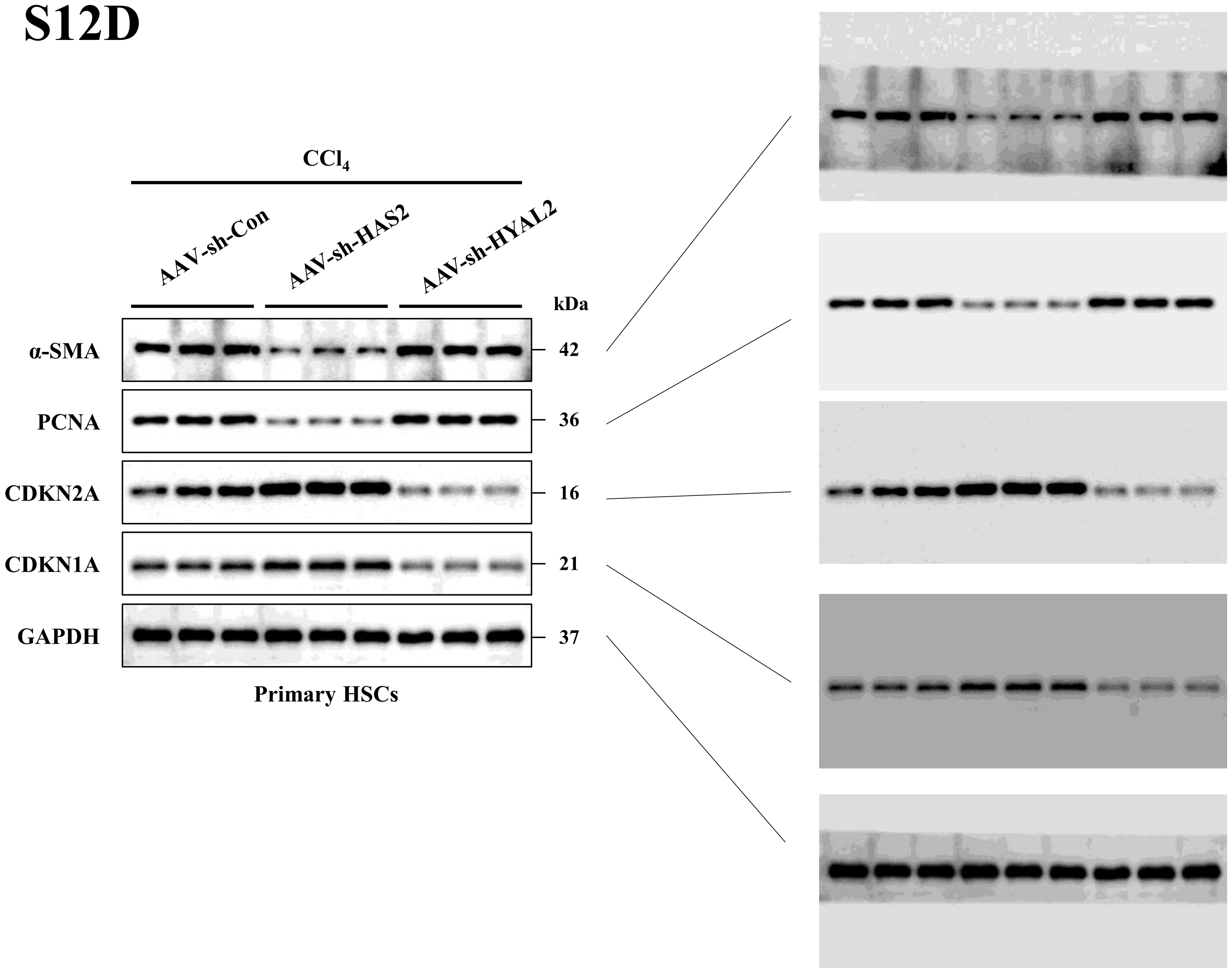
CCl4
AAV-sh-Con
AAV-sh-HAS2
AAV-sh-HYAL2
kDa
α-SMA
PCNA
CDKN2A
CDKN1A
GAPDH
42
36
16
21
37
Primary HSCs
