# Supplementary material for: MASP1‑METTL3‑HAS2 axis aggravates liver fibrosis through HMW‑HA‑mediated senescence inhibition and LMW‑HA‑induced activation of hepatic stellate cells
Source: Cell Death Dis. 2026 Aug 13;17(1):958. doi: 10.1038/s41419-026-09158-2 (PMC13752318; doi:10.1038/s41419-026-09158-2)
Supplement: Supplementary file 2 [file 41419_2026_9158_MOESM1_ESM.docx]

***Supplementary Information***

**MASP1‑METTL3‑HAS2 axis aggravates liver fibrosis through HMW‑HA‑mediated senescence inhibition and LMW‑HA‑induced activation of hepatic stellate cells**

**Running Title:** MASP1‑METTL3‑HAS2 axis aggravates liver fibrosis

**Xianzhi Liu**, ^1, #,^ * **Peng Wang**, ^5,^ ^#^ **Wei Zeng**, ^1, #^ **Huajie Liu**, ^1, #^ **Jun Lin**, ^3, #^ Wenxuan Gan, ^2^ Meilian Yu, ^1^ Yanyan Zheng, ^1^ Qian Luo, ^1^ Mingqing Zhang, ^1,^ * Mingkai Li, ^4,^ * Yihang Gong, ^6,^ * Weiling He, ^2,^ *

^1^Department of Gastroenterology, Xiang'an Hospital of Xiamen University, School of Medicine, Xiamen University, Xiamen, Fujian, 361000, China.

^2^Department of Gastrointestinal Surgery, Xiang'an Hospital of Xiamen University, School of Medicine, Xiamen University, Xiamen, Fujian, 361000, China.

^3^Department of Pediatrics, Xiang'an Hospital of Xiamen University, School of Medicine, Xiamen University, Xiamen, Fujian, 361000, China.

^4^Department of gastroenterology，Shenzhen Hospital of southern medical university, Shenzhen, 510086, China.

^5^Department of Gastroenterology, the Third Affiliated Hospital, Sun Yat-sen University. Guangzhou, 510630, China.

^6^Biotherapy Centre & Cell-gene Therapy Translational Medicine Research Centre, the Third Affiliated Hospital, Sun Yat-sen University. Guangzhou, 510630, China.

#These authors contributed equally.

*Corresponding author: liuxzh37@mail2.sysu.edu.cn; wlhe@xah.xmu.edu.cn; gongyh9@mail3.sysu.edu.cn; limingkai27@126.com; zmqing8084@sina.com.

**Figure legends**

**Figure 1. Analysis of HSC fate transformation during different fibrotic microenvironment.**

**(A)** Schematic diagram of the mouse modeling procedure.

**(B)** PKH26 fluorescence staining in HSCs. Scar bar: 50 μm, 25 μm.

**(C-D)** HSC activation induced by TGF‑β1; Data are presented as mean ± SEM; statistical significance was assessed by Student’s t-test (n = 3 per group), **P* < 0.05.

**(E-H)** Effects of the fibrotic microenvironment at different stages on HSC fate assessed by qPCR and immunofluorescence co‑localization; Data are presented as mean ± SEM; statistical significance was assessed by Student’s t-test (H, n = 3 per group), **P* < 0.05. Scar bar: 50 μm.

**Figure 2. MASP1 inhibits HSC senescence and promotes liver fibrosis.**

**(A-C)** Transcriptome sequencing reveals the association of MASP1 with HSC senescence and activation.

**(D-G)** MASP1 exacerbates liver fibrosis and injury in mice; Data are presented as mean ± SEM; statistical significance was assessed by Student’s t-test (D-E, n = 6 per group; F-G, n = 3 per group), **P* < 0.05. Scar bar: 50 μm.

**(H-K)** MASP1 promotes HSC activation and suppresses the expression of senescence markers; Data are presented as mean ± SEM; statistical significance was assessed by Student’s t-test (H, n = 6 per group; I-K, n = 3 per group), **P* < 0.05. Scar bar: 50 μm.

**(L)** MASP1 levels are significantly elevated in serum from patients and mice with advanced liver fibrosis. Data are presented as mean ± SEM (Human, n = 10 per group; Mouse, n = 6 per group).

**Figure 3.** **Targeted overexpression of CDKN2A in hepatic stellate cells reverses MASP1‑induced liver fibrosis.**

**(A)** Schematic diagram of the mouse model establishment.

**(B)** Immunofluorescence detection of GFP-labeled AAV6 vector and HSC marker Desmin colocalization. Scar bar: 50 μm.

**(C-D)** Mice were treated with rMASP1, *MASP1‑Luc*, and AAV‑OE‑CDKN2A; liver fibrosis was assessed by H&E, immunofluorescence, and Sirius Red staining. Data are presented as mean ± SEM; statistical significance was assessed by Student’s t-test (n = 6 per group), **P* < 0.05. Scar bar: 50 μm.

**(E-F)** Western blot analysis of fibrosis‑related protein expression in mice treated with rMASP1, *MASP1‑Luc*, and AAV‑OE‑CDKN2A. Data are presented as mean ± SEM; statistical significance was assessed by Student’s t-test (n = 3 per group), **P* < 0.05.

**(G)** SA‑β‑Gal staining for senescence detection in above groups. Data are presented as mean ± SEM; statistical significance was assessed by Student’s t-test (n = 6 per group), **P* < 0.05. Scar bar: 50 μm.

**(H-J)** Western blot and qPCR analysis of activation and senescence of primary HSCs isolated from mice in the AAV‑OE‑Con and AAV‑OE‑CDKN2A groups. Data are presented as mean ± SEM; statistical significance was assessed by Student’s t-test (n = 3 per group), **P* < 0.05.

**Figure 4.** **MASP1 promotes HSC activation and suppresses HSC senescence by regulation HA metabolism.**

**(A)** MASP1 is associated with glycosaminoglycan metabolism pathways.

**(B-C)** MASP1 promotes HA secretion from HSCs; Data are presented as mean ± SEM; statistical significance was assessed by Student’s t-test (n = 6 per group), **P* < 0.05.

**(D-E)** The levels of serum and liver total HA, HMW‑HA, LMW‑HA, and serum MASP1 were measured in mice and patients with liver fibrosis at different stages. Data are presented as mean ± SEM (Human, n = 10 per group; Mouse, n = 6 per group). And MASP1 level was positively correlated with HA level.

**(F-I)** LMW-HA promotes HSC activation via CD44; HMW-HA suppresses HSC senescence via TLR4; Data are presented as mean ± SEM by one-way ANOVA (n = 3-6 per group), **P* < 0.05. Scar bar: 25 μm.

**(J)** Schematic diagram of the regulatory mechanism regarding HA in HSC activation and senescence.

**Figure 5. LMW-HA and HMW-HA exacerbate liver fibrosis by promoting HSC activation and suppressing HSC senescence, respectively.**

**(A)** Schematic diagram of the mouse modeling procedure.

**(B-D)** LMW-HA and HMW-HA promote liver fibrosis and injury. Data are presented as mean ± SEM by one-way ANOVA (n = 6 per group), **P* < 0.05 versus PBS/CTRL group, ^#^*P* < 0.05 versus PBS/CCl_4_ group. Scar bar: 50 μm.

**(E)** LMW-HA and HMW-HA exacerbate liver fibrosis by promoting HSC activation and suppressing HSC senescence, respectively. Data are presented as mean ± SEM by one-way ANOVA (n = 3 per group), **P* < 0.05.

**(F)** LMW-HA and HMW-HA promote the mRNA levels of ACTA2, COL1A1, Timp1 and HAS2. Data are presented as mean ± SEM by one-way ANOVA (n = 3 per group), **P* < 0.05 versus PBS/CTRL group, ^#^*P* < 0.05 versus PBS/CCl_4_ group.

**(G-J)** LMW-HA and HMW-HA mediate their effects via the CD44-p38-ATF2 axis and the TLR4-SerpinE1 axis, respectively. n = 3-6 per group. Scar bar: 50 μm.

**Figure 6. MASP1 promotes HAS2 expression through METTL3-mediated m⁶A modification.**

**(A-B)** MASP1 increases HAS2 mRNA expression. Data are presented as mean ± SEM; statistical significance was assessed by Student’s t-test (n = 3 per group), **P* < 0.05.

**(C-E)** MASP1 elevates m^6^A methylation, while the MASP1 inhibitor C1-inhibitor and *si-MASP1* can reverse this increase. Data are presented as mean ± SEM; statistical significance was assessed by Student’s t-test or one-way ANOVA (n = 3 per group), **P* < 0.05.

**(F-J)** MASP1 promotes the expression of METTL3 and HAS2. Data are presented as mean ± SEM; statistical significance was assessed by Student’s t-test (n = 3-6 per group), **P* < 0.05. Scar bar: 25 μm.

**(K)** Immunofluorescence staining shows co-localization of METTL3 and HAS2. Scar bar: 25 μm.

**(L-M)** Inhibition of METTL3 reverses the MASP1-induced increase in m^6^A methylation. Data are presented as mean ± SEM by one-way ANOVA (n = 3 per group), **P* < 0.05.

**(N-O)** Inhibition of METTL3 reverses the MASP1-induced upregulation of HAS2 expression. Data are presented as mean ± SEM by one-way ANOVA (n = 3 per group), **P* < 0.05.

**(P-Q)** MASP1 binds to the promoter of METTL3. Data are presented as mean ± SEM; statistical significance was assessed by Student’s t-test or one-way ANOVA (n = 3 per group), **P* < 0.05.

**Figure 7. HSC-specific deficiency of METTL3 alleviates liver fibrosis by modulating HSC activation and senescence via the m^6^A/HAS2/HA pathway.**

**(A-B)** METTL3 deficiency reduces m^6^A methylation levels. Data are presented as mean ± SEM by one-way ANOVA (n = 3 per group), **P* < 0.05.

**(C-H)** METTL3 deficiency alleviates liver fibrosis, HA secretion, HAS2 expression and HSC activation. Data are presented as mean ± SEM by one-way ANOVA (n = 6 per group), **P* < 0.05. Scar bar: 50 μm.

**(I-L)** METTL3 deficiency promotes HSC senescence. Data are presented as mean ± SEM by one-way ANOVA (n = 3-6 per group), **P* < 0.05. Scar bar: 50 μm.

**Figure 8. Inhibition of MASP1 promotes HSC senescence and alleviates liver fibrosis by modulating the fibrotic microenvironment.**

**(A)** Schematic diagram of the mouse modeling procedure.

**(B-D)** Mice in the AAV-shMASP1 group exhibited reduced MASP1 expression. Data are presented as mean ± SEM by one-way ANOVA (n = 3-6 per group), **P* < 0.05.

**(E-G)** Knockdown of MASP1 alleviated liver fibrosis and injury. Data are presented as mean ± SEM by one-way ANOVA (n = 6 per group), **P* < 0.05. Scar bar: 50 μm.

**(H)** MASP1 knockdown decreased m^6^A methylation levels. Data are presented as mean ± SEM by one-way ANOVA (n = 6 per group), **P* < 0.05.

**(I-J)** MASP1 knockdown reduced HAS2 expression and HA level. Data are presented as mean ± SEM by one-way ANOVA (n = 3-6 per group), **P* < 0.05.

**(K)** MASP1 knockdown inhibited HSC activation and promoted HSC senescence. Data are presented as mean ± SEM by one-way ANOVA (n = 3 per group), **P* < 0.05.

**(L)** PKH26-labeled HSCs were also isolated from the indicated groups, compared to initial input HSCs, RT-qPCR showed that senescent HSCs were enhanced while activated HSCs were inhibited when MASP1 was inhibited. Data are presented as mean ± SEM by one-way ANOVA (n = 3 per group), **P* < 0.05.

**Supplementary Materials and Methods**

**Histological Analysis**

Collagen deposition was evaluated using Sirius Red staining. Further histopathological examination was performed on frozen or paraffin-embedded liver tissue sections, which included Hematoxylin and Eosin (H&E) staining, along with immunohistochemistry, immunofluorescence assays. All procedures were carried out according to well-established protocols detailed in prior methodologies[1, 2]. All antibodies are listed in Supplementary Table 1, including vendor and catalog numbers.

**Western Blot Analysis**

Total protein lysates prepared from tissue samples or cultured cells were subjected to immunoblotting according to established procedures[1, 2]. Membranes were probed with the indicated primary antibodies, followed by incubation with corresponding horseradish peroxidase (HRP)-conjugated secondary antibodies for signal detection. Protein band intensities were analyzed by densitometry and normalized to the expression level of GAPDH, which served as a loading control. All antibodies are listed in Supplementary Table 1, including vendor and catalog numbers.

**Quantitative Real-Time PCR (qPCR)**

Total RNA was extracted from tissues or cultured cells. RNA concentration and purity were determined spectrophotometrically. Complementary DNA (cDNA) was synthesized from equal amounts of RNA using a commercial reverse transcription kit with oligo (dT) and random primers, according to the manufacturer's protocol. Quantitative real-time PCR was performed using a SYBR Green-based master mix on a real-time PCR detection system. Gene expression levels were calculated and normalized to the expression of a housekeeping gene.

**Senescence-Associated β-Galactosidase (SA‑β‑Gal) Staining**

Cellular senescence was assessed using a SA‑β‑Gal staining kit according to the manufacturer’s protocol. In brief, cells were washed with phosphate‑buffered saline (PBS), fixed with 4% paraformaldehyde for 10-15 min at room temperature, and then washed twice with PBS. The fixed cells were incubated overnight at 37 °C (without CO₂) with freshly prepared staining solution containing X‑Gal at pH 6.0. After incubation, cells were washed with PBS and examined under a microscope.

**Dot Blot Assay**

Total RNA was denatured and spotted onto a positively charged nylon membrane. The RNA was then cross-linked to the membrane using UV light. After blocking with 5% non-fat milk, the membrane was incubated overnight at 4°C with a primary antibody specific for m^6^A. Following washes, the membrane was incubated with an HRP-conjugated secondary antibody. The signal was developed using an enhanced chemiluminescence (ECL) substrate and visualized with an imaging system. Equal RNA loading was verified by staining the membrane with 0.02% methylene blue in 0.3 M sodium acetate (pH 5.2).

**5‑Ethynyl‑2′‑deoxyuridine (EdU) Assay**

Cell proliferation was assessed using a commercial EdU assay kit according to the manufacturer’s instructions. In brief, cells were seeded in culture plates and grown to approximately 60-70% confluence. EdU solution was added directly to the culture medium at a final concentration of 10 μM and incubated for 2 h under normal growth conditions. Subsequently, cells were fixed with 4% paraformaldehyde for 15 min at room temperature and permeabilized with 0.5% Triton X‑100 for 10 min. After washing with PBS, the click reaction mixture (containing fluorescent azide) was applied and incubated for 30 min in the dark. Nuclei were counterstained with Hoechst. Images were acquired using a fluorescence microscope.

**CCK-8 Assay**

Cell proliferation and viability were also assessed using the CCK‑8 kit. Briefly, cells were seeded into 96‑well plates at a density of 3-5 × 10^3^ cells per well and allowed to adhere overnight. After the desired experimental treatment, 10 μL of CCK‑8 reagent was added to each well and incubated at 37 °C for 1-4 h. The absorbance at 450 nm was measured and cell viability was calculated as a percentage relative to the control group.

**Chromatin Immunoprecipitation (ChIP) Assay**

Protein-DNA interactions were analyzed using a ChIP assay kit according to the manufacturer's instructions, with minor modifications. Briefly, cells were cross-linked with 1% formaldehyde for 10 min at room temperature to fix protein-DNA complexes. The cross-linking reaction was quenched by adding glycine. Cells were then lysed, and chromatin was sheared into fragments of 200-500 bp by sonication on ice. The sheared chromatin was immunoprecipitated overnight at 4°C with rotation using a specific antibody against the protein of interest. Control samples were incubated with normal IgG. Protein A/G magnetic beads were added and incubated for an additional 2 h. The bead-bound complexes were washed sequentially with low-salt, high-salt, LiCl, and TE buffers. The cross-links were reversed by incubation at 65°C overnight in the presence of NaCl and proteinase K. Immunoprecipitated DNA was purified using a DNA purification kit. The enrichment of specific DNA regions was quantified by qPCR. The results were normalized to the input DNA and expressed as fold enrichment relative to the IgG control group.

**Dual-Luciferase Reporter Assay**

The putative promoter region of the gene of interest was cloned into the pGL3-Basic firefly luciferase reporter vector. Mutagenesis of potential binding sites was introduced using a site-directed mutagenesis kit. For each assay, cells were co‑transfected with the promoter‑reporter construct (or its mutant) and a Renilla luciferase control plasmid (pRL‑TK) using a lipid‑based transfection reagent. After 48 hours, cells were lysed and luciferase activity was measured sequentially using a dual‑luciferase reporter assay system on a microplate luminometer. Firefly luciferase activity was normalized to Renilla luciferase activity for each sample to control for transfection efficiency. Data are presented as fold‑change relative to the empty vector control group.

**HA quantification and molecular weight fractionation**

HA levels were determined using a commercial ELISA kit. To obtain size‑specific HA fractions, we adapted a previously reported protocol with minor modifications[3]. Samples were first digested with Pronase at 60°C for 4 hours and then heated at 100°C for 30 minutes. Subsequently, the digested samples were passed through molecular weight cutoff columns of 300 kDa. HA fragments with molecular weights below 300 kDa were collected in the flow‑through. Finally, HA concentrations in both unfiltered and filtered samples were measured using the same HA quantification ELISA Kit.

**Co-culture of HSCs with hepatocytes**

Primary mouse HSCs were isolated and seeded 50, 000 per transwell insert. After adherence, the insert was placed on the top of primary mouse hepatocytes (100, 000 cells per well) with different treatments, and for 24 h co-culture. Primary HSC lysates were harvested for further experiments.

**Supplementary Figure Legends**

**Figure S1. The proportion of senescent HSC subtype decreases in advanced liver fibrosis.**

**(A-C)** Phenotypic changes of HSCs in patient samples with hepatitis, mild liver fibrosis and severe cirrhosis. Data are presented as mean ± SEM by one-way ANOVA (n = 5 per group), **P* < 0.05. Scar bar: 25 μm.

**(D-F)** Phenotypic changes of HSCs in mouse samples at different stages of liver fibrosis. Data are presented as mean ± SEM by one-way ANOVA (n = 5 per group), **P* < 0.05. Scar bar: 25 μm.

**Figure S2. The proportion of senescent HSC subtype decreases in advanced liver fibrosis of mice.**

**(A-C)** Expression of senescence, activation, quiescence, apoptosis, inactivation, proliferation-associated markers during liver fibrosis process. Data are presented as mean ± SEM by one-way ANOVA (n = 3 per group), **P* < 0.05.

**Figure S3. Changes of HSC subtypes under TGF‑β1 stimulation.**

**(A-B)** Expression changes of HSC subtype markers were examined under TGF‑β1 stimulation with concentration gradients and time courses. Data are presented as mean ± SEM by one-way ANOVA (n = 3 per group), **P* < 0.05.

**Figure S4. Large amounts of hepatocyte-derived MASP1 in advanced liver fibrosis promote HSC activation and inhibit HSC senescence.**

**(A)** Primary hepatocytes were isolated from mice at different stages of liver fibrosis and treated with *si‑MASP1*. MASP1 levels were then measured. Data are presented as mean ± SEM; statistical significance was assessed by Student’s t-test (n = 6 per group), **P* < 0.05.

**(B‑E)** Primary hepatocytes were isolated from mice at different stages of liver fibrosis and then treated with *si‑MASP1*. These hepatocytes were then co‑cultured with activated primary mouse HSCs. Subsequently the levels of HSC senescence‑related and activation‑related markers were detected. Data are presented as mean ± SEM by one-way ANOVA (n = 3-6 per group), **P* < 0.05. Scar bar: 25 μm.

**Figure S5.** **Inhibiting MASP1 promotes HSC senescence and suppresses their activation.**

**(A-C)** MASP1 promotes HSC proliferation and activation. Data are presented as mean ± SEM; statistical significance was assessed by Student’s t-test (n = 3-6 per group), **P* < 0.05. Scar bar: 25 μm.

**(D-H)** MASP1 suppresses HSC senescence. Data are presented as mean ± SEM; statistical significance was assessed by Student’s t-test or one-way ANOVA (n = 3-6 per group), **P* < 0.05. Scar bar: 25 μm.

**Figure S6.** **CD44 and TLR4 are the primary targets of LMW-HA and HMW-HA respectively.**

**(A)** HSCs were treated with LMW-HA and *si-TLR4*, and HSC proliferation and activation were detected by EdU and α-SMA staining. Data are presented as mean ± SEM; statistical significance was assessed by one-way ANOVA (n = 6 per group), **P* < 0.05. ns, not significant. Scar bar: 25 μm.

**(B)** HSCs were treated with HMW-HA and *si-CD44*, and senescence‑related protein expression was detected. Data are presented as mean ± SEM; statistical significance was assessed by one-way ANOVA (n = 3 per group), **P* < 0.05. ns, not significant.

**(C-D)** The interactions of LMW-HA and HMW-HA with CD44 and TLR4 were examined.

**Figure S7. LMW-HA promotes HSC activation via the p38/ATF2 pathway.**

**(A-B)** WB results showing that LMW-HA activating the p38/ATF2 pathway and HSC activity, which was inhibited by SB203580. Data are presented as mean ± SEM by one-way ANOVA (n = 3 per group), **P* < 0.05.

**(C-D)** LMW-HA-induced HSC proliferation and activation were reversed by SB203580. Data are presented as mean ± SEM by one-way ANOVA (n = 6 per group), **P* < 0.05. Scar bar: 25 μm.

**Figure S8. Inhibition of HA synthesis triggers senescence and suppresses activation of HSCs.**

**(A-D)** GO, KEGG, and REACTOME enrichment analyses of the 4‑MU‑treated group versus the control group.

**(E-H)** Heatmap, volcano plot, REACTOME enrichment, and GSEA results of the 4‑MU‑treated group versus the control group.

**Figure S9. HMW-HA inhibits HSC senescence by suppressing SerpinE1.**

**(A-G)** Transcriptome sequencing analysis.

**(H-I)** SerpinE1 overexpression reversed the senescence-suppressing effect by HMW‑HA; Data are presented as mean ± SEM by one-way ANOVA (n = 3-6 per group), **P* < 0.05. Scar bar: 50 μm, 25 μm.

**(J)** SerpinE1 level was examined in liver tissue and serum samples from patients and mice at different stages of liver fibrosis; Data are presented as mean ± SEM (Human, n = 10 per group; Mouse, n = 6 per group).

**Figure S10. The MASP1-HAS2 axis promotes the accumulation of HMW-HA in activated-HSCs during advanced liver fibrosis.**

**(A)** Protein and mRNA levels of HAS2 and HYAL2 were detected in quiescent and activated primary HSCs. Data are presented as mean ± SEM; statistical significance was assessed by Student’s t-test (n = 3 per group), **P* < 0.05.

**(B-C)** HSCs were treated with rHYAL2 and *HAS2-Flag*. The levels of total HA, HMW-HA and LMW-HA were then measured. Data are presented as mean ± SEM; statistical significance was assessed by Student’s t-test (n = 6 per group), **P* < 0.05.

**(D)** HSCs were treated with *HAS2-Flag*. Protein and mRNA levels of HYAL2 were detected. Data are presented as mean ± SEM; statistical significance was assessed by Student’s t-test (n = 3 per group), **P* < 0.05.

**(E)** Primary HSCs were isolated from mice at different stages of liver fibrosis. Protein and mRNA levels of HAS2 and HYAL2 were detected. Data are presented as mean ± SEM; statistical significance was assessed by one-way ANOVA (n = 3 per group), **P* < 0.05.

**(F)** Activated HSCs were treated with *MASP1-Flag*. Protein and mRNA expression of HAS2 and HYAL2 were detected. Data are presented as mean ± SEM; statistical significance was assessed by Student’s t-test (n = 3 per group), **P* < 0.05.

**Figure S11. HA synthesis Inhibition by 4‑MU alleviates liver fibrosis via suppressing HSC activation and promoting HSC senescence.**

**(A)** Schematic diagram of the mouse modeling procedure.

**(B-D)** 4-MU significantly alleviates CCl₄-induced liver fibrosis and injury. Data are presented as mean ± SEM by one-way ANOVA (n = 6 per group), **P* < 0.05, ^#^*P* < 0.05. Scar bar: 50 μm.

**(E-F)** 4-MU inhibits HSC activation and promotes HSC senescence. Data are presented as mean ± SEM by Student’s t-test and one-way ANOVA (n = 3 per group), **P* < 0.05, ^#^*P* < 0.05.

**(G-I)** The activity of the CD44-p38-ATF2 and TLR4-SerpinE1 pathways under 4-MU treatment. Data are presented as mean ± SEM; statistical significance was assessed by Student’s t-test (n = 3 per group), **P* < 0.05. Scar bar: 50 μm.

**(J-K)** LX-2 cells were treated with 4-MU and the expression of HAS2, activation- and senescence- related markers were detected. Data are presented as mean ± SEM by Student’s t-test and one-way ANOVA (n = 3 per group), **P* < 0.05, ^#^*P* < 0.05.

**Figure S12. Effects of HAS2 and HYAL2 knockdown on HSC activation and senescence, HMW-HA and LMW-HA level, and liver fibrosis.**

**(A‑B)** Mice were treated with CCl_4_, AAV‑sh‑Con, AAV‑sh‑HAS2 or AAV‑sh‑HYAL2. The expression of HAS2 and HYAL2, liver injury and fibrosis were assessed. qPCR was used to detect the mRNA expression of fibrosis‑related and senescence‑related markers. Data are presented as mean ± SEM by one-way ANOVA (n =3-6 per group), **P* < 0.05. Scar bar: 50 μm.

**(C)** Mice were treated with CCl_4_, AAV‑sh‑Con, AAV‑sh‑HAS2 or AAV‑sh‑HYAL2. Primary HSCs were then isolated from each group and the levels of total HA, HMW‑HA and LMW‑HA were measured. Data are presented as mean ± SEM by one-way ANOVA (n = 6 per group), **P* < 0.05.

**(D)** Mice were treated with CCl₄, AAV‑sh‑Con, AAV‑sh‑HAS2 or AAV‑sh‑HYAL2. The expression of activation‑related and senescence‑related proteins was detected by western blotting. Data are presented as mean ± SEM by one-way ANOVA (n = 3 per group), **P* < 0.05.

**Supplementary Figures and Figure Legends**

**Figure S1**

**Figure S2**

**Figure S3**

**Figure S4**

**Figure S5**

**Figure S6**

**Figure S7**

**Figure S8**

**Figure S9**

**Figure S10**

**Figure S11**

**Figure S12**

**Supplementary Table 1：Antibody Information**

| **Antibody** | **Vendor** | **Catalog number** |
| --- | --- | --- |
| α-SMA | Proteintech | 14395-1-AP |
| α-SMA | Abcam | ab5694 |
| PCNA | Proteintech | 10205-2-AP |
| GAPDH | CST | 2118S |
| GAPDH | CST | 97166S |
| COL-Ⅳ | Proteintech | 19674-1-AP |
| COL-Ⅳ | Abcam | ab236640, ab6586 |
| COL-Ⅰ | Proteintech | 14695-1-AP |
| COL-Ⅰ | Abcam | ab233080 |
| CDKN2A | Proteintech | 10883-1-AP |
| CDKN2A | Huabio | HA723746 |
| CDKN1A | Proteintech | 28248-1-AP |
| CDKN1A | Huabio | HA500156 |
| IL-6 | Proteintech | 21865-1-AP, 26404-1-AP |
| Desmin | Proteintech | 16520-1-AP |
| Desmin | Santa Cruz | sc-23879 |
| CD44 | Huabio | HA722845 |
| TLR4 | Proteintech | 19811-1-AP |
| HAS2 | Huabio | 83204-2-RR |
| HAS2 | Abcam | ab140671 |
| p38 | CST | 8690 |
| pp38 | CST | 4511 |
| ATF2 | Proteintech | 14834-1-AP |
| pATF2 | CST | [24329](https://www.cellsignal.cn/products/primary-antibodies/phospho-atf-2-thr71-atf-7-thr53-antibody/24329?site-search-type=Products&N=4294956287&Ntt=p-atf2&fromPage=plp) |
| SerpinE1 | Proteintech | 66261-1-Ig |
| METTL3 | Proteintech | 15073-1-AP |
| METTL14 | Huabio | HA722910 |
| m6A | CST | 56593 |
| MASP1 | Proteintech | 21837-1-AP |
| MASP1 | Abcam | ab255814, ab255731, ab232945 |
| GFAP | Proteintech | 16825-1-AP |
| Ki67 | Proteintech | 27309-1-AP, 28074-1-AP |
| PPARγ | Proteintech | 16643-1-AP |
| Cleaved-Caspase 3 | Proteintech | 19677-1-AP |
| HYAL2 | Proteintech | 15115-1-AP |
| HYAL2 | Abcam | ab68608 |

**REFERENCES**

1.	Liu X, Tan S, Liu H, Jiang J, Wang X, Li L, et al. Hepatocyte-derived MASP1-enriched small extracellular vesicles activate HSCs to promote liver fibrosis. Hepatology. 2023;77:1181-97.

2.	Tan S, Lu Y, Xu M, Huang X, Liu H, Jiang J, et al. β-Arrestin1 enhances liver fibrosis through autophagy-mediated Snail signaling. FASEB J. 2019;33:2000-16.

3.	Yang YM, Noureddin M, Liu C, Ohashi K, Kim SY, Ramnath D, et al. Hyaluronan synthase 2-mediated hyaluronan production mediates Notch1 activation and liver fibrosis. Sci Transl Med. 2019;11
